# Genomic expression catalogue of a global collection of BCG vaccine strains show evidence for highly diverged metabolic and cell-wall adaptations

**DOI:** 10.1038/srep15443

**Published:** 2015-10-21

**Authors:** Abdallah M. Abdallah, Grant A. Hill-Cawthorne, Thomas D. Otto, Francesc Coll, José Afonso Guerra-Assunção, Ge Gao, Raeece Naeem, Hifzur Ansari, Tareq B. Malas, Sabir A. Adroub, Theo Verboom, Roy Ummels, Huoming Zhang, Aswini Kumar Panigrahi, Ruth McNerney, Roland Brosch, Taane G. Clark, Marcel A. Behr, Wilbert Bitter, Arnab Pain

**Affiliations:** 1 Pathogen Genomics Group, Biological, Environmental Sciences and Engineering Division, King Abdullah University of Science and Technology, Thuwal, 23955-6900, Kingdom of Saudi Arabia; 2 Marie Bashir Institute for Infectious Diseases and Biosecurity and School and Public Health, University of Sydney, NSW 2006, Australia; 3 Pathogen Genomics, The Wellcome Trust Sanger Institute, Hinxton, Cambridge CB10 1SA, United Kingdom; 4 Faculty of Infectious and Tropical Diseases, London School of Hygiene & Tropical Medicine, London, United Kingdom; 5 Computational Bioscience Research Center (CBRC), King Abdullah University of Science and Technology (KAUST), Thuwal, 23955-6900, Jeddah, Kingdom of Saudi Arabia; 6 Department of Medical Microbiology and Infection Control, VU University Medical Center, 1081 BT Amsterdam, The Netherlands; 7 Bioscience Core Laboratory, King Abdullah University of Science and Technology, Thuwal, 23955-6900, Kingdom of Saudi Arabia; 8 Institut Pasteur, Unit for Integrated Mycobacterial Pathogenomics, Paris, France; 9 Faculty of Epidemiology and Population Health, London School of Hygiene and Tropical Medicine, London, United Kingdom.; 10 Department of Medicine, McGill University Health Centre, Montreal, QC, Canada; 11 Global Station for Zoonosis Control, Global Institution for Collaborative Research and Education (GI-CoRE), Hokkaido University, Sapporo, 001-0020, Japan

## Abstract

Although Bacillus Calmette-Guérin (BCG) vaccines against tuberculosis have been available for more than 90 years, their effectiveness has been hindered by variable protective efficacy and a lack of lasting memory responses. One factor contributing to this variability may be the diversity of the BCG strains that are used around the world, in part from genomic changes accumulated during vaccine production and their resulting differences in gene expression. We have compared the genomes and transcriptomes of a global collection of fourteen of the most widely used BCG strains at single base-pair resolution. We have also used quantitative proteomics to identify key differences in expression of proteins across five representative BCG strains of the four tandem duplication (DU) groups. We provide a comprehensive map of single nucleotide polymorphisms (SNPs), copy number variation and insertions and deletions (indels) across fourteen BCG strains. Genome-wide SNP characterization allowed the construction of a new and robust phylogenic genealogy of BCG strains. Transcriptional and proteomic profiling revealed a metabolic remodeling in BCG strains that may be reflected by altered immunogenicity and possibly vaccine efficacy. Together, these integrated-omic data represent the most comprehensive catalogue of genetic variation across a global collection of BCG strains.

Tuberculosis disease continues to be one of the world’s leading causes of morbidity and mortality by a single infectious agent. The global spread of drug-resistant forms of tuberculosis and the effect of HIV-*Mycobacterium tuberculosis* co-infection in many parts of the world have revived the efforts to develop a new vaccine. The currently licensed tuberculosis vaccine, attenuated *Mycobacterium bovis* strain Bacillus Calmette-Guérin (BCG), was originally derived from *M. bovis*, a virulent member of the *M. tuberculosis* complex that mainly affects wild and domesticated mammals^1^,^2^, by repeated passages on potato slices soaked in glycerol-ox bile. Since its introduction in 1921, BCG has been used for nearly a century to immunize over 3 billion people in at least 180 countries^3^. It was noted as far back as 1967 that its protective efficacy against tuberculosis disease varies substantially between studies, showing an average risk reduction of pulmonary tuberculosis of 50% and of disseminated and meningitic forms of this disease by 70 to 80%^4^,^5^,^6^. There are several possible explanations for this variable efficacy, including prior exposure to non-tuberculous mycobacteria that could block or mask BCG vaccination-induced immune responses, the nutritional or genetic background of the human population, and variations between the *M. tuberculosis* lineages present in the world^6^,^7^,^8^,^9^.

Another reason for variation in BCG effectiveness could be differences within and between vaccine strains, with potentially considerable heterogeneity among these strains^10^. Today, fourteen BCG strains are currently used worldwide as vaccines against tuberculosis. Early clinical trials in indigenous groups in North America, infants in Chicago and school children in the UK demonstrated the efficacy of the vaccine and led to its distribution to several countries for worldwide application^11^,^12^. The strains used are all progenies of the original strain attenuated by Calmette and Guérin during 1909–1921. In the absence of lyophilisation or freezing and the production of seed-lots until the 1960 s, the propagation of BCG through continuous passage under different laboratory conditions resulted in the generation of daughter BCG strains with different morphological, biochemical and immunological characteristics^13^,^14^. Some of these daughter strains have lost genomic regions that affect their antigenic content, potentially changing their protective efficacy^15^,^16^. Several studies on BCG strains have demonstrated changes at the genome level and comparative genomic studies have uncovered regions of difference (RD) including single nucleotide polymorphisms (SNPs), insertion sequences (*IS*6110), deletions and tandem duplications^16^,^17^,^18^,^19^,^20^. Accordingly, BCG strains are sub-classified into early strains represented by BCG Russia, Japan, Moreau, Birkhaug and Sweden that show fewer chromosomal deletions than late strains, such as BCG Prague, Glaxo, Danish, Tice, Frappier, Connaught, Phipps and Pasteur^21^.

The most attributed reason for the primary attenuation of BCG compared to *M. bovis* is the loss of the RD1 locus, which is deleted in all BCG daughter strains and affects the protein secretion pathway ESX-1 system^13^,^19^,^22^. Although, it has been shown that deletion of RD1 in *M. tuberculosis* leads to attenuation of this strain^23^, complementation of BCG with this region does not fully restore virulence to wild-type levels^24^, suggesting that other attenuating mutations may have occurred^25^. A previous study suggested that the RDs described to date, including RD2, which is absent from late strains and retained in the early strains, might not correlate with the protective efficacy of BCG strains in mice^26^. In a randomized controlled trial in humans BCG Danish (a late strain) and BCG Japan (an early strain) produced higher proportions of polyfunctional CD4^+^T cells than BCG Russia (also an early strain) and BCG Japan also led to higher concentrations of secreted Th1 cytokines than either of the other two vaccines^27^.

While BCG was developed as a vaccine against *M. tuberculosis* infection, since 1976 it has also been used as adjunct therapy for the treatment of bladder cancer and has found favour for the treatment of advanced malignant melanoma^28^,^29^. For bladder cancer it is highly effective with a recent meta-analysis demonstrating a 32% reduction in the risk of recurrence with BCG immunotherapy compared to mitomycin C chemotherapy^30^. Minor adverse events are relatively common and usually as a result of localized inflammatory effects^31^. However, potentially fatal sepsis resulting from dissemination of the live bacteria occurs rarely^32^. A number of the BCG strains are licensed for use in bladder cancer, which, however, shows variability in efficacy^33^.

With differing BCG vaccines strains being used for immunisation and the treatment of bladder cancer, it is important to better understand the genetic basis underpinning this evolution and elucidate the reasons for variations in levels of protection induced by different BCG daughter strains. We, therefore, undertook a systematic and comparative survey of the BCG strains with respect to their genomes, transcriptomes and expressed proteins.

## Results

### Whole-genome sequencing and phylogeny of BCG strains

**Figure 1:**
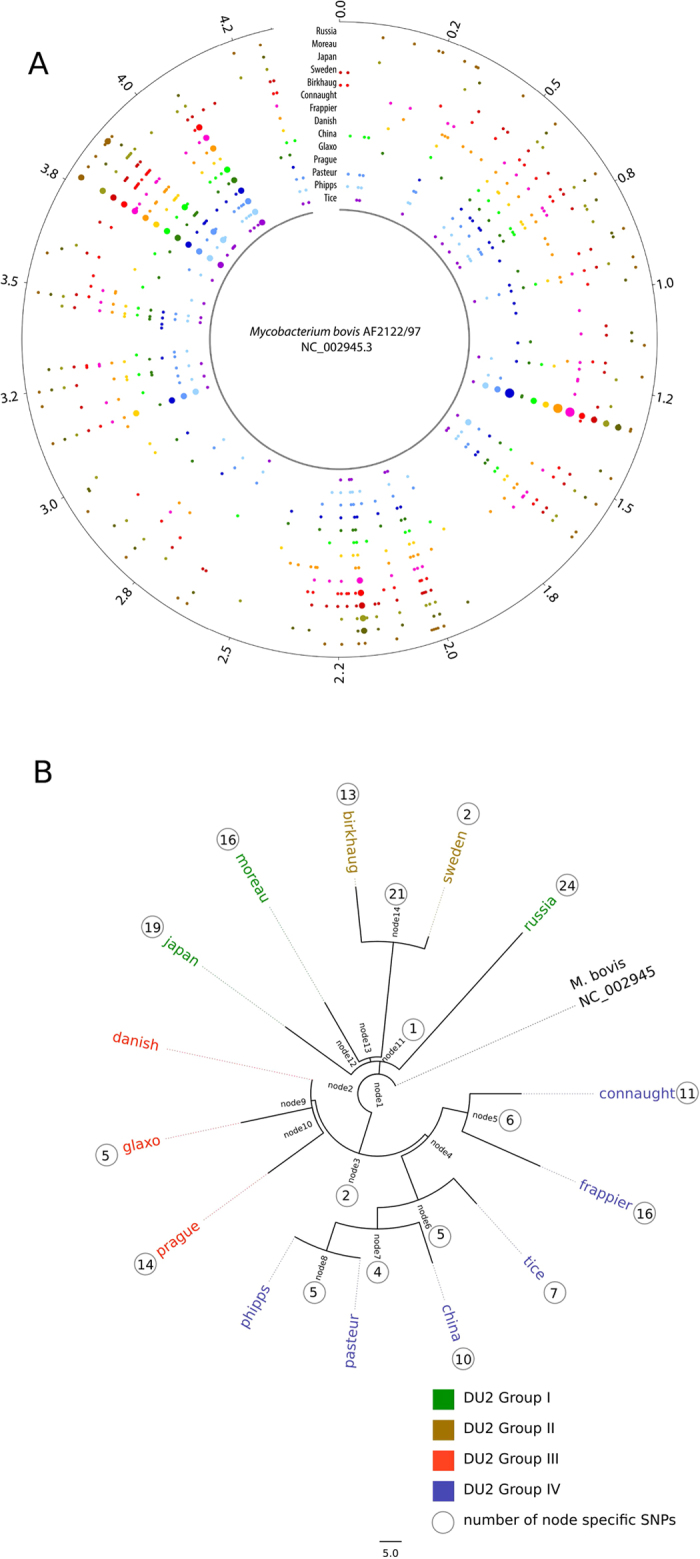
(**A**) A circular representation of Bacillus Calmette-Guérin (BCG) vaccine sub-strains chromosomes. Distribution of single nucleotide polymorphisms (SNPs) in fourteen BCG strains compared to *Mycobacterium bovis* AF2122/97. Each coloured blob corresponds to the SNP density in a non-overlapping window of 10000 nucleotides, with a unique colour per sample allowing easy visual assessment of similarities between samples. The scale is shown in megabases in the circle. (**B**) Phylogenetic relationships among BCG strains. Maximum likelihood phylogeny tree based on 700 + variable common nucleotide positions across fourteen BCG genome sequences. The tree was rooted using *M. bovis* AF2122/97.

We sequenced fourteen BCG genomes and one *M. bovis* isolate to a mean depth of coverage of 250-fold (range 152 to 413) to produce a catalogue of differences between the strains. High quality SNPs (as compared to the *M. bovis* AF2122/97 reference^34^) were identified (Fig. 1A) with numbers ranging from nine SNPs and 12 small indels (up to 10 bp) in the re-sequenced *M. bovis* to 789 SNPs and 94 small indels in BCG Moreau (Table S1, S2). All strains, except BCG Phipps, contained variants that are unique to that individual strain (Table S1).

A maximum-likelihood phylogeny using SNPs has a similar structure to that seen in previous studies that were based on tandem duplications and deletions (Fig. 1B). In particular the members of the DU2 groups, originally described by Brosch *et al.*,^18^, continue to cluster together (Fig. 1B). However, the phylogenetic position of BCG Sweden and BCG Birkhaug differs slightly relative to the DU2-based classification, where these strains share a common deletion named Δint, internal of the duplicated region DU2 with strains from DU groups III and IV^18^.

**Figure 2:**
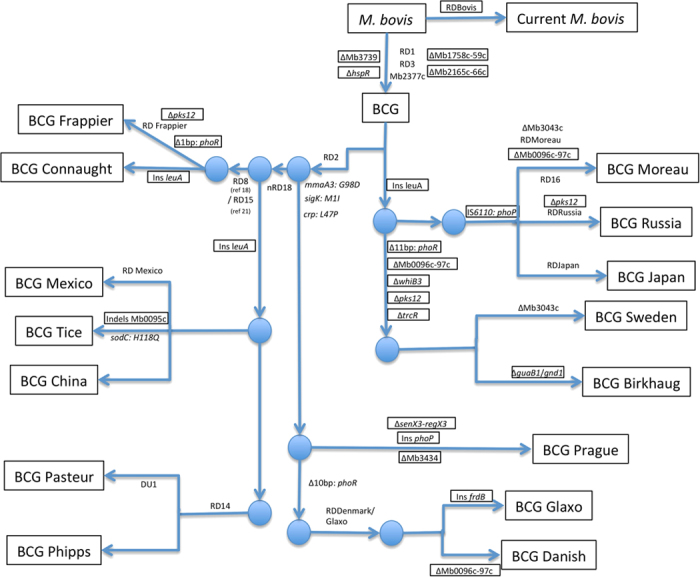
Revised genealogy of Bacillus Calmete-Guérin (BCG) vaccine strains. The genealogy of BCG vaccine strains, displays the original virulent ancestor strain *M. bovis* and the subsequent series of genomic alteration including deletions of regions of difference (RD), and some strain-specific insertion (‘Ins’) and deletions (‘Δ’). Genes with both insertions and deletions is also shown (‘Indels’). Genetic markers identified in this work have been added to the scheme (squares bordered with a red solid line). Note that the RD nomenclature in previous work differs between references^16^,^18^ deletion of RD8^16^ from BCG strains Connaught and Frappier corresponds to RD15 in reference^18^. Variants shown in boxes are novel in this study. All variants shown were manually inspected by read mapping as well as by assembled genomes.

By applying a large indel detection (>20 base pair) pipeline that has been described earlier^35^, we identified a number of previously unknown deletions and insertions in our analysis (Fig. 2, Table S3). An interesting finding is a 103 bp deletion was present across all BCG strains, and eliminates the distal end of *hspR*. This gene is involved in transcriptional regulation (repression) of heat shock proteins and known to impact virulence. The *hspR* locus activates a subset of the heat-shock general stress response upon macrophage invasion^36^, and is necessary in the persistent phase since *hspR* deletion (Δ*hspR*) strains exhibit attenuated growth in chronic infection^37^. An analysis of depth of coverage revealed a duplication of 2,900 bp segment that spans the region between 1,276,501–1,279,400 base pairs (*M. bovis* coordinates). This duplicated fragment is present in all BCG strains (Fig. S2A).

The key studies on BCG plasticity relied upon DNA arrays, RD screening and Southern blotting of restriction digests together with hybridization to estimate genealogies^16^,^18^. More recently Garcia Pelayo *et al.*, used a minimal set of SNPs to follow the geneology of BCG strains^20^, followed by Zhang *et al.* and Copin *et al.*, who produced a similar genealogy for BCG vaccine strains, using whole-genome sequencing data and *de novo* assembly^38^,^39^. In order to fully interrogate our transcriptome data we produced *de novo* genome assemblies for all fourteen sequenced BCG vaccine strains together with *M. bovis*. The overall genealogy structure is similar to the earlier study^38^, but with some notable differences (Fig. 2, Table S4).

BCG Connaught was added to the genealogy as part of DU2 Group IV and is very similar to BCG Frappier. A 1 bp deletion in *phoR* and the characteristic deletion of the region *Mb3525c* to *Mb3527c* (RD Frappier) can be used to identify BCG Frappier. In contrast to other BCG strains and *M. bovis*, BCG Frappier did not seem to show the same partial duplication of gene *leuA* compared to *M. tuberculosis* H37Rv or *M. canettii* CIPT 140010059^40^. The latter gene appears to be a common site for sequence duplications amongst *M. bovis* and BCG strains, for example, with a 202 bp longer *leuA* gene in *M. bovis* AF2122/97. The lengths and positions of these duplications vary among BCG strains with only BCG Frappier containing a *leuA* with the same structure as that present in H37Rv and *M. canettii* (Table S5). Elevated coverage depth signaled two duplications in the genome of BCG Frappier. These duplications comprise 600 bp that encompasses genes *Mb0816c-Mb0817* (Fig. S2B) and 1,650 bp that encodes the cytochrome oxidase assembly factor *ctaB* (Fig. S2C).

BCG Tice belongs to the same group as BCG Mexico but can be differentiated by the three genomic regions described previously^41^. Interestingly, in our study BCG China fits into this group. The only previously sequenced version of this strain contained RD2 as the only major region of difference^38^. However, considerable variation appears to exist between the previously submitted genome of BCG China and the strain that was used in our study. Our re-sequencing of BCG China strain demonstrates that it also contains the nRD18 deletion, thereby homologous with BCGs Tice and Mexico. To confirm our finding we mapped the draft genome assembly produced by Zhang *et al.*,^38^ to our assembly, which demonstrated that the nRD18 region of difference is also present in their original data (Fig. S1). Thus it would appear that there are more than one strains named as BCG China in circulation, which clearly belong to different branches of the BCG genealogy. While the BCG China strain characterized by Garcia Pelayo *et al.*,^20^ based exclusively on SNPs, clusters with the BCG Danish group of strains (DU2 group III), the BCG China strain used in our study is close to the BCG Pasteur strain (DU2 group IV).

As in previous studies BCGs Pasteur and Phipps share considerable homology, with RD14 thought to be the primary distinguisher^18^,^38^. However, we have shown that both BCG Pasteur and some strains of BCG Phipps share this region of difference (Fig. 2, Table S4). It is possible that the original (mother) culture of BCG in 1937–1938 actually had a mixed population for RD14, with some bacteria containing this region and others having it deleted. The BCG Phipps derived from this mother culture in this study appears to have RD14 deleted. Therefore the only key difference that we can be certain of between these two strains, which diverged relatively late in the history of the BCG vaccine, is BCG Pasteur’s DU1 duplication.

The other BCG strains that also constitute the “late” group fall within DU2 Group III. BCG Mérieux was unavailable for sequencing but we could confirm that BCG Prague, Glaxo and Danish all belong to this group (Fig. 2). BCG Prague continues to be the outlier as it does not contain any deletions in *phoR* but does have a 1 bp insertion in *phoP*, ensuring this gene’s pseudogenisation due to a frameshift mutation (Fig. 2). BCG Glaxo and Danish both share a region of difference in *Mb1840–Mb1841*, with the deletion of a Mg^2+^ transport ATPase and NADH dehydrogenerase, with relatively minor changes differentiating between them: an insertion of 5 bp after *Mb2088c* (cobalamin biosynthesis protein) in BCG Glaxo and a deletion of *Mb0097c* and *Mb0098c* in BCG Danish (a common site with variable length deletions found in this region in BCG Danish, Sweden, Birkhaug and Moreau).

Finally, BCGs Russia, Moreau, Japan, Sweden and Birkhaug make up the “early” group of strains, identified by their intact RD2 region. BCGs Sweden and Birkhaug contain DU2 Group II with a significant number of shared gene deletions including *phoR*, *Mb0097c* and *Mb0098c*, *pks12*, *trcR* and *whiB3* (Fig. 2). The DU2 Group I strains contain an *IS*6110 upstream of *phoP* that *M. bovis* and all other BCG strains have lost. Furthermore, our analysis confirmed the DU2-I duplication in BCGs Japan, Moreau and Russia demonstrated earlier^18^. A depth of coverage analysis suggests that DU2-I comprises around 20 kb of duplication in the region of 3,638,751–3,659,450 base pairs (*M. bovis* AF2122/97 coordinates). Elevated coverage depth was also observed for the direct repeat (DR) region (CRISPR-Cas locus) of these strains corresponding to region 3,076,201–3,080,250 base pairs in *M. bovis* AF2122/97, which encodes an *IS6110* insertion sequence (*Mb2838c-Mb2839c*) and many small direct repeats (Fig. S2D).

### Drug resistance phenotype to anti-tuberculosis drugs

It has been observed for some time that differences exist in the susceptibility of different BCG strains to anti-tuberculosis drugs. As they are derived from *M. bovis* they are all pyrazinamide-resistant due to the H57D mutation in *pncA*^42^. In addition, they are also resistant to cycloserine, partly due to the G122S mutation in *cycA*^43^. A previous study found that the “early” strains typically have an MIC for isoniazid (INH) of 0.05 μg/mL, compared to 0.1–0.2 μg/mL for the “late” strains^44^. This modestly elevated MIC has existed in all strains obtained from the Pasteur Institute since 1926.

We repeated this drug susceptibility testing on the BCG strains and found a similar pattern (Table 1). Many of the post-1926 strains (BCGs Prague, Glaxo, Tice, Connaught, Phipps and Pasteur) examined had raised MIC values, often 1 μg/mL or greater. BCG China could also be added to this group with an INH MIC of 1 μg/mL. Previously Behr *et al.*,^45^ have observed that strains obtained from the Pasteur Institute prior to 1927 produced methoxymycolates *in vitro* but those obtained later could not synthesize methoxymycolates. This phenotype has been attributed to a point mutation at position 293 in *mma3*^45^. We found the same SNP in all of the strains with a raised INH MIC, including BCG China (Fig. 2), and therefore we postulate that this SNP can lead to low-level INH resistance This is not surprising, as INH is known to inhibit the synthesis of α-mycolate, methoxymycolate and ß-mycolate^46^. The appearance of this mutation in BCG China as well as its correlation with phenotypic resistance lends further support to it being the mutation responsible for INH resistance.

### Transcriptome variation

Genomic differences constitute one possible reason for the observed differential phenotypic properties of BCG strains, However, this gives only a partial picture as regulatory events occurring on the transcriptional and translational level are largely ignored. A prolonged period of culturing under nutrient-rich conditions is bound to have strong selective effects on gene regulation. In order to reveal the differences in expression profiles of BCG strains, we performed a global transcriptome analysis across all fourteen BCG vaccine strains grown *in vitro* focusing on the changes in the gene expression profile during exponential growth of the bacilli. RNA extracted from three independent exponential phase cultures of *M. bovis* and daughter BCG strains were used to generate cDNA preparations that were then analyzed by Illumina-based RNA-sequencing. Data quality was assessed using Pearson correlation coefficients (Table S6) and principal component analysis (PCA) (Fig. S3), which demonstrated the degree of reproducibility between biological replicates. The total transcriptome data are recorded as an average of the clustered samples. A global view of relative expressed profiles of differentially expressed genes showed extensive variation in gene expression, both between early and late BCG daughter strains and with respect to virulent tubercle bacilli (Fig. S4). This supports the earlier genomic observation that the earlier strains are most closely related and cluster together. Quantitative RT-PCR analysis of 15 randomly selected target genes was conducted to confirm the validity of the data generated through global transcriptional profiles. The qRT-PCR results displayed similar trends of up- or down-regulation to those observed in RNA-seq results, supporting the validity and robustness of the RNA-seq data (Fig. S5).

To understand potential functional changes arising from differentially expressed genes, enrichment analysis of gene ontology (GO) terms across three GO categories—biological processes (BP), molecular functions (MF) and cellular components (CC) were investigated (Fig. S6). The overrepresented categories in the subset of up-regulated genes include: 1) translation (BP); 2) structural constituent of ribosome (MF) and 3) ribosome (CC). While in the down-regulated subset these categories comprised: 1) regulation of transcription, DNA-dependent (BP); 2) sequence-specific DNA binding transcription factor activity (MF) and 3) integral to membrane (CC). We observed arginine biosynthetic process pathway associated genes were expressed nearly exclusively in BCG Glaxo (Fig. S6).

### Metabolic remodelling and adaptations

**Figure 3:**
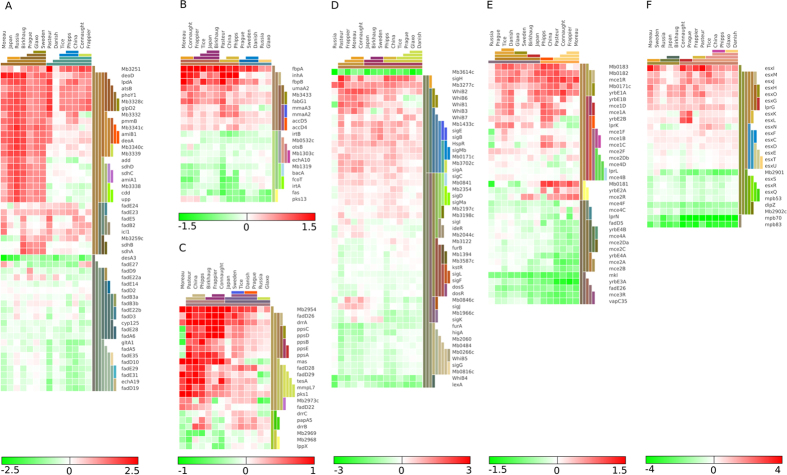
Transcriptional profile of a global collection of BCG strains: Gene expression heat maps of genes showing expression profiles involved in; (**A**) Catabolism of fatty acids. (**B**) phenolpthiocerol dimycocerosate (PDIM). (**C**) mycolic acid synthesis. (**D**) Transcriptional regulatory. (**E**) Mammalian cell entry genes. (**F**) Immunogenic surface and secreted proteins. Genes were selected based on their annotation. The color scales represent log2-fold changes in gene expression (DESeq), using the *M. bovis* AF2122/97 strain as reference.

During *in vitro* growth, *M. bovis* is unable to use glycerol as a sole carbon source and subsequently unable to use carbohydrates to generate energy due to the lack of a functioning pyruvate kinase^47^, which blocks the ATP-generating roles of glycolysis and the pentose phosphate pathway. In line with previous results^18^, our data showed higher levels of transcription of *glpD2* across all BCG strains compared with *M. bovis* linked to the DU2 duplication (Fig. 3A) and strong up-regulation of energy production related genes including NDH-1 and the ATP synthase operons (Fig. S7). These results reflect the ability of BCG strains to use glycerol as a carbon source and carbohydrates to generate energy. By contrast, *frdA*, *aldA, aldB*, and *nuoJ*, encoding fumarate, aldehyde and l-alanine dehydrogenase and NADH dehydrogenease I respectively, were suppressed differentially among BCG strains (Fig. S7). Additional confirmation of metabolic remodelling is seen in the divergent regulation of genes associated with fatty acid degradation (Fig. 3A). Hence, genes that collectively are predicted to encode enzymes necessary for the biochemical activation and β-oxidation of fatty acids, including fatty acid-coenzyme A (CoA) synthase, acyl-CoA dehydrogenase, enoyl-CoA hydratase and acetyl-CoA transferase were all divergently regulated among BCG strains. Similarly, a lipid desaturase gene *desA3* that is putatively involved in fatty acid modification^48^, was suppressed across all BCG strains compared with *M. bovis* (Fig. 3A). A previous study showed that inactivation of the *desA3* gene attenuates *M. tuberculosis* in mice^49^, suggesting a key role for this lipid desaturase gene in virulence. Taken together, these data support the view that BCG uses glycerol as a carbon source during *in vitro* growth^47^, and provides clues to an alternative mechanism by which BCG is attenuated.

Lastly, remodelling of the cell surface and lipid metabolism is indicated by the divergent regulation of the genes involved in phthiocerol dimycocerosate (PDIM) and mycolic acid synthesis (Fig. 3B,C). Whereas mycolic acid synthesis appeared suppressed in BCG compared to *M. bovis*, we noted marked induction of the synthesis of mycolic acids consistent with the induction of *umaA2*, *accD4*, *accD5*, *mmaA2*, *inhA*, *fbpA*, *fbpB*, *fabG1* and *Mb3433* (Fig. 3B). Altogether, our results indicate that BCG strains vary in their cell wall composition compared to *M. bovis*, providing another explanation for both altered immunogenicity and variable drug resistance.

### Adaptations in regulatory and virulence genes

In line with the metabolic remodelling, the expression of several transcriptional regulators was divergent between *M. bovis* and BCG strains (Fig. 3D). Several transcription factors are up-regulated in BCG such as *hspR*, *Mb0361, Mb0841, Mb1433c,* and *Mb3702c*. Similarly, the RNA polymerase sigma factor *sigA* was up-regulated in all strains except BCG Russia (Fig. 3D). This sigma factor has previously been recognised as differentially expressed in virulent versus avirulent strains of *M. tuberculosis*^50^. Other up-regulated RNA polymerase sigma factors are *sigB, sigE, sigH, sigM*. Differential expression is seen among the *whiB* family of transcription factors, which regulate virulence, stress responses and cell division^51^,^52^,^53^. *whiB1*, *whiB2* and *whiB3* were overexpressed, while *whiB4* was down-regulated in BCG strains compared to *M. bovis*. Interestingly, data revealed that differential expression of transcriptional activators showed the least variation in BCG Russia (Fig. 3D).

Analyses of genes encoding virulence and immunogenic surface and secreted proteins also revealed variable levels of gene expression profiles across BCG strains. Several virulence factors required for cell invasion or escape such as *mkl* (*mceG*), thought to be essential for the function of the *mce* loci involved in nutrient uptake, are suppressed in all BCG strains except BCG Tice (Fig. 3E). Strikingly, our results show that *mce2A, mce2B, mce2C, mce2D, mce4A, yrbE4A* and *yrbE4B* are highly suppressed in BCG Pasteur, Moreau, Connaught and Frappier whereas *mce1R* showed increased expression across all BCG except Russia (Fig. 3E). *mce1R* is a transcriptional regulator of mce1 operon^54^ and this overexpression suggests increased negative regulation of *mce1* operon and therefore may be relevant to attenuation.

In line with an earlier report^55^, expression of the antigenic region that encodes the major secreted immunogenic proteins Mpb70 and Mpb83 were significantly suppressed in late BCG strains as well as the immunogenic protein Mpb64, which have been deleted from the later strains (RD2) and suppressed in the earlier strains (Fig. 3F). Furthermore, several immunogenic factors such as ESAT-6 like proteins (*esxC, esxD, esxG, esxH, esxK, esxI, esxJ, esxL, and esxM*) as well as *lprG* are up-regulated in all BCG strains, suggesting that this could be a compensation for ESAT-6 deletion. Interestingly, three ESAT-6-like genes (*esxQ, esxR*, and *esxS*) showed reduced expression compared with *M. bovis* (Fig. 3F). Importantly, it is worth noting the variable expression of PE and PPE families, including PE_PGRS proteins, (Fig. S8) thought to be responsible for the antigenic diversity^56^. Interestingly, we observed over expression of ESX-5 locus genes including *esxN*, *pe18, ppe25, ppe26 and ppe27* exclusively in BCG Tice as a result of ESX-5 locus duplication in BCG Tice^57^ (Fig. 3, Fig. S8). Duplication of the ESX-5 locus could be selected for by certain growth conditions, as recent research has indicated the ESX-5 is involved in outer membrane permeability and nutrient import^58^. Finally, T cells are potent effectors of acquired immunity against intracellular bacterial pathogens including *Mycobacterium* species. It has been observed previously that most BCG T-cell epitopes are highly conserved at sequence level^38^, however, our analysis demonstrated a highly diverse T cell antigen expression profile when compared to *M. bovis* (Fig. S9).

### Pairwise comparison of high and low immunogenicity strains

The most thorough randomised clinical trial of different BCG vaccines to date compared the immune responses of newborn infants to BCG Danish, Japan and Russia^27^. The proportion of polyfunctional CD4^+^ T cells was significantly higher in the infants immunised with BCG Danish or Japan compared to Russia. Infants immunised with BCG Japan also had higher concentrations of secreted Th1 cytokines and infants immunised with BCG Danish had higher proportions of CD107 expressing cytotoxic CD4^+^ T cells. This suggests that BCG Japan and BCG Danish could be more immunogenic than BCG Russia. This finding led us to particularly explore the transcriptional differences among these strains.

**Figure 4:**
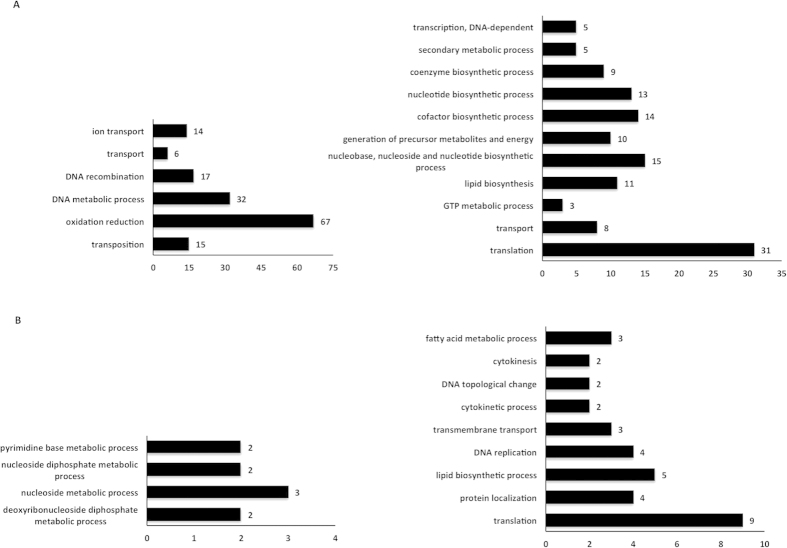
Pattern of increased and decreased expression in genes of BCG Japan and Danish compared to our own assembled genome of BCG Russia. (**A**) Genes that are significantly down-regulated or up-regulated in BCG Japan compared to BCG Russia. Genes are categorized based on significant enrichment in biological processes of gene ontologies (p < 0.05). Left: total number of genes found to be down-regulated in each biological process category. Right: total number of genes found to be up-regulated in each biological process category. (**B**) Genes that are significantly down-regulated or up-regulated in BCG Danish compared to BCG Russia. Genes are categorized based on significant enrichment in biological processes of gene ontologies. Left: total number of genes found to be down-regulated in each biological process category. Right: total number of genes found to be up-regulated in each biological process category.

Both BCG Japan and BCG Danish had a number of genes that showed significantly greater expression than the equivalent genes in BCG Russia. For BCG Japan 348 genes were up-regulated and 541 genes were down-regulated compared to BCG Russia. Our analysis showed an enrichment of genes involved in transport, DNA-mediated transposition, DNA metabolism and oxidation-reduction processes in the list of down-regulated genes (Fig. 4A). Of these, oxidation-reduction was the largest group containing 67 genes, followed by DNA metabolic processes containing 32 genes. For genes up-regulated in BCG Japan compared to Russia, these predominantly consisted of genes involved in translation including 11 ribosomal proteins from the 30 S subunit and 13 proteins from the 50 S subunit (Fig. 4A).

In the comparison between BCG Danish and BCG Russia, 107 genes were up-regulated and 39 were down-regulated. No clear enrichment was evident for the genes down-regulated in BCG Danish with sharing a nucleoside metabolic process (Fig. 4B). However for the up-regulated genes an enrichment of genes involved in translation was again seen with two being 30 S subunit ribosomal proteins and five being 50 S proteins (Fig. 4B).

### Comparison of BCG proteomes

BCG strains are classified into four groups (I-IV) based on RD and DU2 markers^18^. However, our phylogeny produced on genome-wide SNP-differences (SNP-barcodes) re-classified BCG isolates into five groups (Fig. 1B). Coupled with our understanding of the global transcriptional profile, we performed quantitative protein analysis on five representatives of these groups. Using a mass spectrometric approach with tandem mass tagging for quantification (TMT)-labeling, we identified a total of 794 proteins and determined their differential expression (detailed in Table S7). For a global overview of key changes in metabolism, proteins displaying high differential regulation (2-fold change differences) were grouped into functional categories based on the Bovilist/TubercuList server classification^59^. The highest level of diversity in expressed proteins related to information pathways and intermediate metabolism and respiration (Fig. S10), again reflecting the metabolic remodeling as discussed earlier. Differential regulation was also observed for proteins involved in cell wall and cell processes and lipid metabolism (Fig. S10).

**Figure 5:**
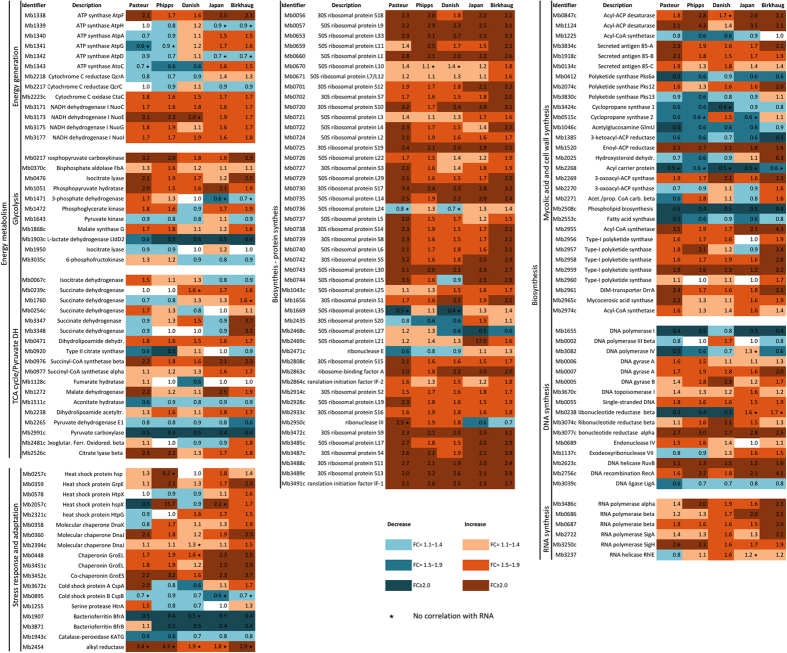
Heat map of differentially expressed proteins among five representative BCG strains compared to *M. bovis.* Differential expression of proteins involved in biochemical pathways related to energy metabolism (**A**), or to biosynthesis pathways (**B**,**C**) are shown. The color scale indicates differential regulation as the Fold Change of the protein amount relative to the *M. bovis* 2122/97 reference strain. Up-regulation is indicated in red, down-regulation in blue. Star (*) indicates lack of correlation with RNA-seq data for that gene.

On the basis of these findings, we subsequently focused on metabolic pathways connected to synthesis or energy production. Proteins mapped to these metabolic pathways were selected and their differential regulation was presented in a heat map (Fig. 5). We regarded proteins showing an increase or decrease with a fold change ≥2.0 as strongly expressed, those with fold change ≥1.5 but <2.0 as moderately regulated and proteins with fold change <1.5 as not regulated. Components of the ATP synthase complex, NADH dehydrogenase and ubiquinol-cytochrome C complex were divergently induced (Fig. 5A). Moreover, enzymes of the glycolytic pathway, components of the tricarboxylic acid cycle and the pyruvate dehydrogenase complex, an enzyme linking glycolysis with the tricarboxylic acid cycle, were displaying differential regulation (Fig. 5A). Among the expressed proteins, some have been previously described as virulence proteins in different strains of *M. tuberculosis* complex including heat shock protein HspX, the secreted antigen 85-A and C, GroELs, phosphoenolpyruvate carboxykinase (*pckA*), isocitrate lyase (*icl*), 3-oxoacyl synthase II (*kasA*) and alkyl-hydroperoxide reductase (*ahpC*)^60^,^61^,^62^ (Fig. 5). Induction was also observed for various components of the stress response, including heat shock proteins, co-chaperonins proteins and bacterioferritins (Fig. 5A). Induction of these facilitators of protein re-folding may contribute towards optimizing the integrity of synthesized proteins or to recycling of existing proteins.

Furthermore, major biosynthetic pathways were generally found divergently regulated among BCG strains, including ribosomal protein synthesis, DNA biosynthesis and synthesis of mycolic acids, a key component of the mycobacterial cell envelope^63^ (Fig. 5B,C). Variation in expression was observed for several polyketide synthases, several ribosomal subunits and for DNA helicase and a ribonucleotide-diphosphate reductase subunit, which are components of lipid, protein and DNA biosynthesis, respectively (Fig. 5C). Taken together, the observed changes in the BCG strains proteome support the results obtained from the transcriptome study and reflect how *in vitro* growth adaptation causes phenotypic differences amongst the BCGs.

## Discussion

Although BCG is the only licensed vaccine against tuberculosis, its history has been clouded by variable efficacy and reports of strain variability in morphology, growth rate, gene/protein expression and genetic make-up^64^. Several reasons have been suggested to explain the varying protective efficacies of BCG, including the genetic differences between vaccines strains as well within a given sub-strain^17^,^18^. In this study, we have surveyed the heterogeneity among BCG strains through a combination of genome sequencing, comparative transcriptome and proteome analyses. Our combined approach has enabled us to identify signatures of genetic and transcriptomic variability across fourteen BCG strains, which may play critical role in observable differences in vaccine efficacy between them.

One signature, consistent with the previous study^18^, is that the SNP phylogeny is congruent with the genealogy constructed based on large insertions and deletions. For BCG China, our result and the previous finding were discrepant. An earlier report claimed that BCG China is a successor of BCG Danish and displays the DU2-III duplication^57^. However, our analysis places BCG China into the group of DU-IV strains, which asks for a closer inspection of the source of the previously sequenced BCG China strain in comparison with the BCG China strain used in our study. It also raises a concern that multiple circulating strains of “BCG China” may be present and these may lead to the production of vaccines with varying immunogenicity with the same name-tag.

Genomic profiles of BCG Sweden and BCG Birkhaug are similar and they shared similar deletions^57^. Our study revealed a novel deletion locus shared by these two strains together with Russia and Frappier, which distinguishes them from other BCG family members (Fig. 2). The variably sized deletions concerned the *pks12* gene, which encodes polyketide synthase 12, known to be necessary for β-phosphomycoketide (MPM) production and the CD1c-mediated T-cell response^65^. Deletion of *pks12* genes in each of *M. tuberculosis* strains H37Rv, CDC1551, and BCG Pasteur strains completely abolished the ability of all three strains to activate CD1c-restricted T-cells and to produce mannosyl-β-1-phosphoisoprenoids (MPI)^65^. It is therefore possible that deletions within *pks12* may directly affect the immunogenicity of the vaccine as CD8-1-reactive MPM cannot be detected in non-pathogenic mycobacteria such as *M. smegmatis*, *M. phlei* and *M. fallax*^65^. The *pks12* gene has not been found in any non-pathogenic mycobacteria, suggesting a possible direct effect on virulence. Deletions of *pks12* have also been shown to increase drug resistance in *Mycobacterium avium* through a cell wall–remodeling pathway, although the impact of this on BCG strains is currently unknown^66^.

Apart from the use of BCG strains in vaccinology, BCG strains have also been used in immunotherapeutic applications in bladder cancer treatment. However, higher bacillic doses are required for bladder cancer immunotherapy (10^8^–10^9^ bacilli) than for either oral BCG vaccination (10^7^ bacilli) or intradermal vaccination (10^5^ bacilli). This may explain the higher likelihood of disseminated infection in immune-compromised individuals undergoing this treatment. Furthermore, variations in efficiency of immunotherapy has been observed between BCG Connaught and BCG Tice in bladder cancer patients^33^, which might be linked to a point mutation in the *sodC* dismutase encoding gene of BCG Tice that we also have confirmed.

Moreover, BCG strains may also differ in their antibiotic resistance levels. A previous study demonstrated that all BCG strains obtained from Pasteur Institute after 1926 had moderately elevated levels of resistance to INH^44^ and this phenotype coincides with a point mutation in the *mma3* gene, resulting in impaired methoxy mycolic acid production^45^. Our analysis of *mma3* genes in BCG strains confirms the same mutation in all late BCG strains, including BCG China, and we believe that this mutation is causative for the moderately elevated levels of resistance to INH. Our result establishes that there are significant differences in the sensitivity to INH between the earlier and later BCG isolates, a finding that has important implications for BCG immunotherapy.

Analysis of the transcriptome of the strains shows remarkable variation in gene expression, both between early and late strains and within groups of BCG strains. In particular, changes in the expression of certain cell envelope components suggest that BCG daughter strains have adapted to their environments by the divergent regulation of fatty acid-degrading enzymes and DNA repair proteins, and the remodelling of their envelopes. Many of the cell wall components of *M. tuberculosis* have been associated with virulence and modulation of the host immune response^67^. Although the function of lipoproteins in mycobacteria have not been well characterized, some lipoproteins such as phospholipase C have been reported to modulate the host immune response and may play an important role in virulence^68^. Similar functions have also been attributed to some exported proteins^69^. We hypothesize that this regulation of a series of genes involved in the synthesis of cell wall and cell membrane components may impact upon BCG attenuation and the vaccine’s variable efficacy.

The up-regulation of genes involved in translation in both BCG Danish and BCG Japan when compared to BCG Russia suggests an increase in expression of ribosomal proteins. It is not surprising that the induction and expression of ribosomal proteins increases the immunogenicity of a vaccine, first observed when guinea pigs immunized with a ribosome-rich fraction of the avirulent *Mycobacterium tuberculosis* H37Ra were protected against subsequent infection by the virulent H37Rv^70^. Subsequent work has shown that the 50 S subunit provokes a strong delayed-type hypersensitivity reaction in sensitized guinea pigs^71^ and that whole ribosomes are required for this reaction, not just the protein or RNA components^72^. Similarly studies on blood samples from BCG-vaccinated healthy volunteers showed increased cell proliferation and interferon-gamma responses related to the immune-dominance of detergent phase fractions of *M. tuberculosis* that were ribosomal protein-rich^73^, many of which are membrane-associated^74^. This increase in ribosome production in carbon-limited BCG cells is subject to growth rate-dependent control^75^. Therefore we suggest that the propensity of BCG Danish and BCG Japan to increase their ribosome number through increased gene expression may directly lead to their increased immunogenicity, emphasizing the importance of ribosome abundance in effective tuberculosis vaccines.

The proteome analysis with relative quantification labeling of the selected BCG strains reveals a profile consistent with their transcription, with proteins functionally involved in biosynthesis, energy metabolism and virulence, detoxification or adaptation significantly over-represented in BCG strains as compared to *M. bovis*. Components of stress response heat shock proteins strongly up-regulated in BCG Phipps while co-chaperonin GroEL and GroES, were strongly up-regulated in BCG Japan and Birkhaug. GroEL1 and 2 are chaperonins participate in folding of newly synthesized, imported, unfolded or poorly folded proteins as well as being involved in the heat-shock response. GroEL1 (*Mb3451c*) is a potent inducer of cytokine synthesis, including interleukins (IL) IL-1β, IL-6, IL-8, IL-12, tumor necrosis factor-α (TNF-α) and granulocyte-macrophage colony-stimulating factor (GM-CSF)^76^, while GroEL2 (*MB0448*) is shown to be a key element for mycobacterial-survival inside macrophages and is well recognized by the immune system, *in vitro* and *in vivo*^77^. Overexpression of these proteins including *AhpC* could be a survival mechanism for these strains, prolonging their intracellular stay in macrophages and allowing for more efficacious antigen processing and presentation, concomitantly with increased protection as result of prolonged immune system activation. On the other hand, since these proteins are a key element for virulence, there appears to be a positive correlation between virulence level and protective efficacy of vaccine strains. A similar correlation was observed in early animal studies^78^,^79^. Furthermore, the cumulative effects of variation in secreted and cell wall antigens across BCG strains may contribute to variable vaccine efficacy. Taken all together, these findings add to the growing evidence that BCG vaccine strains differ too widely to be considered a single vaccine for comparative and regulatory purposes. We propose that a single vaccine strain should be chosen and used for global vaccination purposes to prevent exposing populations to a less immunogenic or more drug resistant strain of BCG. Future systematic studies on comparative efficacies of all of these BCG vaccine strains in various host backgrounds will help to determine the most effective vaccine strain that may be recommended under a given set of environment including host immune status. Our results provide a comprehensive framework to understand the underlying genomic variability and the gene and protein expression readouts for such variability, resulting from nearly 100 years of *in-vitro* evolution since the vaccine was first developed.

## Methods

### Bacterial strains and growth conditions and DNA isolation

*Mycobacterium bovis* BCG substrains were collected from Marcel Behr, McGill University, BCG China from Baoli Zhu, Chinese Academy of Sciences and *Mycobacterium bovis* from Roland Borsch, Institute Pasteur. Bacteria were grown at 37 °C on Middlebrook 7H10 agar plates supplemented with 10% OADC (oleic acid–albumin–dextrose–catalase, BD Biosciences) or in shaking cultures in Middlebrook 7H9 liquid medium supplemented with 10% ADC (albumin–dextrose–catalase, BD Biosciences) and 0.05% Tween 80. Genomic DNA was prepared using the bead beater–phenol/chloroform extraction method.

### Genome Sequencing and Assembly

Paired-end genomic libraries were prepared using TruSeq DNA Sample Preparation Kits V2 (Illumina Inc, San Diego, CA, USA) and sequenced on an Illumina HiSeq 2000. Raw reads were trimmed with Trimmomatic^80^ to an average quality score of 20 within a window size of 4 bp. Initial contig assembly was performed with Velvet^81^, using VelvetOptimiser to choose kmer values ranging from 33 to 81 [http://www.vicbioinformatics.com/software.velvetoptimiser.shtml]. Contigs were orientated and ordered with *M. bovis* AF2122/97 (EMBL accession number BX248333.1) as the reference genome and scaffolding and iterative gap filling were performed using PAGIT toolkit^82^.

### Genome alignment and phylogenetics

The raw paired-end reads were also mapped against the reference *M. bovis* genome using BWA^83^. SNPs were identified using Samtools^84^ and filtered to ensure that they had a quality score more than 30 and were present in at least 75% of the mapped reads in each direction. The reference genome was segmented into 5000 bp non-overlapping sliding windows per sample. The SNP density in each of these windows was represented by a colored circle in Fig. 1A, with different colours representing different samples. This Figure was composed using the Circos visualization package [http://circos.ca]. Filtered SNPs were also concatenated and used as alignments for the estimation of a maximum likelihood phylogeny using the default settings of RAxML v0.7.4. 100 initial trees were generated and the best tree identified, followed by the production of 100 bootstraps.

### Detection of indels

Large deletions were detected as described^35^. Briefly, large deletions (>100 bp) were determined using a combination of tools based on paired-end, split-read and depth of coverage approaches. Reads at putative deletions (300 bp) predicted by all five tools were extracted from bam files and subsequently *de novo* assembled using Velvet^81^. If a derived contig happened to be split into two parts when mapping it back to the reference with high similarity (>95%), the contig was considered a cross-junction contig (CJC)^85^. Deletions without at least one CJC were considered to be false positives and were therefore discarded. Deletions in PE/PPE genes were filtered out due to the complexity of such regions. All validated deletions were gathered and merged when having a mutual overlap greater than 90%. Insertions were detected by performing *de novo* assembly and aligning resulting contigs to the reference genome in order to find novel sequences present in the target genome and absent in the reference.

### RNA isolation and purification

Forty milliliters of exponential growth phase culture was harvested at an OD_600_ of 0.6. Bacteria were re-suspended in 1 ml of TRIzol (Invitrogen) and added to a 2-ml screw-cap tube containing 0.5 ml zirconia beads (BioSpec Products). Bacterial cells were disrupted by bead-beating 5x for 30 seconds with a 1-minute interval on ice. After centrifugation of lysed mycobacteria, supernatants were extracted with chloroform and RNA was precipitated with sodium acetate (2 M, pH 5.2) and isopropanol. RNA pellets were washed with 75% ethanol and resuspended in RNase-free water. Contaminating DNA was removed with RNase-free DNase (Fermentas) prior to RNA purification through an RNeasy MinElute Cleanup kit (Qiagen). The quality of RNA was assessed using a Nanodrop (ND-1000, Labtech) and the Agilent 2100 Bioanalyzer (Agilent; Palo Alto, CA, USA) as per manufacturer’s recommendations.

### Enrichment of mRNA from total RNA and Library preparation for RNA-seq analysis

As ribosomal RNA comprises the vast majority of the extracted RNA population, depletion of these molecules through RiboMinus-based rRNA depletion, has been used in efforts to increase the coverage of mRNA and to reduce rRNA reads. For this mRNA enrichment, the Invitrogen’s RiboMinus^TM^ Prokaryotic kit was used according to manufacturer’s instructions.

Construction of double-stranded cDNA libraries preparation was carried out essentially using Illumina TruSeq^TM^ RNA sample preparation kit V2 according to manufacturer’s protocol.

### Illumina sequencing and Transcriptome (RNA-seq) data analysis

The bar-coded cDNA libraries were pooled together in equal concentrations in one pool before sequencing on Illumina HiSeq2000 using the TruSeq SR Cluster Generation Kit v3 and TruSeq SBS Kit v3. Data were processed with the Illumina Pipeline Software v1.82.

Sequence reads of the different conditions were mapped against the reference sequence of *M. bovis* AF2122/97 strain as paired end data using SMALT. The read counts were used in DESeq^86^ to first generate a distance matrix and PCA plots (Fig. S3, plotPCA function). Differential expression analysis (DESeq^86^) was performed pairwise between individual BCG strain and *M. bovis* AF2122/97 strain, using the default parameter. For the up down regulated genes of each comparison, a GO enrichment was done, using the R package TopGO, default parameter (http://www.bioconductor.org/packages/2.11/bioc/html/topGO.html). GO terms were obtained from EBI [ftp://ftp.ebi.ac.uk/pub/databases/GO/goa/proteomes/].

### Quantitative RT-PCR

The reliability of the RNA-seq expression data was assessed using quantitative real-time reverse transcriptase PCR (qRT-PCR) analysis. Briefly, 200 ng of total RNA was used to generate cDNA using SuperScript III reverse transcriptase (Invitrogen). QPCR was performed using SYBR GreenER QPCR kit (Invitrogen) and the 7900HT Fast Real-Time PCR System (Applied Biosystems). Ct values of target genes were normalized to values obtained for the mycobacterial housekeeping gene *sigA.* In total, 15 genes involved in different pathways were randomly selected for verification of RNA-seq data (Table S8).

### Whole cell lysate preparation and tandem mass tagging (TMT) quantification

For proteome analysis, *M. bovis* BCG Pasteur, Phipps, Danish, Japan and Birkhaug exponential growth phase cultures were harvested at an OD_600_ of 0.6 and washed twice and suspended in PBS for lysis. Cellular proteins were obtained by bead beating using 425–600 μm glass beads (Sigma) in the presence of a protease inhibitor (PMSF, 20 mM). The extracted proteins were quantified using the BCA protein assay (Thermo Scientific, USA). A total of 100 μg of each protein sample was reduced in tris (2-carboxyethylphosphine), alkylated using methyl-methanethiol-sulfonate (MMTS) and digested with trypsin. Tryptic digests were dried, resuspended in triethylammonium bicarbonate (pH 8.5) and labeled with 6plex TMT reagents (Thermo Scientific) according to the manufacture manual. The labeled peptides were pooled and fractionated using strong cation exchange chromatography. The LC-MS/MS analysis of the peptide fractions using an LTQ-Orbitrap Velos (Thermo Scientific) coupled with an Easy-nLC (Thermo Scientific) and the MS data processing were carried out following our published procedure^87^. Briefly, the MS spectra were extracted using Proteome Discoverer software (http://www.thermoscientific.com/en/product/proteome-discoverer-software.html) and processed by an in-house script before Mascot search against the database for *M. bovis* sequence (EMBL accession number BX248333.1). The resulting files were then processed by Scaffold (Proteome Software Inc., USA) for validation of MS/MS based peptide and protein identifications. Peptide and protein identifications were reported if they could be established at greater than 95.0% probability. Scaffold local false positive rates for peptide and protein were controlled within 1%. The peptide and protein quantitation were performed using Scaffold Q + (Proteome Software Inc).

## Additional Information

**Accession codes:** All sequencing reads and assemblies have been submitted to the EMBL-EBI European Nucleotide Archive (ENA) under the study accession PRJEB8560.

**How to cite this article**: Abdallah, A. M. *et al.* Genomic expression catalogue of a global collection of BCG vaccine strains show evidence for highly diverged metabolic and cell-wall adaptations. *Sci. Rep.* **5**, 15443; doi: 10.1038/srep15443 (2015).

## Supplementary Material

Supplementary Information

## Figures and Tables

**Table 1:** Drug sensitivity testing of *M. bovis* BCG strains.

BCG strains	Minimum inhibitory concentration (MIC, μg/ml)				
	Streptomycin	Rifampicin	Rifabutin	Ethambutol	Isoniazid
Pasteur	0.125	0.0005	0.002	0.5	1
Japan	0.125	0.0005	0.002	0.5	0.25
Tice	0.125	0.00025	0.002	0.5	1
Danish	0.015	0.000125	0.002	0.5	0,25
Phipps	0.125	0,001	0.002	1	1
Connaught	0.125	0.000125	0.002	1	1
Prague	0.03	0.000125	0.002	1	1.5
Birkhaug	—	—	—	—	—
Frappier	0.125	0.0005	0.002	1	1
Glaxo	0.125	0.00025	0.002	1	1
Russia	—	—	—	0.5	0.03
Sweden	0.03	—	0.002	0.5	0.015
Moreau	—	—	—	—	—
China	0.125	00005	0,002	1	1

The sensitivities of fourteen different BCG strains to streptomycin, rifampicin, rifabutin, ethambutol and isoniazid were determined.

## References

[b1] Frothingham R., Hills H. G. & Wilson K. H. Extensive DNA sequence conservation throughout the Mycobacterium tuberculosis complex. J Clin Microbiol 32, 1639–1643 (1994). 7929750 10.1128/jcm.32.7.1639-1643.1994 PMC263747

[b2] Imaeda T. Deoxyribonucleic-Acid Relatedness among Selected Strains of Mycobacterium-Tuberculosis, Mycobacterium-Bovis, Mycobacterium-Bovis Bcg, Mycobacterium-Microti, and Mycobacterium-Africanum. Int J Syst Bacteriol 35, 147–150 (1985).

[b3] Zwerling A. *et al.* The BCG World Atlas: a database of global BCG vaccination policies and practices. PLoS medicine 8, e1001012, 10.1371/journal.pmed.1001012 (2011). 21445325 PMC3062527

[b4] Brewer T. F. Preventing tuberculosis with bacillus Calmette-Guerin vaccine: a meta-analysis of the literature. Clinical infectious diseases: an official publication of the Infectious Diseases Society of America 31, Suppl 3, S64–67, 10.1086/314072 (2000). 11010824

[b5] Colditz G. A. *et al.* The efficacy of bacillus Calmette-Guerin vaccination of newborns and infants in the prevention of tuberculosis: meta-analyses of the published literature. Pediatrics 96, 29–35 (1995). 7596718

[b6] Trunz B. B., Fine P. & Dye C. Effect of BCG vaccination on childhood tuberculous meningitis and miliary tuberculosis worldwide: a meta-analysis and assessment of cost-effectiveness. Lancet 367, 1173–1180, 10.1016/S0140-6736(06)68507-3 (2006). 16616560

[b7] Mendoza-Coronel E., Camacho-Sandoval R., Bonifaz L. C. & Lopez-Vidal Y. PD-L2 induction on dendritic cells exposed to Mycobacterium avium downregulates BCG-specific T cell response. Tuberculosis (Edinb) 91, 36–46, 10.1016/j.tube.2010.11.008 (2011). 21147037

[b8] Brandt L. *et al.* Failure of the Mycobacterium bovis BCG vaccine: some species of environmental mycobacteria block multiplication of BCG and induction of protective immunity to tuberculosis. Infect Immun 70, 672–678 (2002). 11796598 10.1128/iai.70.2.672-678.2002 PMC127715

[b9] Lalor M. K. *et al.* Population Differences in Immune Responses to Bacille Calmette-Guerin Vaccination in Infancy. Journal of Infectious Diseases 199, 795–800, 10.1086/597069 (2009). 19434928 PMC3276835

[b10] Behr M. A. BCG—different strains, different vaccines? Lancet Infect Dis 2, 86–92 (2002). 11901655 10.1016/s1473-3099(02)00182-2

[b11] Aronson J. D., Aronson C. F. & Taylor H. C. A twenty-year appraisal of BCG vaccination in the control of tuberculosis. A.M.A. archives of internal medicine 101, 881–893 (1958). 13519929 10.1001/archinte.1958.00260170037006

[b12] Rosenthal S. R. *et al.* BCG vaccination against tuberculosis in Chicago. A twenty-year study statistically analyzed. Pediatrics 28, 622–641 (1961). 14494079

[b13] Liu J., Tran V., Leung A. S., Alexander D. C. & Zhu B. BCG vaccines: their mechanisms of attenuation and impact on safety and protective efficacy. Human vaccines 5, 70–78 (2009). 19164935 10.4161/hv.5.2.7210

[b14] Oettinger T., Jorgensen M., Ladefoged A., Haslov K. & Andersen P. Development of the Mycobacterium bovis BCG vaccine: review of the historical and biochemical evidence for a genealogical tree. Tubercle and lung disease: the official journal of the International Union against Tuberculosis and Lung Disease 79, 243–250, 10.1054/tuld.1999.0206 (1999). 10692993

[b15] Behr M. A. Correlation between BCG genomics and protective efficacy. Scand J Infect Dis 33, 249–252, 10.1080/003655401300077180 (2001). 11345214

[b16] Behr M. A. *et al.* Comparative genomics of BCG vaccines by whole-genome DNA microarray. Science 284, 1520–1523, 10.1126/Science.284.5419.1520 (1999). 10348738

[b17] Brosch R. *et al.* Comparative genomics uncovers large tandem chromosomal duplications in Mycobacterium bovis BCG Pasteur. Yeast 17, 111–123, 10.1002/1097-0061(20000630)17:2<111:AID-YEA17>3.0.CO;2-G (2000). 10900457 PMC2448323

[b18] Brosch R. *et al.* Genome plasticity of BCG and impact on vaccine efficacy. Proceedings of the National Academy of Sciences of the United States of America 104, 5596–5601, 10.1073/pnas.0700869104 (2007). 17372194 PMC1838518

[b19] Mahairas G. G., Sabo P. J., Hickey M. J., Singh D. C. & Stover C. K. Molecular analysis of genetic differences between Mycobacterium bovis BCG and virulent M-bovis. Journal of bacteriology 178, 1274–1282 (1996). 8631702 10.1128/jb.178.5.1274-1282.1996 PMC177799

[b20] Pelayo M. C. G. *et al.* A Comprehensive Survey of Single Nucleotide Polymorphisms (SNPs) across Mycobacterium bovis Strains and M. bovis BCG Vaccine Strains Refines the Genealogy and Defines a Minimal Set of SNPs That Separate Virulent M. bovis Strains and M. bovis BCG Strains. Infection and immunity 77, 2230–2238, 10.1128/Iai.01099-08 (2009). 19289514 PMC2681724

[b21] Behr M. A. & Small P. M. A historical and molecular phylogeny of BCG strains. Vaccine 17, 915–922, 10.1016/S0264-410x(98)00277-1 (1999). 10067698

[b22] Abdallah A. M. *et al.* Type VII secretion—mycobacteria show the way. Nature reviews. Microbiology 5, 883–891, 10.1038/nrmicro1773 (2007). 17922044

[b23] Lewis K. N. *et al.* Deletion of RD1 from Mycobacterium tuberculosis mimics bacille Calmette-Guerin attenuation. J Infect Dis 187, 117–123, 10.1086/345862 (2003). 12508154 PMC1458498

[b24] Pym A. S., Brodin P., Brosch R., Huerre M. & Cole S. T. Loss of RD1 contributed to the attenuation of the live tuberculosis vaccines Mycobacterium bovis BCG and Mycobacterium microti. Mol Microbiol 46, 709–717 (2002). 12410828 10.1046/j.1365-2958.2002.03237.x

[b25] Kozak R. A., Alexander D. C., Liao R., Sherman D. R. & Behr M. A. Region of difference 2 contributes to virulence of Mycobacterium tuberculosis. Infection and immunity 79, 59–66, 10.1128/IAI.00824-10 (2011). 20974821 PMC3019914

[b26] Castillo-Rodal A. I. *et al.* Mycobacterium bovis BCG substrains confer different levels of protection against Mycobacterium tuberculosis infection in a BALB/c model of progressive pulmonary tuberculosis. Infect Immun 74, 1718–1724, 10.1128/IAI.74.3.1718-1724.2006 (2006). 16495544 PMC1418655

[b27] Ritz N. *et al.* The influence of bacille Calmette-Guerin vaccine strain on the immune response against tuberculosis: a randomized trial. American journal of respiratory and critical care medicine 185, 213–222, 10.1164/rccm.201104-0714OC (2012). 22071384

[b28] Lamm D. L. Efficacy and safety of bacille Calmette-Guerin immunotherapy in superficial bladder cancer. Clinical infectious diseases: an official publication of the Infectious Diseases Society of America 31, Suppl 3, S86–90, 10.1086/314064 (2000). 11010830

[b29] Molife R. & Hancock B. W. Adjuvant therapy of malignant melanoma. Critical reviews in oncology/hematology 44, 81–102 (2002). 12399001 10.1016/s1040-8428(02)00014-8

[b30] Malmstrom P. U. *et al.* An individual patient data meta-analysis of the long-term outcome of randomised studies comparing intravesical mitomycin C versus bacillus Calmette-Guerin for non-muscle-invasive bladder cancer. European urology 56, 247–256, 10.1016/j.eururo.2009.04.038 (2009). 19409692

[b31] van der Meijden A. P. *et al.* Maintenance Bacillus Calmette-Guerin for Ta T1 bladder tumors is not associated with increased toxicity: results from a European Organisation for Research and Treatment of Cancer Genito-Urinary Group Phase III Trial. European urology 44, 429–434 (2003). 14499676 10.1016/s0302-2838(03)00357-9

[b32] Lukacs S., Tschobotko B., Szabo N. A. & Symes A. Systemic BCG-Osis as a Rare Side Effect of Intravesical BCG Treatment for Superficial Bladder Cancer. Case reports in urology 2013, 821526, 10.1155/2013/821526 (2013). 23844314 PMC3703374

[b33] Rentsch C. A. *et al.* Bacillus Calmette-Guerin strain differences have an impact on clinical outcome in bladder cancer immunotherapy. European urology 66, 677–688, 10.1016/j.eururo.2014.02.061 (2014). 24674149

[b34] Garnier T. *et al.* The complete genome sequence of Mycobacterium bovis. Proceedings of the National Academy of Sciences of the United States of America 100, 7877–7882, 10.1073/pnas.1130426100 (2003). 12788972 PMC164681

[b35] Coll F. *et al.* PolyTB: a genomic variation map for Mycobacterium tuberculosis. Tuberculosis (Edinb) 94, 346–354, 10.1016/j.tube.2014.02.005 (2014). 24637013 PMC4066953

[b36] Stewart G. R. *et al.* Dissection of the heat-shock response in Mycobacterium tuberculosis using mutants and microarrays. Microbiology 148, 3129–3138 (2002). 12368446 10.1099/00221287-148-10-3129

[b37] Stewart G. R. *et al.* Overexpression of heat-shock proteins reduces survival of Mycobacterium tuberculosis in the chronic phase of infection. Nature medicine 7, 732–737, 10.1038/89113 (2001). 11385512

[b38] Zhang W. *et al.* Genome sequencing and analysis of BCG vaccine strains. Plos One 8, e71243, 10.1371/journal.pone.0071243 (2013). 23977002 PMC3747166

[b39] Copin R., Coscolla M., Efstathiadis E., Gagneux S. & Ernst J. D. Impact of *in vitro* evolution on antigenic diversity of Mycobacterium bovis bacillus Calmette-Guerin (BCG). Vaccine 32, 5998–6004, 10.1016/j.vaccine.2014.07.113 (2014). 25211768 PMC4539939

[b40] Supply P. *et al.* Genomic analysis of smooth tubercle bacilli provides insights into ancestry and pathoadaptation of Mycobacterium tuberculosis. Nature genetics 45, 172–179, 10.1038/ng.2517 (2013). 23291586 PMC3856870

[b41] Orduna P. *et al.* Genomic and proteomic analyses of Mycobacterium bovis BCG Mexico 1931 reveal a diverse immunogenic repertoire against tuberculosis infection. Bmc Genomics 12, 493, 10.1186/1471-2164-12-493 (2011). 21981907 PMC3199284

[b42] Huard R. C. *et al.* Novel genetic polymorphisms that further delineate the phylogeny of the Mycobacterium tuberculosis complex. Journal of bacteriology 188, 4271–4287, 10.1128/Jb.01783-05 (2006). 16740934 PMC1482959

[b43] Chen J. M., Uplekar S., Gordon S. V. & Cole S. T. A point mutation in cycA partially contributes to the D-cycloserine resistance trait of Mycobacterium bovis BCG vaccine strains. Plos One 7, e43467, 10.1371/journal.pone.0043467 (2012). 22912881 PMC3422274

[b44] Kolibab K., Derrick S. C. & Morris S. L. Sensitivity to isoniazid of Mycobacterium bovis BCG strains and BCG disseminated disease isolates. Journal of clinical microbiology 49, 2380–2381, 10.1128/JCM.00648-11 (2011). 21508153 PMC3122778

[b45] Behr M. A., Schroeder B. G., Brinkman J. N., Slayden R. A. & Barry C. E. A point mutation in the mma3 gene is responsible for impaired methoxymycolic acid production in Mycobacterium bovis BCG strains obtained after 1927. Journal of bacteriology 182, 3394–3399, 10.1128/Jb.182.12.3394-3399.2000 (2000). 10852869 PMC101902

[b46] Takayama K., Wang L. & David H. L. Effect of isoniazid on the *in vivo* mycolic acid synthesis, cell growth, and viability of Mycobacterium tuberculosis. Antimicrob Agents Chemother 2, 29–35 (1972). 4208567 10.1128/aac.2.1.29 PMC444261

[b47] Keating L. A. *et al.* The pyruvate requirement of some members of the Mycobacterium tuberculosis complex is due to an inactive pyruvate kinase: implications for *in vivo* growth. Mol Microbiol 56, 163–174, 10.1111/J.1365-2958.2005.04524.X (2005). 15773987

[b48] Makinoshima H. & Glickman M. S. Regulation of Mycobacterium tuberculosis cell envelope composition and virulence by intramembrane proteolysis. Nature 436, 406–409, 10.1038/nature03713 (2005). 16034419 PMC1502149

[b49] Sassetti C. M. & Rubin E. J. Genetic requirements for mycobacterial survival during infection. Proceedings of the National Academy of Sciences of the United States of America 100, 12989–12994, 10.1073/pnas.2134250100 (2003). 14569030 PMC240732

[b50] Manganelli R., Dubnau E., Tyagi S., Kramer F. R. & Smith I. Differential expression of 10 sigma factor genes in Mycobacterium tuberculosis. Mol Microbiol 31, 715–724 (1999). 10027986 10.1046/j.1365-2958.1999.01212.x

[b51] Gomez J. E. & Bishai W. R. whmD is an essential mycobacterial gene required for proper septation and cell division. Proceedings of the National Academy of Sciences of the United States of America 97, 8554–8559, 10.1073/Pnas.140225297 (2000). 10880571 PMC26986

[b52] Morris R. P. *et al.* Ancestral antibiotic resistance in Mycobacterium tuberculosis. Proceedings of the National Academy of Sciences of the United States of America 102, 12200–12205, 10.1073/pnas.0505446102 (2005). 16103351 PMC1186028

[b53] Steyn A. J. C. *et al.* Mycobacterium tuberculosis WhiB3 interacts with RpoV to affect host survival but is dispensable for *in vivo* growth. Proceedings of the National Academy of Sciences of the United States of America 99, 3147–3152, 10.1073/Pnas.052705399 (2002). 11880648 PMC122487

[b54] Casali N., White A. M. & Riley L. W. Regulation of the Mycobacterium tuberculosis mce1 operon. Journal of bacteriology 188, 441–449, 10.1128/JB.188.2.441-449.2006 (2006). 16385033 PMC1347267

[b55] Charlet D. *et al.* Reduced expression of antigenic proteins MPB70 and MPB83 in Mycobacterium bovis BCG strains due to a start codon mutation in sigK. Molecular microbiology 56, 1302–1313 (2005). 15882422 10.1111/j.1365-2958.2005.04618.x

[b56] Sampson S. L. Mycobacterial PE/PPE Proteins at the Host-Pathogen Interface. Clin Dev Immunol, Artn 497203, doi 10.1155/2011/497203 (2011). PMC3034920 21318182

[b57] Leung A. S. *et al.* Novel genome polymorphisms in BCG vaccine strains and impact on efficacy. BMC genomics 9, 413, 10.1186/1471-2164-9-413 (2008). 18793412 PMC2553098

[b58] Ates L. S. *et al.* Essential Role of the ESX-5 Secretion System in Outer Membrane Permeability of Pathogenic Mycobacteria. PLoS Genet 11, e1005190, 10.1371/journal.pgen.1005190 (2015). 25938982 PMC4418733

[b59] Lew J. M., Kapopoulou A., Jones L. M. & Cole S. T. TubercuList—10 years after. Tuberculosis (Edinb) 91, 1–7, 10.1016/j.tube.2010.09.008 (2011). 20980199

[b60] Armitige L. Y., Jagannath C., Wanger A. R. & Norris S. J. Disruption of the genes encoding antigen 85A and antigen 85B of Mycobacterium tuberculosis H37Rv: effect on growth in culture and in macrophages. Infection and immunity 68, 767–778 (2000). 10639445 10.1128/iai.68.2.767-778.2000 PMC97204

[b61] Heym B. *et al.* Effects of overexpression of the alkyl hydroperoxide reductase AhpC on the virulence and isoniazid resistance of Mycobacterium tuberculosis. Infection and immunity 65, 1395–1401 (1997). 9119479 10.1128/iai.65.4.1395-1401.1997 PMC175145

[b62] Liu K., Yu J. & Russell D. G. pckA-deficient Mycobacterium bovis BCG shows attenuated virulence in mice and in macrophages. Microbiology 149, 1829–1835 (2003). 12855734 10.1099/mic.0.26234-0

[b63] Daffe M. & Draper P. The envelope layers of mycobacteria with reference to their pathogenicity. Adv Microb Physiol 39, 131–203 (1998). 9328647 10.1016/s0065-2911(08)60016-8

[b64] Behr M. A. BCG—different strains, different vaccines? The Lancet infectious diseases 2, 86–92 (2002). 11901655 10.1016/s1473-3099(02)00182-2

[b65] Matsunaga I. *et al.* Mycobacterium tuberculosis pks12 produces a novel polyketide presented by CD1c to T cells. The Journal of experimental medicine 200, 1559–1569, 10.1084/jem.20041429 (2004). 15611286 PMC2211992

[b66] Matsunaga I., Maeda S., Nakata N. & Fujiwara N. The polyketide synthase-associated multidrug tolerance in Mycobacterium intracellulare clinical isolates. Chemotherapy 58, 341–348, 10.1159/000343311 (2012). 23171694

[b67] Av-Gay Y., Jamil S. & Drews S. J. Expression and characterization of the Mycobacterium tuberculosis serine/threonine protein kinase PknB. Infection and immunity 67, 5676–5682 (1999). 10531215 10.1128/iai.67.11.5676-5682.1999 PMC96941

[b68] Cole S. T. *et al.* Deciphering the biology of Mycobacterium tuberculosis from the complete genome sequence. Nature 393, 537–544, 10.1038/31159 (1998). 9634230

[b69] Berthet F. X. *et al.* Attenuation of virulence by disruption of the Mycobacterium tuberculosis erp gene. Science 282, 759–762 (1998). 9784137 10.1126/science.282.5389.759

[b70] Youmans A. S. & Youmans G. P. Immunogenic Activity of a Ribosomal Fraction Obtained from Mycobacterium Tuberculosis. Journal of bacteriology 89, 1291-& (1965). 14293000 10.1128/jb.89.5.1291-1298.1965 PMC277642

[b71] Tantimavanich S. *et al.* Immunological properties of ribosomal proteins from Mycobacterium bovis BCG. Infection and immunity 61, 4005–4007 (1993). 8359926 10.1128/iai.61.9.4005-4007.1993 PMC281109

[b72] Miyazaki C. *et al.* Host immune responses to ribosome, ribosomal proteins, and RNA from Mycobacterium bovis bacille de Calmette-Guerin. Vaccine 17, 245–251 (1999). 9987160 10.1016/s0264-410x(98)00191-1

[b73] Sinha S. *et al.* Immunogenic membrane-associated proteins of Mycobacterium tuberculosis revealed by proteomics. Microbiology 151, 2411–2419, 10.1099/mic.0.27799-0 (2005). 16000731

[b74] Gu S. *et al.* Comprehensive proteomic profiling of the membrane constituents of a Mycobacterium tuberculosis strain. Mol Cell Proteomics 2, 1284–1296, 10.1074/Mcp.M300060-Mcp200 (2003). 14532352

[b75] Beste D. J. V. *et al.* Compiling a molecular inventory for Mycobacterium bovis BCG at two growth rates: Evidence for growth rate-mediated regulation of ribosome biosynthesis and lipid metabolism. Journal of bacteriology 187, 1677–1684, 10.1128/Jb.157.5.1677-1684.2005 (2005). 15716438 PMC1064002

[b76] Lewthwaite J. C. *et al.* Mycobacterium tuberculosis chaperonin 60.1 is a more potent cytokine stimulator than chaperonin 60.2 (Hsp 65) and contains a CD14-binding domain. Infection and immunity 69, 7349–7355, 10.1128/IAI.69.12.7349-7355.2001 (2001). 11705907 PMC98821

[b77] Bonato V. L., Lima V. M., Tascon R. E., Lowrie D. B. & Silva C. L. Identification and characterization of protective T cells in hsp65 DNA-vaccinated and Mycobacterium tuberculosis-infected mice. Infection and immunity 66, 169–175 (1998). 9423854 10.1128/iai.66.1.169-175.1998 PMC107873

[b78] Dubos R. J. & Pierce C. H. Differential characteristics *in vitro* and *in vivo* of several substrains of BCG. IV. Immunizing effectiveness. American review of tuberculosis 74, 699–717 (1956). 13372955 10.1164/artpd.1956.74.5.699

[b79] Lagranderie M. R., Balazuc A. M., Deriaud E., Leclerc C. D. & Gheorghiu M. Comparison of immune responses of mice immunized with five different Mycobacterium bovis BCG vaccine strains. Infection and immunity 64, 1–9 (1996). 8557324 10.1128/iai.64.1.1-9.1996 PMC173719

[b80] Bolger A. M., Lohse M. & Usadel B. Trimmomatic: a flexible trimmer for Illumina sequence data. Bioinformatics, 10.1093/bioinformatics/btu170 (2014). PMC4103590 24695404

[b81] Zerbino D. R. & Birney E. Velvet: Algorithms for *de novo* short read assembly using de Bruijn graphs. Genome research 18, 821–829, 10.1101/Gr.074492.107 (2008). 18349386 PMC2336801

[b82] Swain M. T. *et al.* A post-assembly genome-improvement toolkit (PAGIT) to obtain annotated genomes from contigs. Nature protocols 7, 1260–1284, 10.1038/nprot.2012.068 (2012). 22678431 PMC3648784

[b83] Li H. & Durbin R. Fast and accurate short read alignment with Burrows-Wheeler transform. Bioinformatics 25, 1754–1760, 10.1093/bioinformatics/btp324 (2009). 19451168 PMC2705234

[b84] Li H. *et al.* The Sequence Alignment/Map format and SAMtools. Bioinformatics 25, 2078–2079, 10.1093/bioinformatics/btp352 (2009). 19505943 PMC2723002

[b85] Wang J. *et al.* CREST maps somatic structural variation in cancer genomes with base-pair resolution. Nature methods 8, 652–654, 10.1038/nmeth.1628 (2011). 21666668 PMC3527068

[b86] Anders S. & Huber W. Differential expression analysis for sequence count data. Genome biology 11, R106, 10.1186/gb-2010-11-10-r106 (2010). 20979621 PMC3218662

[b87] Liu P., Zhang H. M., Wang H. & Xia Y. J. Identification of redox-sensitive cysteines in the Arabidopsis proteome using OxiTRAQ, a quantitative redox proteomics method. Proteomics 14, 750–762, 10.1002/Pmic.201300307 (2014). 24376095

