# Supplementary material for: Genomic expression catalogue of a global collection of BCG vaccine strains show evidence for highly diverged metabolic and cell-wall adaptations
Source: Sci Rep. 2015 Oct 21;5:15443. doi: 10.1038/srep15443 (PMC4614345; doi:10.1038/srep15443)
Supplement: Supplementary Information [file srep15443-s1.pdf]

Genomic expression catalogue of a global collection of BCG vaccine strains show evidence for highly diverged metabolic and cell-wall adaptations.

Abdallah M. Abdallah[1‡*], Grant A. Hill-Cawthorne[1,2‡], Thomas D. Otto[3], Francesc Coll[4], José Afonso Guerra-Assunção[4], Ge Gao[1], Raeece Naeem[1], Hifzur Ansari[1], Tareq B. Malas[1], Sabir A. Adroub[1], Theo Verboom[5], Roy Ummels[5], Huoming Zhang[6], Aswini Kumar Panigrahi[6], Ruth McNerney[4], Roland Brosch[7], Taane G. Clark[4,8], Marcel A. Behr[9], Wilbert Bitter[5], Arnab Pain[1,10*]

Supplementary Tables and Figures legends

Table S1: Numbers of SNPs, small Indels and Unique Variants identified in *M. bovis* and fourteen BCG strains compared to *M. bovis* AF2122/97[1].

Table S2: List of identified SNPs in fourteen BCG strains compared to *M. bovis* AF2122/97[1] with their types.

Table S3: Distribution of regions of difference and deleted genes in the genomes of fourteen BCG strains compared to *M. bovis* AF2122/97[1].

Table S4: The lengths and positions of *leuA* gene insertions in *M. bovis* AF2122/97[1] and BCG strains.

Table S5: Sequences insertion in fourteen BCG strains compared to *M. bovis* AF2122/97[1]. Insertions were detected by performing *de novo* assembly and aligning resulting contigs to the reference genome (*M. bovis* AF2122/97[1]).

Table S6: Pearson correlation coefficients between the biological replicates of RNA-seq transcriptome data. Analysis was done in R with cor function, using the log read counts.

Table S7: Proteomic profiling of BCG Japan, Birkahug, Pasteur, Phipps and Danish compared to *M. bovis*.

Table S8: Oligonucleotide primers used for reverse-transcription quantitative PCR. The table shows the gene symbol for the investigated transcripts and sequences for forward/backward primer.

Figure S1: nRD18 deletion from BCG china strains (this study and Zhang et al.,[2]) confirmed by ACT comparison[3,4] with reference *M. bovis.* An ACT screenshot is shown with BlastN comparison.

Figure S2: Sequence duplication across different regions shown using Artemis Bam view[3] confirmed by CNVnator[5]. Individual gene annotations are shown. A) Direct repeat at *CRISPR-Cas* locus on strains BCG japan (blue), BCG Moreau (green), BCG Russia (red) and *M. bovis* (grey). B,C) Sequence duplication in BCG Frappier (pink) strain compared with

reference *M.bovis* (green). D) Sequence duplication across all BCG strains.

Figure S3: Principal component cluster analysis (PCA) of biological replicates of RNA-seq transcriptome data. PCA mapping showed clustering of biological replicates from each strain of the fourteen BCG strains and *M. bovis* AF2122/97[1] strain. The graph was done with plotPCA function of DESeq[6] using the read counts.

Figure S4: Expression profiles for all genes, showing significant changes in gene expression were clustered using the Euclidean distance algorithm.

Figure S5: Confirmation of differential gene expression during RNA-seq analysis by Q-RT-PCR. Scatterplot of the relationship between genes quantified in both data sets. Scatterplots display the rectilinear equation and coefficient of determination (R2).

Figure S6. Gene ontology (GO) analysis of the differentially expressed genes.

Heat maps representing GO enrichment of (a) Biological Processes, (b) Molecular Functions and (c) Cellular Components for all differentially expressed genes. The GO terms were clustered by their enrichment patterns ($p<0.001$). Using the Pearson correlation as the distance measure with average linkage. Up-regulation is indicated in red, down-regulation in green and was calculated with DESeq[6], see Methods.

Figure S7: Expression of genes encoding proteins predicted to be involved in energy metabolism. Genes predicted to be involved in energy generation and NAD^+ regeneration were selected based on their annotation[1]. The color scales represent log2-fold changes in gene expression (DESeq[6]), using the *M. bovis* AF2122/97[1] strain as reference.

Figure S8: Transcriptional profile of a global collection of fourteen BCG strains: Gene expression heat maps of genes showing expression profiles in: (A) PE family. (B) PE_PGRS family. (C) PPE family. The color scales represent log2-fold changes in gene expression (DESeq[6]), using the *M. bovis* AF2122/97[1] strain as reference.

Figure S9: Transcriptional profile of T cell antigen present in 14 BCG strains. Selected genes contain experimentally verified list of human T-cell antigens[2,7]. The color scales represent log2-fold changes in gene expression (DESeq[6]), using the *M. bovis* AF2122/97[1] strain as reference.

Figure S10: Global proteomic profiling of BCG Japan, Birkahug, Pasteur, Phipps and Danish compared to *M. bovis* AF2122/97[1]. Proteins displaying high differential regulation (2-fold change differences) were grouped according to the BoviList classification (http://genolist.pasteur.fr/ BoviList/).

References

1 Garnier, T. *et al.* The complete genome sequence of Mycobacterium bovis. *Proceedings of the National Academy of Sciences of the United States of America* **100**, 7877-7882, doi:10.1073/pnas.1130426100 (2003).

2 Zhang, W. *et al.* Genome sequencing and analysis of BCG vaccine strains. *Plos One* **8**, e71243, doi:10.1371/journal.pone.0071243 (2013).

3 Carver, T. *et al.* Artemis and ACT: viewing, annotating and comparing sequences stored in a relational database. *Bioinformatics* **24**, 2672-2676, doi:Doi 10.1093/Bioinformatics/Btn529 (2008).

4 Carver, T. J. *et al.* ACT: the Artemis Comparison Tool. *Bioinformatics* **21**, 3422-3423, doi:10.1093/bioinformatics/bti553 (2005).

5 Abyzov, A., Urban, A. E., Snyder, M. & Gerstein, M. CNVnator: an approach to discover, genotype, and characterize typical and atypical CNVs from family and population genome sequencing. *Genome research* **21**, 974-984, doi:10.1101/gr.114876.110 (2011).

6 Anders, S. & Huber, W. Differential expression analysis for sequence count data. *Genome biology* **11**, R106, doi:10.1186/gb-2010-11-10-r106 (2010).

7 Comas, I. *et al.* Human T cell epitopes of Mycobacterium tuberculosis are evolutionarily hyperconserved. *Nature genetics* **42**, 498-503, doi:10.1038/ng.590 (2010).

Table S1: numbers of SNPs, small Indels and Unique variants identified in this study

Strain	No. SNPs	No. Indels	No. unique variants
M. bovis	9	12	5
Birkhaug	770	92	15
Connaught	764	81	5
Danish	756	81	10
Frappier	772	54	13
Glaxo	774	94	16
Moreau	789	94	8
Pasteur	760	88	14
Phipps	778	100	0
Prague	762	76	19
Russia	750	73	37
Sweden	774	91	20
Tice	779	95	9
Japan	757	81	11

Table S2: List of identified SNPs in fourteen BCG strains compared to M. bovis AF2122/97 with their types.

Position_in_alignment	Ref_base	SNP_base	Total	birkhaug	china	connaught	Danish	frappier	glaxo	moreau	pasteur	phipps	prague	russia	sweden	tice	Japan
766	G	A	2	A	.	.	.	.	.	.	.	.	.	.	A	.	.
1057	A	G	14	G	G	G	G	G	G	G	G	G	G	G	G	G	G
4480	T	C	14	C	C	C	C	C	C	C	C	C	C	C	C	C	C
8624	G	T	14	T	T	T	T	T	T	T	T	T	T	T	T	T	T
9217	C	A	14	A	A	A	A	A	A	A	A	A	A	A	A	A	A
10727	G	A	14	A	A	A	A	A	A	A	A	A	A	A	A	A	A
17856	C	T	14	T	T	T	T	T	T	T	T	T	T	T	T	T	T
24020	C	T	2	T	.	.	.	.	.	.	.	.	.	.	T	.	.
24678	G	A	14	A	A	A	A	A	A	A	A	A	A	A	A	A	A
25287	G	C	14	C	C	C	C	C	C	C	C	C	C	C	C	C	C
27695	C	T	14	T	T	T	T	T	T	T	T	T	T	T	T	T	T
33788	A	G	12	G	G	G	G	G	G	G	G	N	G	G	G	N	G
35326	C	T	14	T	T	T	T	T	T	T	T	T	T	T	T	T	T
39077	G	A	14	A	A	A	A	A	A	A	A	A	A	A	A	A	A
39257	G	A	3	.	A	.	.	.	.	.	A	A	.	.	.	.	.
41228	A	C	14	C	C	C	C	C	C	C	C	C	C	C	C	C	C
41437	T	C	14	C	C	C	C	C	C	C	C	C	C	C	C	C	C
49989	A	G	14	G	G	G	G	G	G	G	G	G	G	G	G	G	G
50750	A	G	14	G	G	G	G	G	G	G	G	G	G	G	G	G	G
59864	A	G	14	G	G	G	G	G	G	G	G	G	G	G	G	G	G
65759	A	C	14	C	C	C	C	C	C	C	C	C	C	C	C	C	C
65860	A	G	14	G	G	G	G	G	G	G	G	G	G	G	G	G	G
69065	C	T	14	T	T	T	T	T	T	T	T	T	T	T	T	T	T
69916	T	C	14	C	C	C	C	C	C	C	C	C	C	C	C	C	C
70085	G	A	14	A	A	A	A	A	A	A	A	A	A	A	A	A	A
85016	A	G	14	G	G	G	G	G	G	G	G	G	G	G	G	G	G
87123	T	C	14	C	C	C	C	C	C	C	C	C	C	C	C	C	C

89168	C	T	4	.	T	.	.	.	.	.	T	T	.	.	.	T	.
90973	T	C	14	C	C	C	C	C	C	C	C	C	C	C	C	C	C
114138	C	A	14	A	A	A	A	A	A	A	A	A	A	A	A	A	A
126088	G	C	14	C	C	C	C	C	C	C	C	C	C	C	C	C	C
127456	G	C	14	C	C	C	C	C	C	C	C	C	C	C	C	C	C
128310	A	G	14	G	G	G	G	G	G	G	G	G	G	G	G	G	G
130246	T	C	14	C	C	C	C	C	C	C	C	C	C	C	C	C	C
138794	A	C,T	1,1	N	-	N	N	N	N	N	T	-	N	-	C	-	N
146015	A	G	14	G	G	G	G	G	G	G	G	G	G	G	G	G	G
151692	C	T	1	.	.	T	.	.	.	.	.	.	.	.	.	.	.
159382	A	G	14	G	G	G	G	G	G	G	G	G	G	G	G	G	G
160547	T	C	14	C	C	C	C	C	C	C	C	C	C	C	C	C	C
161134	C	T	1	.	.	.	N	.	T	.	.	.	.	.	.	.	.
161554	C	A	14	A	A	A	A	A	A	A	A	A	A	A	A	A	A
162511	A	G	14	G	G	G	G	G	G	G	G	G	G	G	G	G	G
165814	G	A	14	A	A	A	A	A	A	A	A	A	A	A	A	A	A
166711	A	G	14	G	G	G	G	G	G	G	G	G	G	G	G	G	G
173106	A	C	14	C	C	C	C	C	C	C	C	C	C	C	C	C	C
175034	C	T	1	.	.	.	.	.	.	.	.	.	.	T	.	.	.
177551	C	T	1	.	.	.	.	.	.	.	.	.	.	T	.	.	.
192192	A	G	14	G	G	G	G	G	G	G	G	G	G	G	G	G	G
193219	T	C	14	C	C	C	C	C	C	C	C	C	C	C	C	C	C
201049	C	T	1	.	.	.	.	T	.	.	.	.	.	.	.	.	.
208512	A	G	14	G	G	G	G	G	G	G	G	G	G	G	G	G	G
214592	C	A	1	.	.	A	.	.	.	.	.	.	.	.	.	.	.
217986	T	G	14	G	G	G	G	G	G	G	G	G	G	G	G	G	G
219351	C	T	14	T	T	T	T	T	T	T	T	T	T	T	T	T	T
224649	T	C	14	C	C	C	C	C	C	C	C	C	C	C	C	C	C
227230	G	A	14	A	A	A	A	A	A	A	A	A	A	A	A	A	A
232203	G	C	14	C	C	C	C	C	C	C	C	C	C	C	C	C	C

234188	C	T	14	T	T	T	T	T	T	T	T	T	T	T	T	T	T
237784	C	G	1	.	.	.	.	.	.	.	.	.	.	G	.	.	.
241987	A	C	14	C	C	C	C	C	C	C	C	C	C	C	C	C	C
247471	A	G	1	.	.	.	.	.	.	.	.	.	.	G	.	.	.
253243	C	T	14	T	T	T	T	T	T	T	T	T	T	T	T	T	T
258016	C	A	14	A	A	A	A	A	A	A	A	A	A	A	A	A	A
258368	T	G	14	G	G	G	G	G	G	G	G	G	G	G	G	G	G
264261	T	C	14	C	C	C	C	C	C	C	C	C	C	C	C	C	C
265198	A	G	14	G	G	G	G	G	G	G	G	G	G	G	G	G	G
270004	T	C	14	C	C	C	C	C	C	C	C	C	C	C	C	C	C
287291	T	C	14	C	C	C	C	C	C	C	C	C	C	C	C	C	C
288793	T	C	14	C	C	C	C	C	C	C	C	C	C	C	C	C	C
292467	C	T	14	T	T	T	T	T	T	T	T	T	T	T	T	T	T
293378	G	A	1	A	.	.	.	.	.	.	.	.	.	.	.	.	.
295534	T	C	14	C	C	C	C	C	C	C	C	C	C	C	C	C	C
302955	G	A	1	.	.	.	.	.	.	.	.	.	.	A	.	.	.
311426	C	T	14	T	T	T	T	T	T	T	T	T	T	T	T	T	T
314906	T	C	14	C	C	C	C	C	C	C	C	C	C	C	C	C	C
316408	C	T	14	T	T	T	T	T	T	T	T	T	T	T	T	T	T
321024	T	C	14	C	C	C	C	C	C	C	C	C	C	C	C	C	C
323242	C	G	14	G	G	G	G	G	G	G	G	G	G	G	G	G	G
327558	A	G	14	G	G	G	G	G	G	G	G	G	G	G	G	G	G
331688	A	G	14	G	G	G	G	G	G	G	G	G	G	G	G	G	G
331857	G	C	14	C	C	C	C	C	C	C	C	C	C	C	C	C	C
331860	T	C	14	C	C	C	C	C	C	C	C	C	C	C	C	C	C
334335	T	C	7	N	C	N	C	N	N	N	C	C	N	C	N	C	C
334338	T	C	7	N	C	N	C	N	N	N	C	C	N	C	N	C	C
336428	C	T	1	.	.	.	.	.	.	N	.	.	.	T	.	.	.
340112	G	A	6	N	A	N	A	N	N	N	N	A	N	A	A	A	N
340114	C	T	8	N	T	N	T	N	T	T	N	T	N	T	T	T	N

340115	G	A	9	N	A	N	A	N	A	A	N	A	N	A	A	A	A
340116	G	C	10	N	C	N	C	C	C	C	N	C	N	C	C	C	C
340121	C	G	10	N	G	N	G	N	G	G	G	G	N	G	G	G	G
340369	T	C	2	.	.	.	.	.	.	.	C	C	.	.	.	.	.
343713	C	T	1	.	.	.	.	T	.	.	.	.	.	.	.	.	.
347063	A	G	14	G	G	G	G	G	G	G	G	G	G	G	G	G	G
354475	G	A	2	.	.	A	.	A	.	.	.	.	.	.	.	.	.
357750	A	G	1	.	.	.	.	.	.	G	.	.	.	.	.	.	.
358307	C	T	14	T	T	T	T	T	T	T	T	T	T	T	T	T	T
362845	T	C	14	C	C	C	C	C	C	C	C	C	C	C	C	C	C
364587	A	G	14	G	G	G	G	G	G	G	G	G	G	G	G	G	G
364831	T	C	14	C	C	C	C	C	C	C	C	C	C	C	C	C	C
366052	T	C	14	C	C	C	C	C	C	C	C	C	C	C	C	C	C
373205	G	A	14	A	A	A	A	A	A	A	A	A	A	A	A	A	A
375726	C	T	4	-	-	-	-	-	-	T	-	-	T	T	-	T	-
375731	C	G	3	-	-	-	-	-	-	N	-	-	G	G	-	G	-
375733	G	T	4	-	-	-	-	-	-	T	-	-	T	T	-	T	-
375741	C	T	1	-	-	-	-	-	-	N	-	-	N	N	-	T	-
375742	C	T	4	-	-	-	-	-	-	T	-	-	T	T	-	T	-
375743	G	C	1	-	-	-	-	-	-	N	-	-	N	N	-	C	-
391010	T	C	2	C	.	.	.	.	.	.	.	.	.	.	C	.	.
400720	T	G	14	G	G	G	G	G	G	G	G	G	G	G	G	G	G
406041	C	T	1	.	.	.	.	T	.	.	.	.	.	.	.	.	.
408567	T	C	14	C	C	C	C	C	C	C	C	C	C	C	C	C	C
409575	G	A	14	A	A	A	A	A	A	A	A	A	A	A	A	A	A
424351	A	G	14	G	G	G	G	G	G	G	G	G	G	G	G	G	G
425104	C	T	10	T	T	T	N	T	T	T	T	N	T	-	T	-	T
425105	G	T	10	T	T	T	N	T	T	T	T	N	T	-	T	-	T
425175	A	C	8	N	N	C	C	C	C	C	N	N	C	-	C	-	C
430110	C	T	14	T	T	T	T	T	T	T	T	T	T	T	T	T	T

438266	A	G	1	.	.	.	.	G	.	.	.	.	.	.	.	.	.
438515	C	G	14	G	G	G	G	G	G	G	G	G	G	G	G	G	G
441795	C	T	14	T	T	T	T	T	T	T	T	T	T	T	T	T	T
446465	T	C	14	C	C	C	C	C	C	C	C	C	C	C	C	C	C
449955	T	C	14	C	C	C	C	C	C	C	C	C	C	C	C	C	C
463872	G	T	1	.	.	.	.	.	.	.	.	.	.	T	.	.	.
467376	C	T	14	T	T	T	T	T	T	T	T	T	T	T	T	T	T
467435	C	A	14	A	A	A	A	A	A	A	A	A	A	A	A	A	A
467973	C	T	13	T	T	T	T	T	T	N	T	T	T	T	T	T	T
471265	C	G	2	G	.	.	N	.	.	.	.	.	.	.	G	.	.
479012	C	T	1	.	.	.	.	T	.	.	.	.	.	.	.	.	.
485925	G	T	14	T	T	T	T	T	T	T	T	T	T	T	T	T	T
488930	G	T	14	T	T	T	T	T	T	T	T	T	T	T	T	T	T
490911	G	A	14	A	A	A	A	A	A	A	A	A	A	A	A	A	A
495322	C	T	14	T	T	T	T	T	T	T	T	T	T	T	T	T	T
502672	T	C	14	C	C	C	C	C	C	C	C	C	C	C	C	C	C
506354	G	A	14	A	A	A	A	A	A	A	A	A	A	A	A	A	A
507962	T	C	14	C	C	C	C	C	C	C	C	C	C	C	C	C	C
519445	T	C	14	C	C	C	C	C	C	C	C	C	C	C	C	C	C
520858	A	G	14	G	G	G	G	G	G	G	G	G	G	G	G	G	G
521005	C	G	1	.	.	.	.	.	.	.	.	.	.	.	.	G	.
532602	C	G	14	G	G	G	G	G	G	G	G	G	G	G	G	G	G
535446	C	T	8	.	T	T	N	T	T	.	T	T	T	.	.	T	.
541604	T	C	14	C	C	C	C	C	C	C	C	C	C	C	C	C	C
544213	C	T	14	T	T	T	T	T	T	T	T	T	T	T	T	T	T
556382	T	C	14	C	C	C	C	C	C	C	C	C	C	C	C	C	C
559772	C	T	14	T	T	T	T	T	T	T	T	T	T	T	T	T	T
560205	C	T	14	T	T	T	T	T	T	T	T	T	T	T	T	T	T
562304	T	G	5	G	.	.	.	.	.	G	.	.	.	G	G	.	G
563995	C	T	14	T	T	T	T	T	T	T	T	T	T	T	T	T	T

566207	A	C	14	C	C	C	C	C	C	C	C	C	C	C	C	C	C	C
580728	T	C	14	C	C	C	C	C	C	C	C	C	C	C	C	C	C	C
580982	C	T	1	T	.	.	.	.	.	.	.	.	.	.	.	.	.	.
581977	A	G	3	N	N	G	N	G	N	N	N	N	G	N	N	N	N	
581981	G	A	6	N	N	A	N	A	N	N	A	A	N	N	A	N	A	
582044	G	A	14	A	A	A	A	A	A	A	A	A	A	A	A	A	A	
582647	C	T	1	.	.	.	.	.	.	.	.	.	.	.	.	.	T	
589160	G	T	9	.	T	T	T	T	T	.	T	T	T	.	.	T	.	
593243	C	A	14	A	A	A	A	A	A	A	A	A	A	A	A	A	A	
598737	C	G	14	G	G	G	G	G	G	G	G	G	G	G	G	G	G	
604016	G	T	1	.	.	.	.	.	.	.	.	.	T	.	.	.	.	
607400	C	G	14	G	G	G	G	G	G	G	G	G	G	G	G	G	G	
611110	A	G	14	G	G	G	G	G	G	G	G	G	G	G	G	G	G	
622422	C	G	14	G	G	G	G	G	G	G	G	G	G	G	G	G	G	
634621	C	T	1	T	.	.	.	.	.	.	.	.	.	.	.	.	.	
644281	C	G	14	G	G	G	G	G	G	G	G	G	G	G	G	G	G	
644598	T	C	9	.	C	C	C	C	C	.	C	C	C	.	N	C	.	
649946	T	C	14	C	C	C	C	C	C	C	C	C	C	C	C	C	C	
651605	G	A	1	.	.	.	.	.	.	.	.	.	A	.	.	.	.	
661087	G	A	14	A	A	A	A	A	A	A	A	A	A	A	A	A	A	
665323	G	T	14	T	T	T	T	T	T	T	T	T	T	T	T	T	T	
673916	T	C	13	C	C	C	C	C	C	N	C	C	C	C	C	C	C	
680452	C	T	14	T	T	T	T	T	T	T	T	T	T	T	T	T	T	
683991	G	T	14	T	T	T	T	T	T	T	T	T	T	T	T	T	T	
684243	T	C	14	C	C	C	C	C	C	C	C	C	C	C	C	C	C	
685105	T	C	14	C	C	C	C	C	C	C	C	C	C	C	C	C	C	
693872	T	C	14	C	C	C	C	C	C	C	C	C	C	C	C	C	C	
699139	T	C	14	C	C	C	C	C	C	C	C	C	C	C	C	C	C	
699970	C	A	2	A	.	.	.	.	.	.	.	.	.	.	A	.	.	
701368	G	C	14	C	C	C	C	C	C	C	C	C	C	C	C	C	C	

701425	A	G	14	G	G	G	G	G	G	G	G	G	G	G	G	G	G
702227	C	T	14	T	T	T	T	T	T	T	T	T	T	T	T	T	T
712358	T	C	14	C	C	C	C	C	C	C	C	C	C	C	C	C	C
713366	G	C	14	C	C	C	C	C	C	C	C	C	C	C	C	C	C
723209	G	A	14	A	A	A	A	A	A	A	A	A	A	A	A	A	A
724751	C	A	1	.	.	A	.	.	.	.	.	.	.	.	.	.	.
724757	C	T	14	T	T	T	T	T	T	T	T	T	T	T	T	T	T
727018	A	G	14	G	G	G	G	G	G	G	G	G	G	G	G	G	G
730013	C	T	3	.	T	.	.	.	.	.	T	T	.	.	.	.	.
739132	C	T	8	.	T	T	N	T	T	.	T	T	T	.	.	T	.
758828	C	T	1	.	.	.	.	.	.	.	.	.	.	.	T	.	.
765380	T	C	1	.	.	.	.	.	.	.	.	.	.	.	.	.	C
765383	G	C	14	C	C	C	C	C	C	C	C	C	C	C	C	C	C
765424	A	G	1	.	.	.	.	.	.	G	.	.	.	.	.	.	.
777573	C	T	1	.	.	.	.	.	.	.	.	.	.	.	.	.	T
783376	C	T	14	T	T	T	T	T	T	T	T	T	T	T	T	T	T
795252	C	T	1	.	.	.	.	T	.	.	.	.	.	.	.	.	.
799176	A	G	1	.	.	.	.	.	.	.	.	.	G	.	.	.	.
805036	T	C	14	C	C	C	C	C	C	C	C	C	C	C	C	C	C
808491	T	C	14	C	C	C	C	C	C	C	C	C	C	C	C	C	C
808640	T	G	14	G	G	G	G	G	G	G	G	G	G	G	G	G	G
811776	C	A	14	A	A	A	A	A	A	A	A	A	A	A	A	A	A
812748	G	A	14	A	A	A	A	A	A	A	A	A	A	A	A	A	A
814323	A	G	1	.	.	.	.	.	.	.	.	.	.	.	.	.	G
815155	A	G	1	.	.	.	.	G	.	.	.	.	.	.	.	.	.
824686	T	C	14	C	C	C	C	C	C	C	C	C	C	C	C	C	C
828045	C	G	14	G	G	G	G	G	G	G	G	G	G	G	G	G	G
836878	T	A	14	A	A	A	A	A	A	A	A	A	A	A	A	A	A
837241	C	T	14	T	T	T	T	T	T	T	T	T	T	T	T	T	T
839350	A	G	14	G	G	G	G	G	G	G	G	G	G	G	G	G	G

842411	A	G	11	G	G	G	G	G	G	N	N	G	G	G	G	G	N
842413	C	G	11	G	G	G	G	G	G	N	N	G	G	G	G	G	N
842419	G	C	2	N	N	C	N	N	N	N	N	N	N	N	C	N	N
843726	C	G	14	G	G	G	G	G	G	G	G	G	G	G	G	G	G
843727	A	G	14	G	G	G	G	G	G	G	G	G	G	G	G	G	G
844682	A	C	14	C	C	C	C	C	C	C	C	C	C	C	C	C	C
846861	C	A	14	A	A	A	A	A	A	A	A	A	A	A	A	A	A
851865	G	A	14	A	A	A	A	A	A	A	A	A	A	A	A	A	A
853865	C	T	1	.	.	.	.	T	.	N	.	.	.	N	.	.	.
854933	A	C	2	.	.	C	.	C	.	.	.	.	.	.	.	.	.
855291	C	A	1	.	.	.	.	.	.	.	.	.	.	A	.	.	.
855592	A	G	1	.	.	.	.	.	.	G	.	.	.	.	.	.	.
855928	T	C	1	C	.	.	.	.	.	.	.	.	.	.	-	.	.
857788	C	T	14	T	T	T	T	T	T	T	T	T	T	T	T	T	T
863336	G	A	1	.	.	.	.	.	A	.	.	.	.	.	.	.	.
865863	G	T	14	T	T	T	T	T	T	T	T	T	T	T	T	T	T
866406	A	G	1	.	.	.	.	.	.	G	.	.	.	.	.	.	.
866748	C	T	14	T	T	T	T	T	T	T	T	T	T	T	T	T	T
870158	G	T	14	T	T	T	T	T	T	T	T	T	T	T	T	T	T
874662	A	G	14	G	G	G	G	G	G	G	G	G	G	G	G	G	G
878256	C	A	2	A	.	.	.	.	.	.	.	.	.	.	A	.	.
901490	G	A	1	.	.	.	.	.	.	.	.	.	A	.	.	.	.
905954	C	G	14	G	G	G	G	G	G	G	G	G	G	G	G	G	G
917301	C	A	14	A	A	A	A	A	A	A	A	A	A	A	A	A	A
920703	T	C	14	C	C	C	C	C	C	C	C	C	C	C	C	C	C
920795	G	A	14	A	A	A	A	A	A	A	A	A	A	A	A	A	A
941116	A	C	14	C	C	C	C	C	C	C	C	C	C	C	C	C	C
941685	C	T	1	.	.	.	.	T	.	.	.	.	.	.	.	.	.
941693	G	C	14	C	C	C	C	C	C	C	C	C	C	C	C	C	C
943767	G	A	14	A	A	A	A	A	A	A	A	A	A	A	A	A	A

946150	G	T	14	T	T	T	T	T	T	T	T	T	T	T	T	T	T
947761	C	T	14	T	T	T	T	T	T	T	T	T	T	T	T	T	T
948070	T	C	14	C	C	C	C	C	C	C	C	C	C	C	C	C	C
949022	A	C	14	C	C	C	C	C	C	C	C	C	C	C	C	C	C
953816	C	T	1	.	.	.	.	.	.	.	.	.	.	T	.	.	.
962690	C	G	14	G	G	G	G	G	G	G	G	G	G	G	G	G	G
965549	C	T	6	N	T	T	N	N	N	N	T	T	N	T	N	T	N
968037	T	C	14	C	C	C	C	C	C	C	C	C	C	C	C	C	C
974658	G	A	14	A	A	A	A	A	A	A	A	A	A	A	A	A	A
975228	C	T	1	.	.	.	.	.	.	T	.	.	.	.	.	.	.
976381	C	T	14	T	T	T	T	T	T	T	T	T	T	T	T	T	T
979550	C	T	1	.	.	.	.	.	.	.	.	.	T	.	.	.	.
983981	C	T	14	T	T	T	T	T	T	T	T	T	T	T	T	T	T
998237	C	G	14	G	G	G	G	G	G	G	G	G	G	G	G	G	G
1018551	A	G	14	G	G	G	G	G	G	G	G	G	G	G	G	G	G
1018597	C	G	1	.	.	.	.	.	.	.	.	.	G	.	.	.	.
1031742	C	G	14	G	G	G	G	G	G	G	G	G	G	G	G	G	G
1035948	A	G	1	.	.	G	.	.	.	.	.	.	.	.	.	.	.
1040453	T	C	14	C	C	C	C	C	C	C	C	C	C	C	C	C	C
1048354	T	C	14	C	C	C	C	C	C	C	C	C	C	C	C	C	C
1065317	T	C	14	C	C	C	C	C	C	C	C	C	C	C	C	C	C
1075828	C	T	14	T	T	T	T	T	T	T	T	T	T	T	T	T	T
1096161	T	C	13	C	C	C	C	C	C	N	C	C	C	C	C	C	C
1101564	C	A	14	A	A	A	A	A	A	A	A	A	A	A	A	A	A
1104057	T	C	14	C	C	C	C	C	C	C	C	C	C	C	C	C	C
1104329	A	G	14	G	G	G	G	G	G	G	G	G	G	G	G	G	G
1106124	G	A	14	A	A	A	A	A	A	A	A	A	A	A	A	A	A
1107737	G	T	1	.	.	.	.	.	.	T	.	.	.	.	.	.	.
1107809	G	A	1	.	.	A	.	.	.	.	.	.	.	.	.	.	.
1109286	C	T	14	T	T	T	T	T	T	T	T	T	T	T	T	T	T

1112776	C	T	14	T	T	T	T	T	T	T	T	T	T	T	T	T	T
1113175	G	A	14	A	A	A	A	A	A	A	A	A	A	A	A	A	A
1124304	G	A	14	A	A	A	A	A	A	A	A	A	A	A	A	A	A
1126362	G	A	14	A	A	A	A	A	A	A	A	A	A	A	A	A	A
1129123	A	G	1	.	.	.	.	.	.	G	.	.	.	.	.	.	.
1132869	G	A	1	.	.	.	.	.	A	.	.	.	.	.	.	.	.
1135158	G	A	1	.	.	.	.	.	.	.	.	.	.	A	.	.	.
1136964	T	C	1	.	.	.	.	.	.	.	.	.	C	.	.	.	.
1137838	G	C	14	C	C	C	C	C	C	C	C	C	C	C	C	C	C
1138095	C	T	14	T	T	T	T	T	T	T	T	T	T	T	T	T	T
1139527	T	C	14	C	C	C	C	C	C	C	C	C	C	C	C	C	C
1149874	T	C	14	C	C	C	C	C	C	C	C	C	C	C	C	C	C
1151953	G	A	14	A	A	A	A	A	A	A	A	A	A	A	A	A	A
1152219	T	C	14	C	C	C	C	C	C	C	C	C	C	C	C	C	C
1158891	G	C	2	C	.	.	.	.	.	.	.	.	.	.	C	.	.
1159134	T	G	1	G	.	.	.	.	.	.	.	.	.	.	N	.	.
1162461	G	T	14	T	T	T	T	T	T	T	T	T	T	T	T	T	T
1166534	A	C	14	C	C	C	C	C	C	C	C	C	C	C	C	C	C
1168496	G	T	14	T	T	T	T	T	T	T	T	T	T	T	T	T	T
1172152	C	T	14	T	T	T	T	T	T	T	T	T	T	T	T	T	T
1172559	T	G	14	G	G	G	G	G	G	G	G	G	G	G	G	G	G
1186582	G	A	2	A	.	.	.	.	.	.	.	.	.	.	A	.	.
1192663	A	G	4	N	G	N	G	N	N	N	N	G	N	-	N	-	G
1192667	C	A	2	N	A	N	N	N	N	N	N	A	N	-	N	-	N
1192668	G	C	2	N	C	N	N	N	N	N	N	C	N	-	N	-	N
1192750	T	C	2	N	C	N	N	N	N	N	N	C	N	-	N	-	N
1194258	G	A	14	A	A	A	A	A	A	A	A	A	A	A	A	A	A
1197934	C	T	14	T	T	T	T	T	T	T	T	T	T	T	T	T	T
1199567	G	A	14	A	A	A	A	A	A	A	A	A	A	A	A	A	A
1199568	A	C	14	C	C	C	C	C	C	C	C	C	C	C	C	C	C

1206934	A	G	14	G	G	G	G	G	G	G	G	G	G	G	G	G	G
1214578	T	C	14	C	C	C	C	C	C	C	C	C	C	C	C	C	C
1214912	A	G	1	-	N	-	-	-	-	N	-	G	-	N	-	N	-
1214914	C	T	1	-	N	-	-	-	-	N	-	N	-	N	-	T	-
1216529	C	A	14	A	A	A	A	A	A	A	A	A	A	A	A	A	A
1228662	A	C	1	.	.	.	.	.	.	.	.	.	.	.	.	.	C
1230187	A	C	14	C	C	C	C	C	C	C	C	C	C	C	C	C	C
1230919	G	T	14	T	T	T	T	T	T	T	T	T	T	T	T	T	T
1237100	C	T	14	T	T	T	T	T	T	T	T	T	T	T	T	T	T
1241020	T	C	1	.	.	C	.	.	.	.	.	.	.	.	.	.	.
1244790	G	C	14	C	C	C	C	C	C	C	C	C	C	C	C	C	C
1247460	G	A	1	.	.	A	.	.	.	.	.	.	.	.	.	.	.
1259294	T	C	14	C	C	C	C	C	C	C	C	C	C	C	C	C	C
1277052	G	T	14	T	T	T	T	T	T	T	T	T	T	T	T	T	T
1278032	C	G	11	G	G	N	G	G	G	G	N	G	G	G	G	G	N
1278133	G	T	14	T	T	T	T	T	T	T	T	T	T	T	T	T	T
1283094	C	T	14	T	T	T	T	T	T	T	T	T	T	T	T	T	T
1284974	C	G	6	G	-	N	G	G	G	G	N	-	N	-	N	-	G
1284976	C	G	9	G	-	G	G	G	G	G	N	-	G	-	G	-	G
1286510	A	G	1	.	.	.	.	.	.	.	.	.	.	.	.	G	.
1286634	G	C	1	.	.	C	.	.	.	.	.	.	.	.	.	.	.
1294561	C	T	2	.	.	.	.	.	.	.	T	T	.	.	.	.	.
1295845	T	C	14	C	C	C	C	C	C	C	C	C	C	C	C	C	C
1295906	G	C	1	.	.	.	.	.	.	.	.	.	.	.	.	.	C
1319319	G	C	14	C	C	C	C	C	C	C	C	C	C	C	C	C	C
1335142	T	C	8	C	N	N	C	N	C	C	N	N	C	C	C	N	C
1339858	C	G	6	G	-	G	N	-	-	G	G	-	G	-	G	-	N
1339859	A	C	6	C	-	C	N	-	-	C	C	-	C	-	C	-	N
1340159	C	A	14	A	A	A	A	A	A	A	A	A	A	A	A	A	A
1340295	G	T	2	.	.	T	.	.	.	.	.	.	T	.	.	.	.

1340760	G	C	4	N	C	N	N	N	N	N	N	C	N	C	N	C	N
1340763	T	G	4	N	G	N	N	N	N	N	N	G	N	G	N	G	N
1340764	C	A	4	N	A	N	N	N	N	N	N	A	N	A	N	A	N
1341041	T	C	2	N	N	N	N	N	N	N	N	C	N	N	N	C	N
1341042	A	C	2	N	N	N	N	N	N	N	N	C	N	N	N	C	N
1341222	C	T	1	N	N	N	N	N	N	N	N	N	N	T	N	N	N
1341367	G	A	1	N	N	N	N	N	N	N	N	N	N	N	N	A	N
1341388	A	C	4	N	C	N	N	N	N	N	N	C	N	C	N	C	N
1341399	G	C	3	N	N	N	N	N	N	N	N	C	N	C	N	C	N
1341409	G	C	4	N	C	N	N	N	N	N	N	C	N	C	N	C	N
1341410	C	T	4	N	T	N	N	N	N	N	N	T	N	T	N	T	N
1341451	A	G	14	G	G	G	G	G	G	G	G	G	G	G	G	G	G
1341455	A	G	14	G	G	G	G	G	G	G	G	G	G	G	G	G	G
1341638	A	C	14	C	C	C	C	C	C	C	C	C	C	C	C	C	C
1341639	G	A	14	A	A	A	A	A	A	A	A	A	A	A	A	A	A
1341642	C	A	14	A	A	A	A	A	A	A	A	A	A	A	A	A	A
1341830	A	G	1	.	.	.	.	.	.	.	.	.	G	.	.	.	.
1342100	C	T	4	N	T	N	N	N	N	N	N	T	N	T	N	T	N
1342103	C	T	4	N	T	N	N	N	N	N	N	T	N	T	N	T	N
1342106	G	C	4	N	C	N	N	N	N	N	N	C	N	C	N	C	N
1342109	C	T	4	N	T	N	N	N	N	N	N	T	N	T	N	T	N
1342155	A	G	14	G	G	G	G	G	G	G	G	G	G	G	G	G	G
1346066	G	A	14	A	A	A	A	A	A	A	A	A	A	A	A	A	A
1347553	G	A	14	A	A	A	A	A	A	A	A	A	A	A	A	A	A
1355971	A	C	14	C	C	C	C	C	C	C	C	C	C	C	C	C	C
1357694	C	T	1	T	.	.	.	.	.	.	.	.	.	.	.	.	.
1362701	T	C	14	C	C	C	C	C	C	C	C	C	C	C	C	C	C
1364349	C	G	14	G	G	G	G	G	G	G	G	G	G	G	G	G	G
1366287	A	C	14	C	C	C	C	C	C	C	C	C	C	C	C	C	C
1372934	C	A	14	A	A	A	A	A	A	A	A	A	A	A	A	A	A

1383787	C	T	14	T	T	T	T	T	T	T	T	T	T	T	T	T	T
1412880	T	C	14	C	C	C	C	C	C	C	C	C	C	C	C	C	C
1412884	T	A	14	A	A	A	A	A	A	A	A	A	A	A	A	A	A
1412949	T	A	14	A	A	A	A	A	A	A	A	A	A	A	A	A	A
1417579	T	C	14	C	C	C	C	C	C	C	C	C	C	C	C	C	C
1428709	G	A	1	.	A	.	.	.	.	.	.	.	.	.	.	.	.
1437869	T	G	14	G	G	G	G	G	G	G	G	G	G	G	G	G	G
1438168	T	C	14	C	C	C	C	C	C	C	C	C	C	C	C	C	C
1453340	C	G	14	G	G	G	G	G	G	G	G	G	G	G	G	G	G
1455059	C	G	14	G	G	G	G	G	G	G	G	G	G	G	G	G	G
1459820	G	A	1	.	.	.	.	.	.	A	.	.	.	.	.	.	.
1460353	C	G	14	G	G	G	G	G	G	G	G	G	G	G	G	G	G
1464554	G	C	1	.	.	.	.	.	.	.	.	.	.	C	.	.	.
1477005	T	C	14	C	C	C	C	C	C	C	C	C	C	C	C	C	C
1488093	C	T	14	T	T	T	T	T	T	T	T	T	T	T	T	T	T
1497610	T	C	14	C	C	C	C	C	C	C	C	C	C	C	C	C	C
1499347	G	T	14	T	T	T	T	T	T	T	T	T	T	T	T	T	T
1503657	G	T	14	T	T	T	T	T	T	T	T	T	T	T	T	T	T
1504076	G	A	2	A	.	.	.	.	.	.	.	.	.	.	A	.	.
1505165	A	G	14	G	G	G	G	G	G	G	G	G	G	G	G	G	G
1523512	G	C	14	C	C	C	C	C	C	C	C	C	C	C	C	C	C
1524825	T	G	14	G	G	G	G	G	G	G	G	G	G	G	G	G	G
1528853	A	G	14	G	G	G	G	G	G	G	G	G	G	G	G	G	G
1530977	T	C	14	C	C	C	C	C	C	C	C	C	C	C	C	C	C
1530981	T	C	14	C	C	C	C	C	C	C	C	C	C	C	C	C	C
1531044	T	G	14	G	G	G	G	G	G	G	G	G	G	G	G	G	G
1531065	C	T	3	N	N	N	N	N	N	N	T	N	N	N	N	T	T
1533051	T	G	14	G	G	G	G	G	G	G	G	G	G	G	G	G	G
1537630	A	G	14	G	G	G	G	G	G	G	G	G	G	G	G	G	G
1541225	A	G	14	G	G	G	G	G	G	G	G	G	G	G	G	G	G

1541814	A	C	14	C	C	C	C	C	C	C	C	C	C	C	C	C	C
1541890	A	G	14	G	G	G	G	G	G	G	G	G	G	G	G	G	G
1545565	C	G	14	G	G	G	G	G	G	G	G	G	G	G	G	G	G
1549399	T	C	14	C	C	C	C	C	C	C	C	C	C	C	C	C	C
1558180	A	G	14	G	G	G	G	G	G	G	G	G	G	G	G	G	G
1561667	C	G	14	G	G	G	G	G	G	G	G	G	G	G	G	G	G
1563771	G	A	14	A	A	A	A	A	A	A	A	A	A	A	A	A	A
1563878	C	T	14	T	T	T	T	T	T	T	T	T	T	T	T	T	T
1564177	G	A	14	A	A	A	A	A	A	A	A	A	A	A	A	A	A
1572071	G	C	14	C	C	C	C	C	C	C	C	C	C	C	C	C	C
1593534	T	C	14	C	C	C	C	C	C	C	C	C	C	C	C	C	C
1602554	A	G	14	G	G	G	G	G	G	G	G	G	G	G	G	G	G
1612487	T	C	14	C	C	C	C	C	C	C	C	C	C	C	C	C	C
1614834	A	G	14	G	G	G	G	G	G	G	G	G	G	G	G	G	G
1615042	T	G	13	G	G	G	G	G	G	N	G	G	G	G	G	G	G
1619181	C	T	14	T	T	T	T	T	T	T	T	T	T	T	T	T	T
1619531	A	G	3	N	N	-	-	-	G	N	-	-	G	N	G	-	N
1619532	A	G	5	G	N	-	-	-	G	N	-	-	G	G	G	-	N
1628006	C	G	13	G	G	G	G	G	G	N	G	G	G	G	G	G	G
1628126	C	A	1	.	.	.	.	.	.	N	.	.	.	A	.	.	.
1628550	G	T	14	T	T	T	T	T	T	T	T	T	T	T	T	T	T
1629670	G	A	2	A	.	.	.	.	.	N	.	.	.	.	A	.	.
1633592	C	G	10	G	G	N	G	G	G	N	N	N	G	G	G	G	G
1633593	C	T	5	N	T	N	T	N	N	N	N	N	T	T	N	T	N
1633601	C	T	1	N	N	N	N	N	N	N	N	N	N	T	N	N	N
1633602	C	G	1	N	N	N	N	N	N	N	N	N	N	G	N	N	N
1643613	A	T	14	T	T	T	T	T	T	T	T	T	T	T	T	T	T
1643953	C	T	14	T	T	T	T	T	T	T	T	T	T	T	T	T	T
1647716	C	T	14	T	T	T	T	T	T	T	T	T	T	T	T	T	T
1657488	T	G	14	G	G	G	G	G	G	G	G	G	G	G	G	G	G

1657489	T	A	14	A	A	A	A	A	A	A	A	A	A	A	A	A	A
1661122	G	A	14	A	A	A	A	A	A	A	A	A	A	A	A	A	A
1668972	A	G	14	G	G	G	G	G	G	G	G	G	G	G	G	G	G
1670857	G	A	2	.	.	A	.	A	.	.	.	.	.	.	.	.	.
1683144	C	T	14	T	T	T	T	T	T	T	T	T	T	T	T	T	T
1687823	C	G	14	G	G	G	G	G	G	G	G	G	G	G	G	G	G
1695445	G	A	14	A	A	A	A	A	A	A	A	A	A	A	A	A	A
1698882	T	C	14	C	C	C	C	C	C	C	C	C	C	C	C	C	C
1701112	C	T	14	T	T	T	T	T	T	T	T	T	T	T	T	T	T
1707806	C	A	14	A	A	A	A	A	A	A	A	A	A	A	A	A	A
1712773	T	C	14	C	C	C	C	C	C	C	C	C	C	C	C	C	C
1714476	A	G	14	G	G	G	G	G	G	G	G	G	G	G	G	G	G
1715907	A	G	14	G	G	G	G	G	G	G	G	G	G	G	G	G	G
1716470	C	T	14	T	T	T	T	T	T	T	T	T	T	T	T	T	T
1730257	T	G	2	.	-	N	.	G	.	.	G	-	.	.	.	-	.
1730263	A	C	3	.	-	C	.	C	.	.	C	-	.	.	.	-	.
1733255	T	C	14	C	C	C	C	C	C	C	C	C	C	C	C	C	C
1737240	G	A	14	A	A	A	A	A	A	A	A	A	A	A	A	A	A
1741652	A	G	14	G	G	G	G	G	G	G	G	G	G	G	G	G	G
1741691	A	T	3	T	-	N	N	N	N	T	T	-	N	-	N	-	-
1752501	T	G	8	G	-	G	N	G	G	G	N	-	G	-	G	-	G
1763721	C	T	14	T	T	T	T	T	T	T	T	T	T	T	T	T	T
1784032	C	A	14	A	A	A	A	A	A	A	A	A	A	A	A	A	A
1785983	G	A	14	A	A	A	A	A	A	A	A	A	A	A	A	A	A
1798722	C	T	14	T	T	T	T	T	T	T	T	T	T	T	T	T	T
1800684	T	C	14	C	C	C	C	C	C	C	C	C	C	C	C	C	C
1802310	T	G	14	G	G	G	G	G	G	G	G	G	G	G	G	G	G
1812766	C	T	14	T	T	T	T	T	T	T	T	T	T	T	T	T	T
1813945	C	T	1	.	T	.	.	.	.	.	.	.	.	.	.	.	.
1815746	G	A	14	A	A	A	A	A	A	A	A	A	A	A	A	A	A

1818210	A	G	1	.	.	.	.	.	.	.	.	.	.	.	.	G	.
1823749	A	T	14	T	T	T	T	T	T	T	T	T	T	T	T	T	T
1824666	G	C	14	C	C	C	C	C	C	C	C	C	C	C	C	C	C
1829017	A	G	14	G	G	G	G	G	G	G	G	G	G	G	G	G	G
1835191	G	T	14	T	T	T	T	T	T	T	T	T	T	T	T	T	T
1837833	G	T	14	T	T	T	T	T	T	T	T	T	T	T	T	T	T
1863716	A	G	14	G	G	G	G	G	G	G	G	G	G	G	G	G	G
1869993	A	C	14	C	C	C	C	C	C	C	C	C	C	C	C	C	C
1882356	A	G	14	G	G	G	G	G	G	G	G	G	G	G	G	G	G
1890609	G	C	14	C	C	C	C	C	C	C	C	C	C	C	C	C	C
1891804	T	C	14	C	C	C	C	C	C	C	C	C	C	C	C	C	C
1911148	G	C	14	C	C	C	C	C	C	C	C	C	C	C	C	C	C
1911623	A	G	14	G	G	G	G	G	G	G	G	G	G	G	G	G	G
1915017	C	T	14	T	T	T	T	T	T	T	T	T	T	T	T	T	T
1915787	T	C	14	C	C	C	C	C	C	C	C	C	C	C	C	C	C
1916530	C	T	14	T	T	T	T	T	T	T	T	T	T	T	T	T	T
1918087	T	G	3	.	G	.	.	.	.	.	G	G	.	.	.	.	.
1923270	C	T	1	.	.	.	.	.	.	T	.	.	.	.	.	.	.
1928659	A	G	14	G	G	G	G	G	G	G	G	G	G	G	G	G	G
1935920	G	A	14	A	A	A	A	A	A	A	A	A	A	A	A	A	A
1937468	C	G	14	G	G	G	G	G	G	G	G	G	G	G	G	G	G
1938771	A	T	14	T	T	T	T	T	T	T	T	T	T	T	T	T	T
1941052	G	C	6	C	N	C	N	N	C	C	N	N	C	N	C	N	N
1941053	G	A	7	A	N	A	N	A	N	A	A	N	A	N	A	N	N
1941129	G	C	11	C	N	C	N	C	C	C	C	N	C	C	C	C	C
1941131	C	A	13	A	A	A	A	A	A	A	A	N	A	A	A	A	A
1946275	C	T	14	T	T	T	T	T	T	T	T	T	T	T	T	T	T
1953616	C	T	1	.	.	.	.	T	.	.	.	.	.	.	.	.	.
1953665	T	G	14	G	G	G	G	G	G	G	G	G	G	G	G	G	G
1954664	A	T	14	T	T	T	T	T	T	T	T	T	T	T	T	T	T

1957343	G	A	14	A	A	A	A	A	A	A	A	A	A	A	A	A	A
1962741	C	A	14	A	A	A	A	A	A	A	A	A	A	A	A	A	A
1966429	G	C	14	C	C	C	C	C	C	C	C	C	C	C	C	C	C
1970522	T	C	14	C	C	C	C	C	C	C	C	C	C	C	C	C	C
1981005	G	C	1	-	C	-	-	-	-	-	-	N	-	N	-	N	-
1981007	A	C	1	-	N	-	-	-	-	-	-	C	-	N	-	N	-
1981012	T	C	4	-	C	-	-	-	-	-	-	C	-	C	-	C	-
1981036	C	T	14	T	T	T	T	T	T	T	T	T	T	T	T	T	T
1986879	C	T	14	T	T	T	T	T	T	T	T	T	T	T	T	T	T
1988836	C	T	9	N	T	T	T	N	T	T	N	N	T	T	N	T	T
1997748	A	G	12	G	G	G	G	G	G	G	N	N	G	G	G	G	G
2002990	C	G	14	G	G	G	G	G	G	G	G	G	G	G	G	G	G
2003321	C	T	14	T	T	T	T	T	T	T	T	T	T	T	T	T	T
2007997	C	T	14	T	T	T	T	T	T	T	T	T	T	T	T	T	T
2010718	G	T	1	.	.	.	.	.	.	.	.	.	T	.	.	.	.
2011751	G	A	5	A	.	.	N	.	.	A	.	.	.	A	A	.	A
2011885	C	T	8	.	T	T	N	T	T	.	T	T	T	.	.	T	.
2016562	C	G	1	.	.	.	.	.	.	.	.	.	.	G	.	.	.
2016867	G	C	2	C	.	.	.	.	.	.	.	.	.	.	C	.	.
2017327	A	G	14	G	G	G	G	G	G	G	G	G	G	G	G	G	G
2018160	G	T	14	T	T	T	T	T	T	T	T	T	T	T	T	T	T
2018168	G	A	14	A	A	A	A	A	A	A	A	A	A	A	A	A	A
2019543	T	C	14	C	C	C	C	C	C	C	C	C	C	C	C	C	C
2019740	G	A	13	A	A	A	A	A	A	A	A	A	N	A	A	A	A
2020577	T	C	14	C	C	C	C	C	C	C	C	C	C	C	C	C	C
2021574	G	T	1	.	.	.	.	.	.	.	.	.	.	.	.	.	T
2022655	C	T	1	.	.	T	.	.	.	.	.	.	.	.	.	.	.
2022834	T	G	2	.	.	G	.	G	.	.	.	.	.	.	.	.	.
2023556	G	A	14	A	A	A	A	A	A	A	A	A	A	A	A	A	A
2023614	C	G	1	.	.	.	.	.	.	.	.	.	.	.	G	.	.

2023772	A	G	1	.	.	.	.	.	.	.	.	.	.	G	.	.	.
2025428	G	T	1	.	.	.	.	.	.	.	.	.	.	T	.	.	.
2027378	C	T	1	.	.	.	.	.	.	.	.	.	.	T	.	.	.
2031297	A	G	14	G	G	G	G	G	G	G	G	G	G	G	G	G	G
2033637	C	T	14	T	T	T	T	T	T	T	T	T	T	T	T	T	T
2040046	T	C	14	C	C	C	C	C	C	C	C	C	C	C	C	C	C
2044858	A	G	14	G	G	G	G	G	G	G	G	G	G	G	G	G	G
2047356	G	A	14	A	A	A	A	A	A	A	A	A	A	A	A	A	A
2053240	G	A	14	A	A	A	A	A	A	A	A	A	A	A	A	A	A
2054936	G	A	14	A	A	A	A	A	A	A	A	A	A	A	A	A	A
2055607	A	G	14	G	G	G	G	G	G	G	G	G	G	G	G	G	G
2056796	T	C	1	.	C	.	.	.	.	.	.	.	.	.	.	.	.
2059444	G	T	14	T	T	T	T	T	T	T	T	T	T	T	T	T	T
2064779	T	C	14	C	C	C	C	C	C	C	C	C	C	C	C	C	C
2071092	C	T	14	T	T	T	T	T	T	T	T	T	T	T	T	T	T
2085810	A	G	14	G	G	G	G	G	G	G	G	G	G	G	G	G	G
2092414	C	G	14	G	G	G	G	G	G	G	G	G	G	G	G	G	G
2092501	A	G	14	G	G	G	G	G	G	G	G	G	G	G	G	G	G
2094732	T	C	14	C	C	C	C	C	C	C	C	C	C	C	C	C	C
2094786	C	T	14	T	T	T	T	T	T	T	T	T	T	T	T	T	T
2105046	T	C	14	C	C	C	C	C	C	C	C	C	C	C	C	C	C
2109516	A	G	14	G	G	G	G	G	G	G	G	G	G	G	G	G	G
2110994	A	G	14	G	G	G	G	G	G	G	G	G	G	G	G	G	G
2111179	G	A	1	.	.	.	.	.	.	.	.	.	.	A	.	.	.
2113785	A	G	14	G	G	G	G	G	G	G	G	G	G	G	G	G	G
2119153	A	G	14	G	G	G	G	G	G	G	G	G	G	G	G	G	G
2120818	G	A	1	A	.	.	.	.	.	.	.	.	.	.	.	.	.
2125774	A	G	14	G	G	G	G	G	G	G	G	G	G	G	G	G	G
2126943	A	G	14	G	G	G	G	G	G	G	G	G	G	G	G	G	G
2135038	T	C	14	C	C	C	C	C	C	C	C	C	C	C	C	C	C

2137504	T	C	14	C	C	C	C	C	C	C	C	C	C	C	C	C	C
2140668	G	A	1	N	.	.	N	.	.	.	.	.	.	.	A	.	.
2141560	T	C	14	C	C	C	C	C	C	C	C	C	C	C	C	C	C
2146847	G	A	14	A	A	A	A	A	A	A	A	A	A	A	A	A	A
2149591	C	T	14	T	T	T	T	T	T	T	T	T	T	T	T	T	T
2153888	A	C	3	N	C	N	N	N	N	N	N	N	N	C	N	C	N
2153889	T	C	4	N	C	N	N	N	N	N	N	C	N	C	N	C	N
2160055	A	G	14	G	G	G	G	G	G	G	G	G	G	G	G	G	G
2160657	C	A	14	A	A	A	A	A	A	A	A	A	A	A	A	A	A
2178859	G	C	1	.	.	.	.	.	.	.	.	.	.	.	.	.	C
2179757	G	A	1	.	.	.	.	.	.	.	.	.	A	.	.	.	.
2187664	T	C	14	C	C	C	C	C	C	C	C	C	C	C	C	C	C
2187665	A	C	14	C	C	C	C	C	C	C	C	C	C	C	C	C	C
2189006	G	C	1	N	N	N	N	N	N	N	N	C	N	N	N	N	N
2189008	C	A	1	N	N	N	N	N	N	N	N	A	N	N	N	N	N
2189012	T	C	2	N	C	N	N	N	N	N	N	C	N	N	N	N	N
2189014	G	C	2	N	C	N	N	N	N	N	N	C	N	N	N	N	N
2189020	T	C	5	N	C	N	C	N	C	C	N	C	N	N	N	N	N
2189029	A	G	8	G	G	N	G	N	G	G	N	G	G	N	G	N	N
2189032	C	A	14	A	A	A	A	A	A	A	A	A	A	A	A	A	A
2189035	C	A	14	A	A	A	A	A	A	A	A	A	A	A	A	A	A
2200088	C	G	2	.	N	N	N	N	N	.	N	G	G	.	.	N	.
2200359	G	A	6	A	N	N	A	N	N	A	N	N	N	A	A	N	A
2219596	A	C	14	C	C	C	C	C	C	C	C	C	C	C	C	C	C
2224503	G	A	1	A	.	.	.	.	.	.	.	.	.	.	.	.	.
2226609	C	T	2	T	.	.	.	.	.	.	.	.	.	.	T	.	.
2226929	G	A	14	A	A	A	A	A	A	A	A	A	A	A	A	A	A
2233245	G	A	14	A	A	A	A	A	A	A	A	A	A	A	A	A	A
2234759	C	T	14	T	T	T	T	T	T	T	T	T	T	T	T	T	T
2239982	C	G	5	N	-	G	-	-	-	G	-	-	G	-	G	-	G

2248846	T	C	14	C	C	C	C	C	C	C	C	C	C	C	C	C	C
2253244	C	A	14	A	A	A	A	A	A	A	A	A	A	A	A	A	A
2254057	C	T	2	T	.	.	.	.	.	.	.	.	.	.	T	.	.
2255848	G	A	14	A	A	A	A	A	A	A	A	A	A	A	A	A	A
2271512	A	G	14	G	G	G	G	G	G	G	G	G	G	G	G	G	G
2278906	A	G	14	G	G	G	G	G	G	G	G	G	G	G	G	G	G
2278924	C	G	13	G	G	G	G	G	G	G	G	G	G	G	G	G	N
2278926	A	G	13	G	G	G	G	G	G	G	G	G	G	G	G	G	N
2278933	A	G	14	G	G	G	G	G	G	G	G	G	G	G	G	G	G
2278941	G	A	14	A	A	A	A	A	A	A	A	A	A	A	A	A	A
2278942	G	C	14	C	C	C	C	C	C	C	C	C	C	C	C	C	C
2279056	C	T	14	T	T	T	T	T	T	T	T	T	T	T	T	T	T
2279059	G	T	14	T	T	T	T	T	T	T	T	T	T	T	T	T	T
2279061	G	C	14	C	C	C	C	C	C	C	C	C	C	C	C	C	C
2279068	A	T	14	T	T	T	T	T	T	T	T	T	T	T	T	T	T
2279074	G	C	7	N	C	N	C	N	C	N	N	C	C	C	N	C	N
2279077	C	A	14	A	A	A	A	A	A	A	A	A	A	A	A	A	A
2281733	G	A	14	A	A	A	A	A	A	A	A	A	A	A	A	A	A
2282125	G	T	14	T	T	T	T	T	T	T	T	T	T	T	T	T	T
2282223	T	C	14	C	C	C	C	C	C	C	C	C	C	C	C	C	C
2302722	G	A	14	A	A	A	A	A	A	A	A	A	A	A	A	A	A
2310843	G	A	14	A	A	A	A	A	A	A	A	A	A	A	A	A	A
2319314	C	T	1	.	.	.	.	T	.	.	.	.	.	.	.	.	.
2321432	G	C	8	N	N	C	N	C	N	C	C	C	C	N	C	N	C
2321433	T	C	8	N	N	C	N	C	N	C	N	C	N	C	C	C	C
2321454	G	C	14	C	C	C	C	C	C	C	C	C	C	C	C	C	C
2321455	T	C	13	C	C	C	C	C	C	C	C	N	C	C	C	C	C
2329700	T	C	14	C	C	C	C	C	C	C	C	C	C	C	C	C	C
2335476	A	G	14	G	G	G	G	G	G	G	G	G	G	G	G	G	G
2337007	G	A	1	.	.	.	.	A	.	.	.	.	.	.	.	.	.

2338058	C	T	1	T	.	.	.	.	.	.	.	.	.	.	.	.	.
2354004	T	C	14	C	C	C	C	C	C	C	C	C	C	C	C	C	C
2361571	G	T	1	.	.	.	.	.	.	T	.	.	.	.	.	.	.
2365551	C	A	14	A	A	A	A	A	A	A	A	A	A	A	A	A	A
2374030	C	T	14	T	T	T	T	T	T	T	T	T	T	T	T	T	T
2375728	G	A	1	.	.	.	.	A	.	.	.	.	.	.	.	.	.
2377143	C	A	14	A	A	A	A	A	A	A	A	A	A	A	A	A	A
2378970	A	G	14	G	G	G	G	G	G	G	G	G	G	G	G	G	G
2379340	C	T	14	T	T	T	T	T	T	T	T	T	T	T	T	T	T
2382446	C	G	4	N	G	N	N	N	N	N	N	G	N	G	N	G	N
2382459	C	T	4	N	T	N	N	N	N	N	N	T	N	T	N	T	N
2382608	C	T	14	T	T	T	T	T	T	T	T	T	T	T	T	T	T
2383136	G	A	14	A	A	A	A	A	A	A	A	A	A	A	A	A	A
2405495	A	G	14	G	G	G	G	G	G	G	G	G	G	G	G	G	G
2408146	G	A	14	A	A	A	A	A	A	A	A	A	A	A	A	A	A
2413976	G	A	14	A	A	A	A	A	A	A	A	A	A	A	A	A	A
2424109	C	A	14	A	A	A	A	A	A	A	A	A	A	A	A	A	A
2442854	G	A	2	.	.	.	N	.	A	.	.	.	A	.	.	.	.
2450607	C	A	14	A	A	A	A	A	A	A	A	A	A	A	A	A	A
2458267	T	C	14	C	C	C	C	C	C	C	C	C	C	C	C	C	C
2462783	C	G	14	G	G	G	G	G	G	G	G	G	G	G	G	G	G
2473751	C	A	14	A	A	A	A	A	A	A	A	A	A	A	A	A	A
2474719	C	T	14	T	T	T	T	T	T	T	T	T	T	T	T	T	T
2478193	A	G	14	G	G	G	G	G	G	G	G	G	G	G	G	G	G
2488915	A	G	14	G	G	G	G	G	G	G	G	G	G	G	G	G	G
2492734	C	G	14	G	G	G	G	G	G	G	G	G	G	G	G	G	G
2495661	G	A	14	A	A	A	A	A	A	A	A	A	A	A	A	A	A
2501914	A	C	14	C	C	C	C	C	C	C	C	C	C	C	C	C	C
2504341	G	T	14	T	T	T	T	T	T	T	T	T	T	T	T	T	T
2507885	C	A	1	.	.	.	.	.	.	.	.	.	.	.	.	.	A

2508487	A	G	14	G	G	G	G	G	G	G	G	G	G	G	G	G	G
2509516	C	A	14	A	A	A	A	A	A	A	A	A	A	A	A	A	A
2516291	C	G	14	G	G	G	G	G	G	G	G	G	G	G	G	G	G
2520657	G	A	14	A	A	A	A	A	A	A	A	A	A	A	A	A	A
2522750	G	A	14	A	A	A	A	A	A	A	A	A	A	A	A	A	A
2524232	T	C	14	C	C	C	C	C	C	C	C	C	C	C	C	C	C
2525128	G	C	14	C	C	C	C	C	C	C	C	C	C	C	C	C	C
2526744	A	C	14	C	C	C	C	C	C	C	C	C	C	C	C	C	C
2528673	G	A	14	A	A	A	A	A	A	A	A	A	A	A	A	A	A
2570140	T	C	14	C	C	C	C	C	C	C	C	C	C	C	C	C	C
2579911	C	G	14	G	G	G	G	G	G	G	G	G	G	G	G	G	G
2581661	A	C	14	C	C	C	C	C	C	C	C	C	C	C	C	C	C
2595973	C	G	14	G	G	G	G	G	G	G	G	G	G	G	G	G	G
2598417	C	G	14	G	G	G	G	G	G	G	G	G	G	G	G	G	G
2611315	A	G	14	G	G	G	G	G	G	G	G	G	G	G	G	G	G
2612237	G	A	14	A	A	A	A	A	A	A	A	A	A	A	A	A	A
2622611	G	A	1	.	.	.	.	.	.	.	.	.	.	A	.	.	.
2623064	C	T	4	.	T	.	.	.	.	.	T	T	.	.	.	T	.
2623101	C	G	1	.	.	.	.	.	.	G	.	.	.	.	.	.	.
2640287	T	G	14	G	G	G	G	G	G	G	G	G	G	G	G	G	G
2648953	C	T	1	.	.	.	.	.	.	.	.	.	.	T	.	.	.
2652375	G	A	14	A	A	A	A	A	A	A	A	A	A	A	A	A	A
2663213	T	C	14	C	C	C	C	C	C	C	C	C	C	C	C	C	C
2673565	A	G	14	G	G	G	G	G	G	G	G	G	G	G	G	G	G
2683445	C	T	14	T	T	T	T	T	T	T	T	T	T	T	T	T	T
2685626	A	G	14	G	G	G	G	G	G	G	G	G	G	G	G	G	G
2697727	G	T	14	T	T	T	T	T	T	T	T	T	T	T	T	T	T
2719060	G	A	14	A	A	A	A	A	A	A	A	A	A	A	A	A	A
2721312	T	C	14	C	C	C	C	C	C	C	C	C	C	C	C	C	C
2727107	C	G	14	G	G	G	G	G	G	G	G	G	G	G	G	G	G

2727948	T	C	1	.	.	.	.	.	.	.	.	.	.	.	.	.	C
2729322	A	G	14	G	G	G	G	G	G	G	G	G	G	G	G	G	G
2734915	T	C	2	C	.	.	.	.	.	.	.	.	.	.	C	.	.
2736146	C	T	14	T	T	T	T	T	T	T	T	T	T	T	T	T	T
2737343	A	G	2	.	.	.	.	.	.	.	G	G	.	.	.	.	.
2743527	T	C	14	C	C	C	C	C	C	C	C	C	C	C	C	C	C
2745185	A	G	14	G	G	G	G	G	G	G	G	G	G	G	G	G	G
2751228	C	T	14	T	T	T	T	T	T	T	T	T	T	T	T	T	T
2751229	G	T	14	T	T	T	T	T	T	T	T	T	T	T	T	T	T
2754491	C	T	14	T	T	T	T	T	T	T	T	T	T	T	T	T	T
2757644	G	T	1	.	.	.	.	T	.	.	.	.	.	.	.	.	.
2761158	T	C	1	.	.	.	.	.	.	.	.	.	C	.	.	.	.
2763263	A	G	14	G	G	G	G	G	G	G	G	G	G	G	G	G	G
2763299	G	C	14	C	C	C	C	C	C	C	C	C	C	C	C	C	C
2766218	G	C	14	C	C	C	C	C	C	C	C	C	C	C	C	C	C
2768606	C	T	14	T	T	T	T	T	T	T	T	T	T	T	T	T	T
2787400	T	C	14	C	C	C	C	C	C	C	C	C	C	C	C	C	C
2789233	C	T	1	.	T	.	.	.	.	.	.	.	.	.	.	.	.
2801368	G	A	14	A	A	A	A	A	A	A	A	A	A	A	A	A	A
2802339	T	C	14	C	C	C	C	C	C	C	C	C	C	C	C	C	C
2802852	G	A	1	.	.	.	.	.	.	.	.	.	.	A	.	.	.
2803246	C	G	14	G	G	G	G	G	G	G	G	G	G	G	G	G	G
2803247	C	G	14	G	G	G	G	G	G	G	G	G	G	G	G	G	G
2804991	A	G	14	G	G	G	G	G	G	G	G	G	G	G	G	G	G
2818841	G	C	14	C	C	C	C	C	C	C	C	C	C	C	C	C	C
2819335	G	A	14	A	A	A	A	A	A	A	A	A	A	A	A	A	A
2820099	A	G	14	G	G	G	G	G	G	G	G	G	G	G	G	G	G
2824579	A	G	14	G	G	G	G	G	G	G	G	G	G	G	G	G	G
2854913	G	T	14	T	T	T	T	T	T	T	T	T	T	T	T	T	T
2856574	G	A	14	A	A	A	A	A	A	A	A	A	A	A	A	A	A

2858264	T	C	14	C	C	C	C	C	C	C	C	C	C	C	C	C	C
2859103	T	G	14	G	G	G	G	G	G	G	G	G	G	G	G	G	G
2863355	G	A	2	.	.	.	.	.	.	.	A	A	.	.	.	.	.
2863480	G	A	1	.	A	.	.	.	.	.	.	.	.	.	.	.	.
2866360	T	C	14	C	C	C	C	C	C	C	C	C	C	C	C	C	C
2866924	G	T	1	.	.	.	.	.	.	.	.	.	.	.	.	.	T
2868763	A	G	14	G	G	G	G	G	G	G	G	G	G	G	G	G	G
2871519	A	G	14	G	G	G	G	G	G	G	G	G	G	G	G	G	G
2875593	G	A	1	.	.	.	.	.	A	.	.	.	.	.	.	.	.
2877555	A	G	1	.	.	.	.	.	.	G	.	.	.	.	.	.	.
2877601	C	T	1	.	.	.	.	.	.	T	.	.	.	.	.	.	.
2881854	C	T	14	T	T	T	T	T	T	T	T	T	T	T	T	T	T
2887265	A	G	14	G	G	G	G	G	G	G	G	G	G	G	G	G	G
2888781	A	G	14	G	G	G	G	G	G	G	G	G	G	G	G	G	G
2892386	C	T	14	T	T	T	T	T	T	T	T	T	T	T	T	T	T
2895787	C	G	1	N	-	N	N	-	-	N	N	-	N	-	G	-	N
2895789	A	C	5	C	-	N	N	-	-	N	C	-	C	-	C	-	C
2895790	A	G	3	N	-	N	N	-	-	N	G	-	G	-	G	-	N
2895791	T	C	7	C	-	C	N	-	-	C	C	-	C	-	C	C	N
2896261	A	G	14	G	G	G	G	G	G	G	G	G	G	G	G	G	G
2902796	C	T	1	T	.	.	.	.	.	.	.	.	.	.	.	.	.
2911149	A	G	14	G	G	G	G	G	G	G	G	G	G	G	G	G	G
2913939	C	T	14	T	T	T	T	T	T	T	T	T	T	T	T	T	T
2920511	A	G	14	G	G	G	G	G	G	G	G	G	G	G	G	G	G
2922870	G	C	14	C	C	C	C	C	C	C	C	C	C	C	C	C	C
2929496	G	A	14	A	A	A	A	A	A	A	A	A	A	A	A	A	A
2938662	A	G	14	G	G	G	G	G	G	G	G	G	G	G	G	G	G
2940876	G	A	14	A	A	A	A	A	A	A	A	A	A	A	A	A	A
2945083	T	C	1	.	.	.	.	.	.	.	.	.	.	C	.	.	.
2950941	C	G	14	G	G	G	G	G	G	G	G	G	G	G	G	G	G

2959945	T	G	14	G	G	G	G	G	G	G	G	G	G	G	G	G	G
2979318	A	G	14	G	G	G	G	G	G	G	G	G	G	G	G	G	G
2979383	T	C	2	.	.	.	N	.	C	.	.	.	C	.	.	.	.
2979894	T	G	14	G	G	G	G	G	G	G	G	G	G	G	G	G	G
2984316	G	T	1	.	.	.	.	.	.	T	.	.	.	.	.	.	.
2987466	A	G	1	.	.	.	.	.	.	G	.	.	.	.	.	.	.
2990430	T	C	14	C	C	C	C	C	C	C	C	C	C	C	C	C	C
2990655	C	T	14	T	T	T	T	T	T	T	T	T	T	T	T	T	T
2993981	C	G	14	G	G	G	G	G	G	G	G	G	G	G	G	G	G
2995048	C	T	14	T	T	T	T	T	T	T	T	T	T	T	T	T	T
2998343	C	T	14	T	T	T	T	T	T	T	T	T	T	T	T	T	T
3001650	T	C	14	C	C	C	C	C	C	C	C	C	C	C	C	C	C
3011970	G	C	3	C	N	N	N	N	N	N	N	N	N	N	C	N	C
3011972	C	G	10	G	N	G	G	G	G	G	G	N	G	N	G	N	G
3014331	G	T	14	T	T	T	T	T	T	T	T	T	T	T	T	T	T
3018961	G	A	14	A	A	A	A	A	A	A	A	A	A	A	A	A	A
3031746	G	T	14	T	T	T	T	T	T	T	T	T	T	T	T	T	T
3033371	G	A	14	A	A	A	A	A	A	A	A	A	A	A	A	A	A
3036354	A	C	14	C	C	C	C	C	C	C	C	C	C	C	C	C	C
3036558	A	G	14	G	G	G	G	G	G	G	G	G	G	G	G	G	G
3038652	T	G	14	G	G	G	G	G	G	G	G	G	G	G	G	G	G
3045641	T	C	1	.	C	.	.	.	.	.	.	.	.	.	.	.	.
3046068	G	A	14	A	A	A	A	A	A	A	A	A	A	A	A	A	A
3049393	G	A	14	A	A	A	A	A	A	A	A	A	A	A	A	A	A
3052047	A	C	14	C	C	C	C	C	C	C	C	C	C	C	C	C	C
3058630	C	T	14	T	T	T	T	T	T	T	T	T	T	T	T	T	T
3058818	A	G	14	G	G	G	G	G	G	G	G	G	G	G	G	G	G
3063785	A	G	14	G	G	G	G	G	G	G	G	G	G	G	G	G	G
3066333	C	T	1	.	T	.	.	.	.	.	.	.	.	.	.	.	.
3066386	C	T	14	T	T	T	T	T	T	T	T	T	T	T	T	T	T

3071411	T	C	14	C	C	C	C	C	C	C	C	C	C	C	C	C	C
3076307	T	A	3	A	N	N	N	A	N	N	N	N	N	N	N	N	A
3076308	T	G	1	N	N	N	N	G	N	N	N	N	N	N	N	N	N
3079494	A	G	3	N	G	N	N	N	N	N	N	G	N	N	N	G	N
3079498	C	G	3	N	G	N	N	N	N	N	N	G	N	N	N	G	N
3089281	G	T	14	T	T	T	T	T	T	T	T	T	T	T	T	T	T
3090844	G	A	1	.	.	.	.	A	.	.	.	.	.	.	.	.	.
3092894	G	T	4	N	T	N	N	N	N	N	N	T	N	T	N	T	N
3094682	T	C	14	C	C	C	C	C	C	C	C	C	C	C	C	C	C
3095212	A	G	14	G	G	G	G	G	G	G	G	G	G	G	G	G	G
3096389	G	C	14	C	C	C	C	C	C	C	C	C	C	C	C	C	C
3096587	G	A	14	A	A	A	A	A	A	A	A	A	A	A	A	A	A
3099107	T	C	14	C	C	C	C	C	C	C	C	C	C	C	C	C	C
3099893	C	A	14	A	A	A	A	A	A	A	A	A	A	A	A	A	A
3119499	G	A	14	A	A	A	A	A	A	A	A	A	A	A	A	A	A
3120087	A	C	14	C	C	C	C	C	C	C	C	C	C	C	C	C	C
3120175	T	C	12	C	N	C	C	C	C	C	C	N	C	C	C	C	C
3120181	T	C	12	C	N	C	C	C	C	C	C	C	C	C	C	N	C
3120220	T	C	13	C	C	C	C	C	C	N	C	C	C	C	C	C	C
3120221	G	A	13	A	A	A	A	A	A	N	A	A	A	A	A	A	A
3133224	A	G	14	G	G	G	G	G	G	G	G	G	G	G	G	G	G
3136095	G	T	14	T	T	T	T	T	T	T	T	T	T	T	T	T	T
3137456	C	T	14	T	T	T	T	T	T	T	T	T	T	T	T	T	T
3140276	G	A	14	A	A	A	A	A	A	A	A	A	A	A	A	A	A
3141441	T	G	14	G	G	G	G	G	G	G	G	G	G	G	G	G	G
3144645	G	A	14	A	A	A	A	A	A	A	A	A	A	A	A	A	A
3148733	G	A	14	A	A	A	A	A	A	A	A	A	A	A	A	A	A
3149893	T	C	14	C	C	C	C	C	C	C	C	C	C	C	C	C	C
3160201	A	G	14	G	G	G	G	G	G	G	G	G	G	G	G	G	G
3164390	G	A	14	A	A	A	A	A	A	A	A	A	A	A	A	A	A

3169321	T	C	14	C	C	C	C	C	C	C	C	C	C	C	C	C	C
3178260	A	G	14	G	G	G	G	G	G	G	G	G	G	G	G	G	G
3182568	C	T	6	.	T	T	N	T	.	.	T	T	.	.	.	T	.
3186694	C	A	14	A	A	A	A	A	A	A	A	A	A	A	A	A	A
3187545	A	G	14	G	G	G	G	G	G	G	G	G	G	G	G	G	G
3189384	A	G	13	G	G	G	G	G	G	G	G	N	G	G	G	G	G
3189440	A	G	2	N	G	N	N	N	N	N	N	G	N	N	N	N	N
3210270	G	A	14	A	A	A	A	A	A	A	A	A	A	A	A	A	A
3214426	G	A	1	.	.	.	.	.	.	.	.	.	.	A	.	.	.
3239805	G	T	1	.	.	.	N	.	T	.	.	.	.	.	.	.	.
3240077	T	G	14	G	G	G	G	G	G	G	G	G	G	G	G	G	G
3243304	T	C	14	C	C	C	C	C	C	C	C	C	C	C	C	C	C
3263664	C	T	14	T	T	T	T	T	T	T	T	T	T	T	T	T	T
3265937	A	G	14	G	G	G	G	G	G	G	G	G	G	G	G	G	G
3286787	T	G	14	G	G	G	G	G	G	G	G	G	G	G	G	G	G
3300139	C	T	14	T	T	T	T	T	T	T	T	T	T	T	T	T	T
3303798	T	G	14	G	G	G	G	G	G	G	G	G	G	G	G	G	G
3305747	A	G	14	G	G	G	G	G	G	G	G	G	G	G	G	G	G
3317768	G	A	3	.	A	.	.	.	.	.	A	A	.	.	.	.	.
3334057	C	G	14	G	G	G	G	G	G	G	G	G	G	G	G	G	G
3343629	C	T	14	T	T	T	T	T	T	T	T	T	T	T	T	T	T
3343634	G	A	14	A	A	A	A	A	A	A	A	A	A	A	A	A	A
3349403	A	C	14	C	C	C	C	C	C	C	C	C	C	C	C	C	C
3353685	C	T	14	T	T	T	T	T	T	T	T	T	T	T	T	T	T
3366915	A	G	14	G	G	G	G	G	G	G	G	G	G	G	G	G	G
3376338	A	G	14	G	G	G	G	G	G	G	G	G	G	G	G	G	G
3380659	T	A	14	A	A	A	A	A	A	A	A	A	A	A	A	A	A
3380686	T	C	14	C	C	C	C	C	C	C	C	C	C	C	C	C	C
3382742	G	T	14	T	T	T	T	T	T	T	T	T	T	T	T	T	T
3388155	A	G	14	G	G	G	G	G	G	G	G	G	G	G	G	G	G

3392383	G	C	14	C	C	C	C	C	C	C	C	C	C	C	C	C	C
3403083	T	C	14	C	C	C	C	C	C	C	C	C	C	C	C	C	C
3405466	T	G	14	G	G	G	G	G	G	G	G	G	G	G	G	G	G
3407543	A	G	14	G	G	G	G	G	G	G	G	G	G	G	G	G	G
3410117	G	A	14	A	A	A	A	A	A	A	A	A	A	A	A	A	A
3416905	G	A	1	.	.	.	.	.	.	.	.	.	A	.	.	.	.
3417745	C	T	14	T	T	T	T	T	T	T	T	T	T	T	T	T	T
3422724	A	G	14	G	G	G	G	G	G	G	G	G	G	G	G	G	G
3433626	G	A	1	.	.	.	.	.	.	.	.	.	A	.	.	.	.
3434211	G	A	14	A	A	A	A	A	A	A	A	A	A	A	A	A	A
3435340	G	A	14	A	A	A	A	A	A	A	A	A	A	A	A	A	A
3443927	A	G	14	G	G	G	G	G	G	G	G	G	G	G	G	G	G
3444781	T	C	4	C	-	-	-	-	-	C	-	-	C	-	C	-	-
3445557	G	A	1	.	.	.	N	.	A	.	.	.	.	.	.	.	.
3447631	C	A	14	A	A	A	A	A	A	A	A	A	A	A	A	A	A
3455216	G	A	1	.	.	.	N	.	A	.	.	.	.	.	.	.	.
3455277	G	A	1	.	.	A	.	.	.	.	.	.	.	.	.	.	.
3455916	G	A	9	.	A	A	A	A	A	.	A	A	A	.	.	A	.
3455977	T	C	1	.	.	.	.	.	.	.	.	.	.	.	.	C	.
3456046	A	G	1	.	.	.	.	.	.	.	.	.	.	.	.	.	G
3457066	G	T	14	T	T	T	T	T	T	T	T	T	T	T	T	T	T
3460247	C	T	14	T	T	T	T	T	T	T	T	T	T	T	T	T	T
3460286	T	C	14	C	C	C	C	C	C	C	C	C	C	C	C	C	C
3464431	C	A	14	A	A	A	A	A	A	A	A	A	A	A	A	A	A
3467365	C	A	14	A	A	A	A	A	A	A	A	A	A	A	A	A	A
3467994	C	T	1	.	T	.	.	.	.	.	.	.	.	.	.	.	.
3472661	G	A	14	A	A	A	A	A	A	A	A	A	A	A	A	A	A
3478406	A	T	14	T	T	T	T	T	T	T	T	T	T	T	T	T	T
3480598	A	G	14	G	G	G	G	G	G	G	G	G	G	G	G	G	G
3482269	T	A	14	A	A	A	A	A	A	A	A	A	A	A	A	A	A

3484184	G	A	8	N	A	A	A	N	N	N	A	A	N	A	N	A	A
3489322	T	G	14	G	G	G	G	G	G	G	G	G	G	G	G	G	G
3491278	C	T	14	T	T	T	T	T	T	T	T	T	T	T	T	T	T
3491495	G	A	2	A	.	.	.	.	.	.	.	.	.	.	A	.	.
3492179	A	C	14	C	C	C	C	C	C	C	C	C	C	C	C	C	C
3493725	C	T	14	T	T	T	T	T	T	T	T	T	T	T	T	T	T
3513492	G	T	14	T	T	T	T	T	T	T	T	T	T	T	T	T	T
3516822	G	C	14	C	C	C	C	C	C	C	C	C	C	C	C	C	C
3519089	G	T	2	.	.	T	.	T	.	.	.	.	.	.	.	.	.
3522719	G	A	1	A	.	.	.	.	.	.	.	.	.	.	.	.	.
3523580	C	T	1	.	.	.	.	T	.	.	.	.	.	.	.	.	.
3542571	C	T	14	T	T	T	T	T	T	T	T	T	T	T	T	T	T
3544791	A	G	14	G	G	G	G	G	G	G	G	G	G	G	G	G	G
3545360	C	G	4	-	-	-	G	-	N	G	-	-	G	-	G	-	-
3545362	A	C	5	N	C	N	N	N	N	N	C	C	N	C	N	C	N
3545363	G	A	11	A	A	A	N	A	A	A	A	A	N	A	N	A	A
3553036	C	T	1	T	.	.	.	.	.	.	.	.	.	.	.	.	.
3554474	G	T	14	T	T	T	T	T	T	T	T	T	T	T	T	T	T
3558607	A	G	14	G	G	G	G	G	G	G	G	G	G	G	G	G	G
3563308	A	G	14	G	G	G	G	G	G	G	G	G	G	G	G	G	G
3565019	C	A	1	.	.	.	.	.	.	.	.	.	.	.	.	.	A
3569787	T	C	14	C	C	C	C	C	C	C	C	C	C	C	C	C	C
3576729	A	G	14	G	G	G	G	G	G	G	G	G	G	G	G	G	G
3578155	G	C	14	C	C	C	C	C	C	C	C	C	C	C	C	C	C
3578761	G	T	1	.	.	.	.	.	.	.	.	.	.	T	.	.	.
3583866	T	C	14	C	C	C	C	C	C	C	C	C	C	C	C	C	C
3595234	T	C	1	.	.	.	.	.	.	C	.	.	.	.	.	.	.
3608842	C	T	1	.	.	.	N	.	T	.	.	.	.	.	.	.	.
3608993	G	A	14	A	A	A	A	A	A	A	A	A	A	A	A	A	A
3609328	C	T	2	T	.	.	.	.	.	.	.	.	.	.	T	.	.

3615780	A	G	1	.	.	.	.	.	.	.	.	.	.	.	.	.	G
3620144	A	G	14	G	G	G	G	G	G	G	G	G	G	G	G	G	G
3621901	T	C	14	C	C	C	C	C	C	C	C	C	C	C	C	C	C
3641455	T	C	14	C	C	C	C	C	C	C	C	C	C	C	C	C	C
3642066	C	A	14	A	A	A	A	A	A	A	A	A	A	A	A	A	A
3662284	C	G	14	G	G	G	G	G	G	G	G	G	G	G	G	G	G
3662285	G	C	14	C	C	C	C	C	C	C	C	C	C	C	C	C	C
3663602	A	G	14	G	G	G	G	G	G	G	G	G	G	G	G	G	G
3673340	A	G	14	G	G	G	G	G	G	G	G	G	G	G	G	G	G
3679785	G	A	14	A	A	A	A	A	A	A	A	A	A	A	A	A	A
3682694	G	A	4	.	A	.	.	.	.	.	A	A	.	.	.	A	.
3683816	C	G	14	G	G	G	G	G	G	G	G	G	G	G	G	G	G
3688084	A	G	3	N	N	N	N	N	N	N	N	G	N	G	N	G	N
3688090	A	G	1	N	N	N	N	N	N	N	N	N	N	G	N	N	N
3689285	A	G	14	G	G	G	G	G	G	G	G	G	G	G	G	G	G
3692429	C	T	1	N	.	.	.	.	.	N	.	.	T	.	.	.	.
3696575	C	G	2	G	.	.	.	.	.	.	.	.	.	.	G	.	.
3703648	A	G	14	G	G	G	G	G	G	G	G	G	G	G	G	G	G
3705212	T	C	14	C	C	C	C	C	C	C	C	C	C	C	C	C	C
3710266	A	G	1	.	.	.	.	.	G	.	.	.	.	.	.	.	.
3715584	A	G	14	G	G	G	G	G	G	G	G	G	G	G	G	G	G
3719336	C	G	14	G	G	G	G	G	G	G	G	G	G	G	G	G	G
3720988	G	A	14	A	A	A	A	A	A	A	A	A	A	A	A	A	A
3725136	G	T	14	T	T	T	T	T	T	T	T	T	T	T	T	T	T
3728534	G	A	14	A	A	A	A	A	A	A	A	A	A	A	A	A	A
3736999	T	C	14	C	C	C	C	C	C	C	C	C	C	C	C	C	C
3746676	A	G	14	G	G	G	G	G	G	G	G	G	G	G	G	G	G
3747150	C	T	14	T	T	T	T	T	T	T	T	T	T	T	T	T	T
3756815	A	G	14	G	G	G	G	G	G	G	G	G	G	G	G	G	G
3759091	G	A	14	A	A	A	A	A	A	A	A	A	A	A	A	A	A

3772552	A	G	13	G	G	G	G	G	G	N	G	G	G	G	G	G	G
3772828	T	G	4	G	.	.	N	.	.	N	.	.	.	G	G	.	G
3772945	A	G	13	G	G	G	G	G	G	N	G	G	G	G	G	G	G
3774270	A	G	13	G	G	G	G	G	G	N	G	G	G	G	G	G	G
3776720	A	G	1	.	.	.	.	.	.	N	.	.	.	.	.	G	.
3782003	C	T	1	.	.	T	.	.	.	.	.	.	.	.	.	.	.
3787664	A	T	2	T	.	.	.	.	.	.	.	.	.	.	T	.	.
3787772	A	T	2	T	.	.	.	.	.	.	.	.	.	.	T	.	.
3796023	T	C	14	C	C	C	C	C	C	C	C	C	C	C	C	C	C
3799479	G	A	2	.	.	A	.	A	.	.	.	.	.	.	.	.	.
3801019	T	C	1	.	.	.	.	.	.	.	.	.	.	C	.	.	.
3801267	T	C	14	C	C	C	C	C	C	C	C	C	C	C	C	C	C
3812678	T	C	14	C	C	C	C	C	C	C	C	C	C	C	C	C	C
3816169	C	T	14	T	T	T	T	T	T	T	T	T	T	T	T	T	T
3817059	A	C	14	C	C	C	C	C	C	C	C	C	C	C	C	C	C
3823299	A	G	14	G	G	G	G	G	G	G	G	G	G	G	G	G	G
3825621	T	C	2	C	.	.	.	.	.	.	.	.	.	.	C	.	.
3826230	G	A	1	.	.	.	.	A	.	.	.	.	.	.	.	.	.
3834238	T	C	14	C	C	C	C	C	C	C	C	C	C	C	C	C	C
3835453	T	C	14	C	C	C	C	C	C	C	C	C	C	C	C	C	C
3836500	C	G	2	G	.	.	.	.	.	.	.	.	.	.	G	.	.
3836789	C	G	14	G	G	G	G	G	G	G	G	G	G	G	G	G	G
3840723	T	C	14	C	C	C	C	C	C	C	C	C	C	C	C	C	C
3842373	A	G	14	G	G	G	G	G	G	G	G	G	G	G	G	G	G
3845632	C	T	14	T	T	T	T	T	T	T	T	T	T	T	T	T	T
3853341	G	A	14	A	A	A	A	A	A	A	A	A	A	A	A	A	A
3856306	G	A	1	A	.	.	.	.	.	.	.	.	.	.	.	.	.
3862669	T	G	14	G	G	G	G	G	G	G	G	G	G	G	G	G	G
3864176	G	A	14	A	A	A	A	A	A	A	A	A	A	A	A	A	A
3867352	G	C	14	C	C	C	C	C	C	C	C	C	C	C	C	C	C

3868910	G	A	1	.	.	.	.	.	.	.	.	.	.	A	.	.	.
3870244	A	G	14	G	G	G	G	G	G	G	G	G	G	G	G	G	G
3874032	C	A	1	A	.	N	N	N	N	N	N	.	N	.	N	.	N
3877066	G	A	2	N	N	N	N	N	N	N	A	N	N	N	N	N	A
3878672	C	G	1	N	G	N	N	N	N	N	N	N	N	N	N	N	N
3878729	C	G	2	N	N	N	N	N	G	N	N	N	N	N	G	N	N
3878730	C	G	2	N	N	N	N	N	G	N	N	N	N	N	G	N	N
3878733	A	C	6	C	N	C	N	C	C	N	N	N	N	N	C	N	C
3878736	C	T	5	N	N	T	N	T	T	N	N	N	N	N	T	N	T
3878959	A	G	6	-	-	G	G	G	G	N	N	-	-	-	G	-	G
3878960	A	G	5	-	-	G	G	G	G	N	N	-	-	-	G	-	N
3878961	C	A	3	-	-	N	N	A	A	N	N	-	-	-	A	-	N
3878964	C	T	1	-	-	N	N	T	N	N	N	-	-	-	N	-	N
3878965	T	G	5	-	-	G	G	G	G	N	G	-	-	-	N	-	N
3879809	C	G	3	N	N	N	N	N	N	N	G	N	N	G	N	N	G
3880106	A	G	6	-	G	-	-	-	-	G	G	G	-	G	-	G	-
3880331	C	T	13	T	T	T	T	T	T	N	T	T	T	T	T	T	T
3889139	G	C	2	N	N	C	N	N	N	N	N	N	C	N	N	N	N
3889141	C	G	3	N	N	G	N	N	N	N	N	N	G	G	N	N	N
3889254	A	G	8	N	N	G	N	G	N	N	G	G	G	N	G	G	G
3889256	T	C	9	N	N	C	C	C	N	N	C	C	C	N	C	C	C
3889410	G	A	7	N	A	N	N	A	N	N	N	A	A	A	N	A	A
3889411	G	C	6	N	C	N	N	C	N	N	N	N	C	C	N	C	C
3889423	C	A	6	A	N	N	A	A	N	N	N	N	N	N	A	A	A
3889424	C	G	1	N	N	N	N	G	N	N	N	N	N	N	N	N	N
3889427	C	T	1	N	N	N	N	T	N	N	N	N	N	N	N	N	N
3889637	T	C	1	C	N	N	N	N	N	N	N	N	N	N	N	N	N
3889640	T	G	1	G	N	N	N	N	N	N	N	N	N	N	N	N	N
3889642	C	A	1	A	N	N	N	N	N	N	N	N	N	N	N	N	N
3889643	T	C	3	C	N	C	N	C	N	N	N	N	N	N	N	N	N

3889646	C	G	8	G	G	G	N	G	N	N	N	G	N	G	G	G	N
3891002	G	A	3	N	N	N	A	N	N	N	A	N	N	N	N	N	A
3892351	A	G	1	N	G	N	N	N	N	N	N	N	N	N	N	N	N
3902325	A	G	14	G	G	G	G	G	G	G	G	G	G	G	G	G	G
3905771	G	A	1	.	.	.	.	.	.	.	.	.	.	.	.	.	A
3909846	C	T	14	T	T	T	T	T	T	T	T	T	T	T	T	T	T
3932547	C	T	14	T	T	T	T	T	T	T	T	T	T	T	T	T	T
3953110	C	T	14	T	T	T	T	T	T	T	T	T	T	T	T	T	T
3953716	G	A	1	.	.	.	.	.	.	.	.	.	A	.	.	.	.
3965303	T	C	14	C	C	C	C	C	C	C	C	C	C	C	C	C	C
3968207	C	T	14	T	T	T	T	T	T	T	T	T	T	T	T	T	T
3968948	T	C	1	.	.	.	.	.	.	.	.	.	.	.	.	.	C
3973699	G	A	14	A	A	A	A	A	A	A	A	A	A	A	A	A	A
3978421	A	G	14	G	G	G	G	G	G	G	G	G	G	G	G	G	G
3978921	T	C	1	.	.	.	.	.	.	.	.	.	.	.	.	C	.
3984600	C	G	14	G	G	G	G	G	G	G	G	G	G	G	G	G	G
3991459	A	G	14	G	G	G	G	G	G	G	G	G	G	G	G	G	G
3992000	G	C	14	C	C	C	C	C	C	C	C	C	C	C	C	C	C
3999273	G	A	14	A	A	A	A	A	A	A	A	A	A	A	A	A	A
3999720	G	A	14	A	A	A	A	A	A	A	A	A	A	A	A	A	A
3999788	G	A	14	A	A	A	A	A	A	A	A	A	A	A	A	A	A
4001003	C	T	8	.	T	T	N	T	T	.	T	T	T	.	.	T	.
4001036	T	C	14	C	C	C	C	C	C	C	C	C	C	C	C	C	C
4006573	T	C	14	C	C	C	C	C	C	C	C	C	C	C	C	C	C
4014113	T	C	14	C	C	C	C	C	C	C	C	C	C	C	C	C	C
4022838	G	A	14	A	A	A	A	A	A	A	A	A	A	A	A	A	A
4026017	G	C	4	.	C	.	.	.	.	.	C	C	.	.	.	C	.
4028272	G	A	14	A	A	A	A	A	A	A	A	A	A	A	A	A	A
4028718	G	A	14	A	A	A	A	A	A	A	A	A	A	A	A	A	A
4031931	C	A	2	-	A	-	-	-	-	N	-	A	-	-	-	-	-

4034157	A	G	14	G	G	G	G	G	G	G	G	G	G	G	G	G	G
4043305	C	T	14	T	T	T	T	T	T	T	T	T	T	T	T	T	T
4044976	A	T	2	.	.	.	.	.	.	.	T	T	.	.	.	.	.
4045545	A	G	14	G	G	G	G	G	G	G	G	G	G	G	G	G	G
4050013	A	G	14	G	G	G	G	G	G	G	G	G	G	G	G	G	G
4050594	T	G	14	G	G	G	G	G	G	G	G	G	G	G	G	G	G
4053973	G	A	1	.	.	.	.	.	.	.	.	.	.	.	.	.	A
4054189	T	C	8	.	C	C	N	C	C	.	C	C	C	.	.	C	.
4054581	G	A	14	A	A	A	A	A	A	A	A	A	A	A	A	A	A
4054600	T	C	1	.	.	.	.	.	.	.	.	.	.	.	.	.	C
4060241	G	A	2	A	.	.	.	.	.	.	.	.	.	.	A	.	.
4073002	T	C	14	C	C	C	C	C	C	C	C	C	C	C	C	C	C
4076549	G	C	14	C	C	C	C	C	C	C	C	C	C	C	C	C	C
4076597	A	G	1	G	.	.	.	.	.	.	.	.	.	.	.	.	.
4077818	A	G	8	.	G	G	N	G	G	.	G	G	G	N	.	G	.
4078071	C	A	13	A	A	A	A	A	A	A	A	A	A	N	A	A	A
4078928	T	C	13	C	C	C	C	C	C	C	C	C	C	N	C	C	C
4089290	C	T	14	T	T	T	T	T	T	T	T	T	T	T	T	T	T
4092425	T	G	14	G	G	G	G	G	G	G	G	G	G	G	G	G	G
4094594	T	G	1	G	N	N	N	N	N	N	N	N	N	N	N	N	N
4094595	G	T	2	T	N	N	N	N	N	T	N	N	N	N	N	N	N
4094598	T	A	1	N	N	N	N	N	N	A	N	N	N	N	N	N	N
4094601	A	G	1	N	N	N	N	N	N	G	N	N	N	N	N	N	N
4098534	A	C	1	C	.	.	.	.	.	.	.	.	.	.	.	.	.
4104874	G	A	14	A	A	A	A	A	A	A	A	A	A	A	A	A	A
4115902	G	A	1	.	.	.	.	.	.	.	.	.	.	.	.	A	.
4124550	G	A	14	A	A	A	A	A	A	A	A	A	A	A	A	A	A
4126746	A	C	14	C	C	C	C	C	C	C	C	C	C	C	C	C	C
4144920	C	A	14	A	A	A	A	A	A	A	A	A	A	A	A	A	A
4158373	T	C	14	C	C	C	C	C	C	C	C	C	C	C	C	C	C

4159498	C	G	1	.	.	.	.	.	.	.	.	.	.	.	.	G	.
4174869	C	A	14	A	A	A	A	A	A	A	A	A	A	A	A	A	A
4176805	A	G	14	G	G	G	G	G	G	G	G	G	G	G	G	G	G
4178555	C	T	14	T	T	T	T	T	T	T	T	T	T	T	T	T	T
4183699	G	A	14	A	A	A	A	A	A	A	A	A	A	A	A	A	A
4195702	C	T	14	T	T	T	T	T	T	T	T	T	T	T	T	T	T
4199010	G	A	1	.	A	.	.	.	.	.	.	.	.	.	.	.	.
4199457	C	T	14	T	T	T	T	T	T	T	T	T	T	T	T	T	T
4206193	C	T	14	T	T	T	T	T	T	T	T	T	T	T	T	T	T
4210078	G	T	14	T	T	T	T	T	T	T	T	T	T	T	T	T	T
4214828	C	G	14	G	G	G	G	G	G	G	G	G	G	G	G	G	G
4218015	A	G	14	G	G	G	G	G	G	G	G	G	G	G	G	G	G
4230761	T	C	14	C	C	C	C	C	C	C	C	C	C	C	C	C	C
4233624	A	C	14	C	C	C	C	C	C	C	C	C	C	C	C	C	C
4237937	T	C	1	.	.	.	.	.	.	.	.	.	.	.	.	.	C
4240019	C	T	14	T	T	T	T	T	T	T	T	T	T	T	T	T	T
4241581	A	G	14	G	G	G	G	G	G	G	G	G	G	G	G	G	G
4242040	A	G	1	.	.	.	.	.	.	.	.	.	G	.	.	.	.
4245785	A	T	14	T	T	T	T	T	T	T	T	T	T	T	T	T	T
4266769	T	G	14	G	G	G	G	G	G	G	G	G	G	G	G	G	G
4266883	G	T	14	T	T	T	T	T	T	T	T	T	T	T	T	T	T
4272335	C	T	1	.	.	.	.	.	T	.	.	.	.	.	.	.	.
4296321	C	T	1	N	N	N	N	N	N	N	N	N	N	N	N	N	T
4307247	C	T	13	T	T	T	T	T	T	N	T	T	T	T	T	T	T
4309257	C	T	14	T	T	T	T	T	T	T	T	T	T	T	T	T	T
4313106	G	A	14	A	A	A	A	A	A	A	A	A	A	A	A	A	A
4317124	A	C	14	C	C	C	C	C	C	C	C	C	C	C	C	C	C
4317159	T	C	14	C	C	C	C	C	C	C	C	C	C	C	C	C	C
4317642	G	A	14	A	A	A	A	A	A	A	A	A	A	A	A	A	A
4318623	C	T	2	T	.	.	.	.	.	.	.	.	.	.	T	.	.

4328460	C	G	14	G	G	G	G	G	G	G	G	G	G	G	G	G	G
4334858	T	G	14	G	G	G	G	G	G	G	G	G	G	G	G	G	G
4335342	C	T	4	.	T	.	.	.	.	.	T	T	.	.	.	T	.
4337394	T	C	14	C	C	C	C	C	C	C	C	C	C	C	C	C	C
4337936	C	T	1	.	T	.	.	.	.	.	.	.	.	.	.	.	.
4343713	G	A	14	A	A	A	A	A	A	A	A	A	A	A	A	A	A

Table S3: Sequences insertion in 14 BCG strains compared to *M. bovis*.

Start	Length (bp)	BCG strains affected
402643	202	Russia
474929	129	Glaxo
474933	200	connaught
476919	150	Glaxo
853780	1360	Moreau, Russia
965530	122	Birkhaug, Copenhagen, Glaxo, Frappier, Russia
965530	242	China, Tice
965531	122	Phipps
1278507	86	Moreau
1278508	162	Copenhagen
1306868	64	all
1341811	74	Sweden
1640995	55	Chia, Phipps, Russia, Tice
1754246	808	all (except Sweden)
1982058	251	Birkahug
2018699	157	Tokyo
2320081	211	Phipps
2322501	138	Birkahug
2655206	110	Tokyo
2833553	44	Birkahug, Connaught, Copenhagen, Glaxo
2834209	44	Frappier, Moreau
3076145	40	Birkahug, Connaught, Copenhagen, Moreau, Phipps, Russia
3079145	377	all (except Russia)
3088862	202	Sweden
3089947	81	Sweden
3089960	69	Pasteur
3092359	52	Pasteur
3112453	110	Russia
3196289	861	Tokyo
3247973	59	Russia
3293191	61	Russia
3293275	120	China
3293275	61	Phipps, Tice
3379796	70	all
3545445	94	Phipps
3561165	165	Sweden
3706540	345	Birkahug
4092808	173	Sweden
4092808	116	Tokyo
4306967	1949	Moreau
4307385	2407	all (except Moreau)

Table S4:Distribution of regions of difference and deleted genes in the genomes of 14 BCG strains compared to M. bovis.

Deleted sequence	M. bovis	Birkhaug	China	Connaught	Mexico	Tice	Russia
RD01	Mb3901 to Mb3909c	deleted	deleted	deleted	deleted	deleted	deleted
RD02	Mb2000 to Mb2010	Present	Deleted	Deleted	Deleted	Deleted	Present
RD03	Mb1599 to Mb1612c	Deleted	Deleted	Deleted	Deleted	Deleted	Deleted
RD08	Mb0317 to Mb0320	Present	Present	Deleted	Present	Present	Present
RD14	Mb1795 to Mb1802c	Present	Present	Present	Present	Present	Present
RD16	Mb3433 to Mb3439c	Present	Mb3435 deleted	Mb3435 deleted	Mb3435 deleted	Present	Present
nRD18	Mb1221 to Mb1223	Present	Deleted	Deleted	Deleted	Deleted	Present
RDDenmark/Glaxo	Mb1840 to Mb1841	Present	Present	Present	Present	Present	Present
RDFrappier	Mb3525c to Mb3527c	Present	Present	Present	Present	Present	Present
RDRussia	Mb3723c to Mb3724	Present	Present	Present	Present	Present	Deleted
RDJapan	Mb3439c	Present	Present	Present	Present	Present	Present
fadD26, ppsA	Mb2955 to Mb2956	Present	Present	Present	Present	Present	Present
RDMoreauRv3887c	Mb3917c	Present	CDS larger and insertion	Insertion	Insertion	Insertion	Insertion
trcR	Mb1062c	2446bp del	Present	Present	Present	Present	Present
whiB3	Mb3450	110bp del	Present	Present	Present	Present	Present
PhoP	Mb0780	Present	Present	Present	Present	Present	Insertion
PhoR	Mb0781	11bp del	Present	Present	Present	Present	Present
Rv0094c-95c	Mb0096c to Mb0097c	867bp del+smaller indels	Present	Present	Present	Rv0093c pseudogene	Present
pks12	pks12	440bp del	Present	2102bp insertion	Present	Present	525bp del
leuA	leuA	915bp ins	1560bp ins	118bp ins	693bp ins	1998bp ins	1517bp ins
Rv3712	Mb3739	118bp del	118bp del	118bp del	118bp del	118bp del	118bp del
frdB	frdB						
RDMexico	Mb3890 to Mb3892c	Present	Present	Present	Deleted	Present	Present
	Mb2377c	Deletion	Deletion	Deletion	Deletion	Deletion	Deletion

Danish	Frappier	Glaxo	Moreau	Phipps	Prague	Sweden	Japan	Pasteur
deleted	deleted	deleted	deleted	deleted	deleted	deleted	deleted	deleted
Deleted	Deleted	Deleted	Present	Deleted	Deleted	Present	Present	Deleted
Deleted	Deleted	Deleted	Deleted	Deleted	Deleted	Deleted	Deleted	Deleted
Present	Deleted	Present	Present	Present	Present	Present	Present	Present
Present	Present	Present	Present	Deleted	Present	Present	Present	Deleted
Present	Mb3435 deleted	Mb3435 deleted	Deleted	Present	Present	Present	Present	Present
Present	Deleted	Present	Present	Deleted	Present	Present	Present	Deleted
Deleted	Present	Mb1840 deleted	Present	Present	Present	Present	Present	Present
Present	Deleted	Present	Present	Present	Present	Present	Present	Present
Present	Present	Present	Present	Present	Present	Present	Present	Present
Present	Present	Present	Present	Present	Present	Present	22bp del	Present
Rearrangement	Present	Present	Both truncated	Present	Present	Present	Truncated	Present
Insertion	Insertion	Insertion	Indel	Ins in Mb3919c	Insertion	Insertion	Insertion	Insertion
Present	Present	Present	Present	Present	Present	2446bp del	Present	Present
Present	Present	Present	Present	Present	Present	110bp del	Present	Present
Present	Present	Present	Ins6110	Present	InsG at Rv852,068-pseudogene	Present	Insertion	Present
10bp del	1bp del Rv 852702	10bp del	Present	Present	Present	11bp del	Present	Present
894bp del	Present	Present	Deleted	Present	Present	867bp del	Present	Present
Present	2 dels-582bp	Present	Present	Present	Present	2618bp del	Present	Present
118bp ins	Present	61bp ins	180bp ins	2009bp ins	1067bp ins	372bp ins	636bp ins	693bp ins
118bp del	118bp del	118bp del	118bp del	118bp del	118bp del	118bp del	118bp del	118bp del
		5bp Ins						
Present	Present	Present	Present	Present	Present	Present	Present	Present
Deletion	Deletion	Deletion	Deletion	Deletion	Deletion	Deletion	Deletion	Deletion

Table S5: The lengths and positions of leuA gene insertions in M. bovis and BCG strains.

**Strain**	**Length of insertion in *leuA* (bp)**
M. bovis	202
Birkhaug	915
China	1560
Connaught	118
Mexico	693
Tice	1998
Russia	1517
Danish	118
Frappier	Same as H37Rv
Glaxo	118
Moreau	180
Phipps	2009
Prague	1067
Sweden	372
Japan	636
Pasteur	693

Table S6: Pearson correlation coefficient between biological replicates of RNA-seq transcriptome data.

V1	Mbovis_1	Mbovis_2	Mbovis_3	Glaxo_2	Glaxo_3	Russia_1	Russia_2	Sweden_1	Sweden_2	Sweden_3	Moreau_2	Moreau_3	China_1	China_2	China_3	Pasteur_2	Pasteur_3	Tokyo_1	Tokyo_2	Tokyo_3	Tice_1	Tice_2	Copenhagen_1	Copenhagen_2	Copenhagen_3	Phipps_1	Phipps_2	Phipps_3	Connaught_2	Connaught_3	Prague_1	Prague_3	Birkhaug_1	Birkhaug_2	Birkhaug_3	Frappier_1	Frappier_2	Frappier_3
Mbovis_1	1.00	0.97	0.99	0.62	0.60	0.66	0.64	0.68	0.69	0.70	0.63	0.63	0.47	0.47	0.47	0.55	0.55	0.67	0.68	0.68	0.62	0.63	0.68	0.62	0.63	0.49	0.49	0.49	0.59	0.58	0.58	0.61	0.69	0.68	0.67	0.60	0.58	0.61
Mbovis_2	0.97	1.00	0.98	0.60	0.58	0.63	0.61	0.67	0.67	0.68	0.67	0.66	0.50	0.51	0.50	0.55	0.55	0.68	0.68	0.69	0.62	0.62	0.66	0.60	0.61	0.52	0.52	0.52	0.62	0.62	0.54	0.58	0.69	0.69	0.68	0.62	0.62	0.63
Mbovis_3	0.99	0.98	1.00	0.62	0.60	0.66	0.64	0.67	0.68	0.69	0.63	0.63	0.46	0.46	0.46	0.53	0.53	0.67	0.67	0.68	0.61	0.62	0.67	0.62	0.62	0.47	0.47	0.47	0.59	0.58	0.58	0.60	0.68	0.68	0.67	0.60	0.58	0.62
Glaxo_2	0.62	0.60	0.62	1.00	0.99	0.91	0.91	0.90	0.90	0.90	0.82	0.82	0.66	0.66	0.66	0.70	0.71	0.89	0.90	0.89	0.94	0.95	0.97	0.98	0.98	0.71	0.72	0.72	0.89	0.88	0.97	0.97	0.89	0.89	0.89	0.90	0.86	0.92
Glaxo_3	0.60	0.58	0.60	0.99	1.00	0.90	0.90	0.88	0.89	0.88	0.78	0.78	0.64	0.64	0.64	0.68	0.70	0.87	0.88	0.87	0.93	0.94	0.97	0.98	0.98	0.69	0.70	0.70	0.87	0.86	0.98	0.97	0.87	0.87	0.86	0.88	0.83	0.91
Russia_1	0.66	0.63	0.66	0.91	0.90	1.00	0.99	0.95	0.97	0.96	0.84	0.84	0.57	0.57	0.57	0.62	0.64	0.95	0.95	0.94	0.86	0.86	0.89	0.90	0.89	0.63	0.63	0.63	0.79	0.78	0.90	0.89	0.94	0.93	0.93	0.81	0.75	0.83
Russia_2	0.64	0.61	0.64	0.91	0.90	0.99	1.00	0.94	0.96	0.94	0.82	0.81	0.54	0.54	0.54	0.59	0.61	0.93	0.94	0.92	0.84	0.85	0.88	0.89	0.88	0.60	0.60	0.60	0.77	0.75	0.90	0.89	0.92	0.92	0.91	0.78	0.72	0.81
Sweden_1	0.68	0.67	0.67	0.90	0.88	0.95	0.94	1.00	0.99	0.99	0.91	0.90	0.70	0.70	0.70	0.74	0.75	0.97	0.97	0.96	0.90	0.89	0.90	0.89	0.89	0.74	0.74	0.74	0.86	0.85	0.86	0.89	0.98	0.98	0.97	0.85	0.81	0.85
Sweden_2	0.69	0.67	0.68	0.90	0.89	0.97	0.96	0.99	1.00	0.99	0.89	0.89	0.66	0.66	0.66	0.71	0.72	0.96	0.97	0.96	0.89	0.89	0.90	0.90	0.89	0.71	0.71	0.71	0.84	0.83	0.88	0.90	0.97	0.97	0.97	0.84	0.79	0.84
Sweden_3	0.70	0.68	0.69	0.90	0.88	0.96	0.94	0.99	0.99	1.00	0.90	0.90	0.69	0.69	0.69	0.73	0.74	0.97	0.97	0.96	0.90	0.89	0.90	0.90	0.89	0.73	0.73	0.73	0.85	0.84	0.87	0.90	0.98	0.98	0.97	0.85	0.81	0.85
Moreau_2	0.63	0.67	0.63	0.82	0.78	0.84	0.82	0.91	0.89	0.90	1.00	1.00	0.77	0.78	0.77	0.75	0.75	0.93	0.92	0.94	0.87	0.85	0.82	0.81	0.81	0.80	0.80	0.80	0.88	0.88	0.75	0.79	0.93	0.93	0.93	0.88	0.88	0.86
Moreau_3	0.63	0.66	0.63	0.82	0.78	0.84	0.81	0.90	0.89	0.90	1.00	1.00	0.77	0.77	0.77	0.75	0.75	0.93	0.92	0.93	0.87	0.85	0.82	0.81	0.81	0.80	0.80	0.80	0.88	0.88	0.75	0.79	0.92	0.93	0.93	0.87	0.88	0.86
China_1	0.47	0.50	0.46	0.66	0.64	0.57	0.54	0.70	0.66	0.69	0.77	0.77	1.00	1.00	1.00	0.91	0.91	0.69	0.68	0.70	0.80	0.77	0.71	0.68	0.69	0.94	0.94	0.94	0.82	0.83	0.61	0.68	0.72	0.73	0.73	0.80	0.82	0.76
China_2	0.47	0.51	0.46	0.66	0.64	0.57	0.54	0.70	0.66	0.69	0.78	0.77	1.00	1.00	1.00	0.91	0.91	0.70	0.69	0.70	0.80	0.77	0.71	0.68	0.69	0.94	0.94	0.94	0.82	0.83	0.61	0.68	0.72	0.74	0.73	0.80	0.82	0.76
China_3	0.47	0.50	0.46	0.66	0.64	0.57	0.54	0.70	0.66	0.69	0.77	0.77	1.00	1.00	1.00	0.91	0.91	0.70	0.68	0.70	0.80	0.77	0.71	0.68	0.69	0.94	0.94	0.94	0.82	0.83	0.61	0.68	0.72	0.73	0.73	0.80	0.82	0.76
Pasteur_2	0.55	0.55	0.53	0.70	0.68	0.62	0.59	0.74	0.71	0.73	0.75	0.75	0.91	0.91	0.91	1.00	0.98	0.71	0.71	0.71	0.80	0.79	0.75	0.71	0.72	0.86	0.86	0.86	0.80	0.80	0.67	0.73	0.75	0.75	0.75	0.78	0.78	0.75
Pasteur_3	0.55	0.55	0.53	0.71	0.70	0.64	0.61	0.75	0.72	0.74	0.75	0.75	0.91	0.91	0.91	0.98	1.00	0.72	0.72	0.72	0.82	0.80	0.76	0.73	0.74	0.87	0.87	0.87	0.81	0.81	0.69	0.74	0.76	0.77	0.76	0.79	0.79	0.77
Tokyo_1	0.67	0.68	0.67	0.89	0.87	0.95	0.93	0.97	0.96	0.97	0.93	0.93	0.69	0.70	0.70	0.71	0.72	1.00	1.00	1.00	0.89	0.88	0.89	0.89	0.89	0.74	0.74	0.74	0.87	0.87	0.85	0.87	0.98	0.98	0.98	0.87	0.85	0.88
Tokyo_2	0.68	0.68	0.67	0.90	0.88	0.95	0.94	0.97	0.97	0.97	0.92	0.92	0.68	0.69	0.68	0.71	0.72	1.00	1.00	0.99	0.89	0.89	0.89	0.90	0.89	0.73	0.73	0.73	0.87	0.86	0.86	0.88	0.98	0.98	0.98	0.87	0.84	0.87
Tokyo_3	0.68	0.69	0.68	0.89	0.87	0.94	0.92	0.96	0.96	0.96	0.94	0.93	0.70	0.70	0.70	0.71	0.72	1.00	0.99	1.00	0.89	0.88	0.89	0.89	0.88	0.74	0.74	0.74	0.88	0.87	0.84	0.87	0.98	0.98	0.98	0.88	0.86	0.88
Tice_1	0.62	0.62	0.61	0.94	0.93	0.86	0.84	0.90	0.89	0.90	0.87	0.87	0.80	0.80	0.80	0.80	0.82	0.89	0.89	0.89	1.00	0.99	0.96	0.95	0.96	0.84	0.85	0.85	0.94	0.94	0.92	0.95	0.90	0.90	0.89	0.94	0.91	0.94
Tice_2	0.63	0.62	0.62	0.95	0.94	0.86	0.85	0.89	0.89	0.89	0.85	0.85	0.77	0.77	0.77	0.79	0.80	0.88	0.89	0.88	0.99	1.00	0.96	0.96	0.96	0.82	0.82	0.82	0.93	0.93	0.94	0.96	0.89	0.89	0.88	0.93	0.90	0.94
Copenhager	0.68	0.66	0.67	0.97	0.97	0.89	0.88	0.90	0.90	0.90	0.82	0.82	0.71	0.71	0.71	0.75	0.76	0.89	0.89	0.89	0.96	0.96	1.00	0.99	0.99	0.75	0.76	0.76	0.90	0.89	0.95	0.97	0.89	0.89	0.88	0.90	0.86	0.91
Copenhager	0.62	0.60	0.62	0.98	0.98	0.90	0.89	0.89	0.90	0.90	0.81	0.81	0.68	0.68	0.68	0.71	0.73	0.89	0.90	0.89	0.95	0.96	0.99	1.00	1.00	0.73	0.73	0.73	0.89	0.88	0.97	0.98	0.89	0.88	0.88	0.89	0.85	0.91
Copenhager	0.63	0.61	0.62	0.98	0.98	0.89	0.88	0.89	0.89	0.89	0.81	0.81	0.69	0.69	0.69	0.72	0.74	0.89	0.89	0.88	0.96	0.96	0.99	1.00	1.00	0.74	0.75	0.75	0.90	0.89	0.97	0.97	0.88	0.88	0.88	0.90	0.86	0.92
Phipps_1	0.49	0.52	0.47	0.71	0.69	0.63	0.60	0.74	0.71	0.73	0.80	0.80	0.94	0.94	0.94	0.86	0.87	0.74	0.73	0.74	0.84	0.82	0.75	0.73	0.74	1.00	1.00	1.00	0.86	0.86	0.67	0.73	0.76	0.77	0.77	0.83	0.85	0.80
Phipps_2	0.49	0.52	0.47	0.72	0.70	0.63	0.60	0.74	0.71	0.73	0.80	0.80	0.94	0.94	0.94	0.86	0.87	0.74	0.73	0.74	0.85	0.82	0.76	0.73	0.75	1.00	1.00	1.00	0.86	0.87	0.68	0.74	0.76	0.77	0.77	0.84	0.85	0.80
Phipps_3	0.49	0.52	0.47	0.72	0.70	0.63	0.60	0.74	0.71	0.73	0.80	0.80	0.94	0.94	0.94	0.86	0.87	0.74	0.73	0.74	0.85	0.82	0.76	0.73	0.75	1.00	1.00	1.00	0.86	0.87	0.68	0.74	0.76	0.77	0.77	0.83	0.85	0.80
Connaught_	0.59	0.62	0.59	0.89	0.87	0.79	0.77	0.86	0.84	0.85	0.88	0.88	0.82	0.82	0.82	0.80	0.81	0.87	0.87	0.88	0.94	0.93	0.90	0.89	0.90	0.86	0.86	0.86	1.00	1.00	0.85	0.88	0.88	0.88	0.89	0.98	0.97	0.97
Connaught_	0.58	0.62	0.58	0.88	0.86	0.78	0.75	0.85	0.83	0.84	0.88	0.88	0.83	0.83	0.83	0.80	0.81	0.87	0.86	0.87	0.94	0.93	0.89	0.88	0.89	0.86	0.87	0.87	1.00	1.00	0.83	0.87	0.87	0.87	0.88	0.97	0.97	0.96
Prague_1	0.58	0.54	0.58	0.97	0.98	0.90	0.90	0.86	0.88	0.87	0.75	0.75	0.61	0.61	0.61	0.67	0.69	0.85	0.86	0.84	0.92	0.94	0.95	0.97	0.97	0.67	0.68	0.68	0.85	0.83	1.00	0.99	0.85	0.84	0.84	0.85	0.79	0.88
Prague_3	0.61	0.58	0.60	0.97	0.97	0.89	0.89	0.89	0.90	0.90	0.79	0.79	0.68	0.68	0.68	0.73	0.74	0.87	0.88	0.87	0.95	0.96	0.97	0.98	0.97	0.73	0.74	0.74	0.88	0.87	0.99	1.00	0.88	0.88	0.87	0.88	0.82	0.89
Birkhaug_1	0.69	0.69	0.68	0.89	0.87	0.94	0.92	0.98	0.97	0.98	0.93	0.92	0.72	0.72	0.72	0.75	0.76	0.98	0.98	0.98	0.90	0.89	0.89	0.89	0.88	0.76	0.76	0.76	0.88	0.87	0.85	0.88	1.00	1.00	1.00	0.87	0.85	0.87
Birkhaug_2	0.68	0.69	0.68	0.89	0.87	0.93	0.92	0.98	0.97	0.98	0.93	0.93	0.73	0.74	0.73	0.75	0.77	0.98	0.98	0.98	0.90	0.89	0.89	0.88	0.88	0.77	0.77	0.77	0.88	0.87	0.84	0.88	1.00	1.00	1.00	0.87	0.85	0.87
Birkhaug_3	0.67	0.68	0.67	0.89	0.86	0.93	0.91	0.97	0.97	0.97	0.93	0.93	0.73	0.73	0.73	0.75	0.76	0.98	0.98	0.98	0.89	0.88	0.88	0.88	0.88	0.77	0.77	0.77	0.89	0.88	0.84	0.87	1.00	1.00	1.00	0.88	0.86	0.88
Frappier_1	0.60	0.62	0.60	0.90	0.88	0.81	0.78	0.85	0.84	0.85	0.88	0.87	0.80	0.80	0.80	0.78	0.79	0.87	0.87	0.88	0.94	0.93	0.90	0.89	0.90	0.83	0.84	0.83	0.98	0.97	0.85	0.88	0.87	0.87	0.88	1.00	0.99	0.99
Frappier_2	0.58	0.62	0.58	0.86	0.83	0.75	0.72	0.81	0.79	0.81	0.88	0.88	0.82	0.82	0.82	0.78	0.79	0.85	0.84	0.86	0.91	0.90	0.86	0.85	0.86	0.85	0.85	0.85	0.97	0.97	0.79	0.82	0.85	0.85	0.86	0.99	1.00	0.98
Frappier_3	0.61	0.63	0.62	0.92	0.91	0.83	0.81	0.85	0.84	0.85	0.86	0.86	0.76	0.76	0.76	0.75	0.77	0.88	0.87	0.88	0.94	0.94	0.91	0.91	0.92	0.80	0.80	0.80	0.97	0.96	0.88	0.89	0.87	0.87	0.88	0.99	0.98	1.00

Table S7: Proteomic of BCG Japan, Birkahug, Pasteur, Phipps and Danish compared to *M. bovis*. List of all identified proteins.

Mb Number	GenBank ID	Molecular Weig	*M.bovis*	Pasteur	Phipps	Danish	Tokyo	Birkhaug
Mb0448	31791618	57 kDa	1	1.7	1.9	1.6	2.3	2.5
Mb1423	31792582	21 kDa	0.9	0.8	1	0.6	0.8	0.6
Mb2268	31793424	13 kDa	1	0.5	0.6	0.5	0.5	0.6
Mb0485	31791655	22 kDa	1.1	0.9	1	0.7	1.3	1.2
Mb3452c	31794599	11 kDa	1	2.2	3.2	1.6	2.3	3.7
Mb0754	31791919	20 kDa	0.8	2.5	2.2	2	1.9	2.7
Mb0358	31791528	67 kDa	1	0.8	1.7	1.1	1.3	1.9
Mb0359	31791529	25 kDa	1	1.1	2.1	1.3	1.7	2.3
Mb2169c	31793325	28 kDa	0.9	0.6	0.8	0.9	1.1	0.6
Mb2489c	31793643	51 kDa	1.1	1.2	1.6	1.2	1.3	1.6
Mb1243	31792404	8 kDa	1.1	0.5	0.6	0.4	1.5	1.3
Mb3945	31795087	13 kDa	1	0.4	0.4	0.4	0.5	0.7
Mb3672c	31794818	7 kDa	1	2	0.8	0.6	1.1	1.7
Mb2965c	31794117	224 kDa	1	2.2	1.3	1.1	1.6	1.9
Mb0671	31791836	13 kDa	1	1.2	1.1	0.9	0.9	1.6
Mb0471	31791641	49 kDa	1	1.8	1.6	1.5	1.6	1.7
Mb3010c	31794162	22 kDa	1	0.7	1.5	0.6	1.6	1.7
Mb1858	31793017	17 kDa	1.2	1.9	0.8	0.9	1.7	1.6
Mb3743c	31794888	13 kDa	1	0.8	1.1	0.7	1	0.7
Mb2765c	31793918	29 kDa	1	0.9	1	0.9	0.9	0.9
Mb0837c	31792002	10 kDa	1	0.5	0.6	0.3	0.4	0.2
Mb3628c	31794774	12 kDa	0.9	0.3	1	0.7	0.6	0.7
Mb1109c	31792271	18 kDa	1.1	1.3	1.5	2.2	1.1	2.5
Mb3485c	31794632	19 kDa	1	2.7	1.8	1.5	2	2.9
Mb2227c	31793383	13 kDa	1	1	1.3	0.7	1.5	1.2
Mb3910c	31795054	12 kDa	1	2.5	2	2.1	2.7	2.3
Mb2244	31793400	54 kDa	1	0.7	0.9	0.7	0.7	0.8
Mb2135c	31793291	7 kDa	1.2	1	1.6	0.7	1.7	1.2
Mb3451c	31794598	56 kDa	1	1.8	1.9	1.2	2.3	2.9
Mb3817	31794961	17 kDa	1	0.6	0.8	0.8	1.1	1
Mb0007	31791184	31 kDa	1	1.7	1.9	1.8	1.6	2
Mb1272	31792433	34 kDa	1	2.2	1.2	1.1	2.5	1.9
Mb2960	31794112	159 kDa	1	1.1	1	1.1	1	1.7
Mb0704	31791869	44 kDa	0.9	2.1	2.2	1.9	2	2.6
Mb0130	31791304	35 kDa	1	1.3	2	1.1	1	1.2
Mb0736	31791901	11 kDa	1	0.7	1.3	0.8	1.3	1.4
Mb0737	31791902	21 kDa	1	2	1.5	1.7	1.2	1.5
Mb3897	31795041	20 kDa	0.9	0.9	0.9	0.8	0.7	0.6
Mb1329	31792490	65 kDa	1	1	0.8	0.7	0.9	1.1

Mb1656	31792816	53 kDa	1	1.7	1.6	2.5	1.9	2.1
Mb1868c	31793027	80 kDa	1	1.7	1.8	1.1	1.2	1.6
Mb2750	31793903	50 kDa	1	0.6	0.9	0.9	1.1	1
Mb0744	31791909	16 kDa	1	3.1	1.6	0.9	2.5	2.9
Mb2718c	31793871	11 kDa	1.1	1	0.9	0.6	1.9	1.5
Mb0020c	31791197	56 kDa	1	0.6	1.1	1.5	0.8	1.1
Mb0386	31791556	8 kDa	0.9	0.2	0.4	0.3	0.3	0.4
Mb3486c	31794633	38 kDa	1	1.4	2	1.9	1.6	2.3
Mb1950	31793108	85 kDa	1	0.9	0.9	1	1.2	1
Mb3652	31794798	18 kDa	1	2.1	2.1	2.7	4.7	2.2
Mb0477	31791647	31 kDa	1	1.1	1.3	1.9	1.7	1.8
Mb2503c	31793657	177 kDa	1	0.9	0.9	0.8	0.9	0.9
Mb0009	31791186	19 kDa	1.1	0.6	1	0.8	1.3	1.4
Mb1662	31792822	15 kDa	1.2	0.6	0.6	0.6	1.5	1.8
Mb2898	31794050	22 kDa	1	0.2	0.3	0.3	0.3	0.4
Mb1255	31792416	54 kDa	1	1.5	0.8	0.7	1	1.3
Mb3055c	31794207	28 kDa	1	1.1	1.1	1.7	0.7	1.1
Mb1665c	31792825	10 kDa	0.9	0.3	0.7	0.4	0.5	0.4
Mb2933c	31794085	17 kDa	1	1.6	1.9	1.8	1.6	1.8
Mb0368	31791538	30 kDa	1.1	0.3	0.4	0.5	0.5	0.7
Mb3831c	31794975	69 kDa	1	0.7	0.8	0.9	0.8	0.9
Mb2324	31793480	9 kDa	1	0.2	0.3	0.2	0.3	0.3
Mb0254c	31791426	71 kDa	1	1.7	1.3	0.8	1	1.1
Mb2906c	31794058	21 kDa	1.1	0.3	0.3	0.3	0.3	0.3
Mb3809	31794953	20 kDa	1	1.1	1.3	1.1	0.9	1.1
Mb2238	31793394	57 kDa	1	1.3	1.6	1.1	1.8	1.7
Mb2808c	31793961	10 kDa	1	2.1	1.6	1.6	1.5	1.7
Mb3627c	31794773	94 kDa	0.9	1.2	1.2	1.2	0.7	0.7
Mb0360	31791530	41 kDa	1	2.1	1.8	1.2	1.9	2.3
Mb3054c	31794206	32 kDa	1.1	1.3	1.1	1.3	1.7	1.4
Mb1427	31792586	43 kDa	1	0.8	0.6	0.7	0.8	0.8
Mb1907	31793066	18 kDa	1	0.5	0.4	0.5	0.5	0.4
Mb0729	31791894	9 kDa	1	2.1	1.6	1.5	1.7	2
Mb0321	31791492	14 kDa	0.9	0.7	0.9	1	0.6	0.6
Mb0581c	31791748	18 kDa	1	0.9	1.1	0.8	1.2	0.9
Mb3133	31794285	49 kDa	1	0.8	0.8	0.8	0.9	1
Mb3951c	31795093	21 kDa	1	1.7	1.6	1.1	1.1	1.2
Mb2952c	31794104	27 kDa	1	1	1.2	1.5	1.5	1.2
Mb2494	31793648	94 kDa	1	0.9	1.2	1.1	1.1	0.9
Mb1067c	31792230 (+2)	11 kDa	0.9	0.9	0.9	0.9	0.7	0.8
Mb0391c	31791561	93 kDa	1	1	1.1	1.6	1	1
Mb1280c	161511534	136 kDa	1	0.9	0.7	0.8	1	0.9

Mb1926	31793085	7 kDa	1	0.4	0.5	0.4	0.6	0.8
Mb0811	31791976	9 kDa	1	0.8	1.1	0.8	0.8	0.9
Mb2971c	31794123	218 kDa	1	1.2	1	1.2	1.4	1
Mb1051	31792214	45 kDa	1	2	1.5	1.6	2.1	1.9
Mb3250c	31794402	24 kDa	1	2.6	2.3	1.6	1.7	1.9
Mb1584	31792743	16 kDa	1	1.1	0.9	0.7	1	0.8
Mb1484c	31792643	76 kDa	1	0.8	0.8	0.9	0.9	0.9
Mb1857	31793016	14 kDa	1	0.5	0.6	0.6	0.5	0.5
Mb1342	31792503	53 kDa	1	0.9	0.7	1.1	0.7	0.7
Mb0670	31791835	18 kDa	1	1.4	1.1	1.4	1.2	1.8
Mb2254c	31793410	27 kDa	1	1.3	1.8	0.8	1.2	1.2
Mb1670	31792830	15 kDa	1	1.2	1.1	1.3	1.1	1.2
Mb1515	31792674	41 kDa	1.1	1.4	1.2	1.2	1.8	1.4
Mb2914c	31794066	31 kDa	1	1.5	1.7	1.6	1.8	1.7
Mb3882	31795026	14 kDa	1	1.1	1.4	1.3	1.1	1
Mb2121c	31793277	9 kDa	0.9	1	1.1	1	0.7	0.7
Mb2057c	31793214	16 kDa	1	0.5	15.7	0.9	2.2	1.7
Mb3492	31794639	31 kDa	1	0.8	0.5	0.6	0.8	1.6
Mb2435	31793591	9 kDa	1	0.8	0.6	0.6	1.5	1.1
Mb3702c	31794848	15 kDa	0.9	0.9	0.9	0.5	0.3	0.6
Mb0730	31791895	15 kDa	1	3.4	2.4	2.3	2.8	3.2
Mb1338	31792499	18 kDa	1	2.1	1.7	1.6	2.5	2.3
Mb1123	31792286	45 kDa	1	1.4	1.3	1.8	1.2	1.2
Mb1363	31792524	96 kDa	1	0.9	0.9	0.8	0.9	0.8
Mb3871	31795015	20 kDa	1	1.1	0.5	0.5	0.4	0.4
Mb0895	31792059	15 kDa	1	0.7	0.9	0.7	0.6	0.7
Mb1652	31792812	23 kDa	1	1	1	1.1	1.2	1
Mb1891	31793050	33 kDa	1	0.7	0.7	0.6	1.1	0.7
Mb3889c	31795033	166 kDa	1	1.9	1.6	1.3	1.9	1.8
Mb2521	31793673	8 kDa	1	0.7	1.3	0.8	1.1	1.2
Mb2913c	31794065	29 kDa	0.9	2.1	1.1	0.8	1.8	2.2
Mb3614c	31794760	18 kDa	1	1.3	1.6	1.3	1.1	1.1
Mb1903c	31793062	45 kDa	1	0.6	0.3	0.4	0.5	0.4
Mb0742	31791907	23 kDa	1	3.2	1.6	1.8	2.5	2.9
Mb3879	31795023	15 kDa	1	1	1	1.2	1.3	1
Mb1471	31792630	36 kDa	1	1.7	1.3	1	0.6	0.7
Mb2454	31793608	22 kDa	1	3.4	4.3	1.9	1.8	2.9
Mb3487c	31794634	23 kDa	1	2.6	2.2	1.9	2.1	2.9
Mb0152	31791325	55 kDa	1	0.6	0.7	0.6	0.6	0.7
Mb0256c	31791428	11 kDa	1.2	0.4	0.9	0.6	1.7	2
Mb0722	31791887	24 kDa	1	2.3	1.7	1.5	1.4	2.5
Mb2320	31793476	35 kDa	1	1.1	0.8	1.4	0.9	0.9

Mb2468c	31793622	9 kDa	1	1.2	1.3	0.6	0.5	0.6
Mb2270	31793426	46 kDa	1	0.7	0.9	1.1	0.9	1.6
Mb2618c	31793771	60 kDa	1	1.1	0.9	1.1	0.9	0.9
Mb2928c	31794080	13 kDa	1	2.3	1.8	1.6	1.5	1.9
Mb2504c	31793658	62 kDa	1	1.2	1.1	1.4	1.1	1.2
Mb0686	31791851	129 kDa	1	1.2	1.3	1.7	2	2.5
Mb2207c	31793363	16 kDa	1	0.7	1	1.4	1.2	0.9
Mb2471c	31793625	103 kDa	1	0.6	0.8	0.9	1.1	1.3
Mb3276c	31794428	54 kDa	1	2.3	2.1	2.2	2.5	2.7
Mb3880	31795024	24 kDa	1	0.6	0.9	0.9	1	0.8
Mb1210	31792371	12 kDa	1	1.1	2	0.6	0.9	1.2
Mb0306	31791477	8 kDa	1	1	1.1	0.7	1.2	1
Mb0055	31791231	17 kDa	1	1.8	1.6	1.6	1.5	1.9
Mb2242	31793398	27 kDa	0.9	1.2	0.6	1.3	0.8	0.7
Mb3303c	31794455	18 kDa	1.1	1.2	0.8	1.1	1.4	1.3
Mb0951	31792115	38 kDa	1	3.4	2.5	3	1.7	0.8
Mb2171c	31793327	28 kDa	0.9	2	1.1	1.9	0.7	0.7
Mb3071	31794223	37 kDa	1	0.7	0.5	0.6	0.5	0.6
Mb2563c	31793716	20 kDa	1	0.5	1.2	1	1.5	1
Mb2025	31793182	27 kDa	1	0.7	0.6	0.9	1.1	6.3
Mb3159	31794311	38 kDa	0.9	2.6	2.1	1.6	0.6	0.7
Mb0189	31791361	30 kDa	1	0.9	0.9	1	0.8	0.9
Mb3440	31794587	33 kDa	1	0.4	0.9	0.9	12	0.5
Mb0057	31791233	16 kDa	1	2.2	2.9	2.3	2.5	3.1
Mb2864c	31794016	94 kDa	1	1.6	1.3	1.5	1.2	1.8
Mb2886c	31794038	31 kDa	1	0.8	0.5	1.1	0.6	0.6
Mb3392	31794540	10 kDa	1	0.9	1.1	0.6	1.1	1
Mb1900c	31793059	44 kDa	1	0.8	0.9	0.8	1	1
Mb3700	31794846	25 kDa	1	0.5	0.5	0.5	0.5	0.7
Mb2074c	31793231	431 kDa	1	1.6	1.3	1.6	1.6	2
Mb0823	31791988	46 kDa	1.1	0.5	1.2	0.8	0.9	1.5
Mb3577	31794723	17 kDa	1	1.1	0.8	0.9	1.1	0.8
Mb2265	31793421	100 kDa	1	0.8	0.8	0.9	0.8	0.6
Mb3895	31795039	11 kDa	1.1	1	0.8	0.9	1.4	1.3
Mb3330c	31794482	63 kDa	1	2.1	1.4	1	1.6	1.8
Mb0922c	31792086	10 kDa	1	0.7	1.3	1	0.9	1.2
Mb0658	31791823	25 kDa	1	1	1.1	1.1	1.3	1
Mb1669	31792829	7 kDa	1	0.5	1.1	0.4	1.3	1.4
Mb3219c	31794371	7 kDa	0.9	0.5	0.6	0.5	0.3	0.4
Mb3297	31794449	10 kDa	0.9	0.7	1.3	1.7	0.9	0.8
Mb3331c	31794483	49 kDa	1.1	3.5	2.6	1	3.3	3.7
Mb1018c	31792182	11 kDa	0.9	1.6	2.4	1.3	0.8	0.7

Mb3069c	31794221	64 kDa	1	1.1	1	1.2	0.9	0.9
Mb2722	31793875	58 kDa	1	1.4	1.3	1.6	1.3	2.1
Mb2774c	31793927	31 kDa	1	1.8	1.8	1.5	0.7	1.3
Mb3737	161511524	71 kDa	1	0.9	1.1	1.2	1	1.1
Mb3693	31794839	19 kDa	1.1	2	1.1	1	1.8	1.5
Mb0653	31791818	6 kDa	1	2.9	3.1	2.7	2.1	3.3
Mb3251	31794403	30 kDa	1	1.9	0.9	0.9	0.8	0.8
Mb0584	31791751	10 kDa	1.2	0.6	3.6	0.7	1.2	2.3
Mb3670c	31794816	102 kDa	1	1.4	1.3	1.2	1.6	1.2
Mb0659	31791824	15 kDa	1	1.4	2.5	1.7	1.5	2.1
Mb0976	31792140	41 kDa	1	2.2	1.8	1.6	2.1	2.3
Mb3308	31794460	59 kDa	1	0.9	0.9	1.1	0.9	0.7
Mb1340	31792501	59 kDa	1	0.7	0.9	1.1	1.5	1.5
Mb3177	31794329	23 kDa	1	1.7	1.7	1.9	1.6	1.8
Mb3644c	31794790	20 kDa	1	0.6	1.1	1.2	0.6	0.9
Mb0794	31791959	16 kDa	1	2.2	1.9	1.6	0.6	1.3
Mb0508	31791678	33 kDa	1	0.6	1.1	1.1	1.1	1
Mb0720	31791885	11 kDa	1	3.2	1.7	2.4	2.9	3.1
Mb1359	31792520	32 kDa	1.2	1.6	1.5	1.5	1.7	1.9
Mb3539c	31794685	52 kDa	1	0.5	0.5	0.9	0.6	0.7
Mb2620	31793773	47 kDa	1	0.8	0.9	0.6	1.4	1.1
Mb3472c	31794619	16 kDa	1	2.3	2.1	2	2.5	3.1
Mb3894	31795038	44 kDa	1.1	0.6	1	0.5	1.5	1.3
Mb0067c	31791243	83 kDa	1	1.5	1.1	1.3	0.8	0.9
Mb0238	31791410	36 kDa	1	0.3	0.4	0.3	1.6	1.7
Mb0249	31791421	46 kDa	1	1.3	0.8	1.3	0.6	0.9
Mb1524	31792683	41 kDa	1	0.8	0.7	1.1	1.3	0.7
Mb0292	31791463	145 kDa	1	0.9	1	1.3	1.2	1.1
Mb2191c	31793347	15 kDa	1.2	0.9	0.8	1.2	2	1.8
Mb2218	31793374	47 kDa	1	0.8	0.7	0.9	1.4	1.3
Mb2610	31793763	34 kDa	0.9	1.1	0.7	1.2	0.4	0.5
Mb2635c	31793788	27 kDa	1	1.7	1.4	1.4	1	1.1
Mb0908c	31792072	40 kDa	1	1.1	1	1.1	1	0.8
Mb2977	31794129	45 kDa	1	1	0.9	1.2	1	1
Mb3102c	31794254	33 kDa	1	0.9	1	1.2	1	1
Mb3175	31794327	85 kDa	1	1.8	1.9	1.1	1.6	1.7
Mb3893	31795037	41 kDa	1	1.7	1.2	1.5	0.7	1.1
Mb0047c	31791223	40 kDa	1	3.3	3.2	2.5	4.3	4.2
Mb1554c	31792713	224 kDa	1	0.7	0.7	1.3	0.8	1.1
Mb0735	31791900	13 kDa	1	2.5	1.9	2.2	2	2.4
Mb3761c	31794906	49 kDa	1	0.3	0.3	0.5	0.4	0.4
Mb0779c	31791944	25 kDa	1	0.9	0.9	0.6	0.6	1

Mb0854c	31792019	30 kDa	1	1.2	1.2	1.2	0.9	1.1
Mb1501	31792660	12 kDa	0.9	1.3	1.8	1.3	0.7	0.7
Mb2321c	31793477	73 kDa	1	0.9	1	1.6	1.7	1.5
Mb2397c	31793553	17 kDa	0.9	1.3	1.9	0.8	0.5	0.6
Mb2002c	31793159	25 kDa	1.1	0	0	0	0.5	0.4
Mb0630c	31791796	93 kDa	1	1	0.9	0.9	1	0.8
Mb2638c	31793791	31 kDa	1	1.5	1.1	1.4	1.2	0.9
Mb2993c	31794145	27 kDa	1	1.8	1.3	1.8	0.8	1.1
Mb1967	31793124	17 kDa	1	2.7	2.5	2.3	2.7	2.2
Mb3072c	31794224	13 kDa	1.1	1	1.2	0.9	1	1.3
Mb3268c	31794420	106 kDa	1	0.9	1	1	1	1
Mb0615c	31791781	8 kDa	0.9	0.8	1.5	0.7	0.7	0.7
Mb1139c	31792302	23 kDa	1.1	1	1	1.3	1.2	1.5
Mb0745	31791910	66 kDa	1	1.2	0.7	0.8	1.4	1
Mb2592	31793745	36 kDa	1	1	0.9	3	0.8	1
Mb3489c	31794636	14 kDa	1	2	3.2	2.6	2.1	2.9
Mb0056	31791232	10 kDa	1	2.3	2	1.8	2.2	2.1
Mb2966	31794118	63 kDa	1	1	1.2	1.1	1	1
Mb3713c	31794859	17 kDa	1	1.1	1	0.9	0.7	0.9
Mb3157c	31794309	23 kDa	1	1	1.1	1	2	1.4
Mb0162	31791335	49 kDa	1	1.5	1.3	1.3	1.3	1.4
Mb3421c	31794569	30 kDa	1.1	1.3	1.2	1.7	1.4	1.4
Mb0091	31791266	25 kDa	1	1.4	1.1	1.6	1.3	0.9
Mb0657	31791822	17 kDa	1	0.7	1	0.6	1	1
Mb2655	31793808	30 kDa	1	0.6	0.6	0.6	0.4	0.7
Mb2174c	31793330	39 kDa	1	2	1.2	3.8	2	2
Mb0727	31791892	30 kDa	1	2.1	1.6	1.4	1.8	1.9
Mb3911c	31795055	48 kDa	1	1.1	1	1.1	1.2	0.9
Mb3876	31795020	23 kDa	1.1	1.4	1.4	1	1.1	1.6
Mb0212c	31791384	101 kDa	1.1	0.7	1	0.7	1.2	1.4
Mb0543	31791712	43 kDa	1	0.9	0.7	0.9	0.8	0.8
Mb1277c	31792438	29 kDa	1.1	1.5	1.2	1.2	1.4	1.4
Mb0008c	31791185	16 kDa	1	0.6	0.7	0.8	0.6	0.6
Mb1960	31793117	66 kDa	1	1	1	1.1	1	1
Mb1398c	31792559	28 kDa	0.9	1.9	2.3	3.6	0.7	0.7
Mb1483c	31792642	41 kDa	1	1.2	1	1.5	1.1	0.9
Mb2472c	31793626	15 kDa	1	0.7	0.5	0.7	0.5	0.8
Mb2730	31793883	25 kDa	1	1.4	1.2	1.8	0.8	1.2
Mb2806c	31793959	80 kDa	1	2	0.9	1.1	1.3	2.3
Mb2940c	31794092	55 kDa	1	1.5	1.3	1.7	1.1	1.1
Mb1511c	31792670	102 kDa	1	0.6	0.9	0.8	0.9	0.9
Mb3703c	31794849	6 kDa	1.2	0.6	0.6	0.4	1.5	1.8

Mb3725	31794870	25 kDa	1	1.1	0.7	1.2	0.9	1
Mb0160	31791333	38 kDa	1	1.5	1.3	1.8	1.4	1.4
Mb1519	31792678	26 kDa	1	1.2	1	1.3	1	0.9
Mb0178	31791350	57 kDa	1.1	0.6	0.4	1	0.9	1.3
Mb2481c	31793635	40 kDa	1	1.1	1	0.9	0.9	1.8
Mb1183	31792345	13 kDa	0.9	1.1	0.8	1.3	0.3	0.5
Mb1370	31792531	10 kDa	1.1	1	1.1	0.7	5.9	1.6
Mb3163	31794315	50 kDa	1	2.7	2	2.4	1.5	1.4
Mb1439	31792598	18 kDa	0.9	0.9	0.8	1	0.4	0.4
Mb1498	31792657	29 kDa	1	1.5	1.8	1.9	1.4	1.2
Mb1917c	31793076	22 kDa	1	1.9	1.9	1.5	1.6	1.2
Mb2076	31793233	13 kDa	1	1.2	1.4	1.2	1	1.2
Mb2740c	31793893	72 kDa	1.1	0.6	0.7	0.8	1.3	1.3
Mb1812	31792972	153 kDa	1	0.7	0.5	0.6	0.7	0.6
Mb0049c	31791225	31 kDa	1	1.2	1.1	1.4	0.9	1.1
Mb0475	31791645	30 kDa	1	0.8	0.9	1	1.1	1.1
Mb0562c	31791730	35 kDa	1	2.1	1.6	1.4	0.8	1.3
Mb0655	31791820	15 kDa	1	1.4	1.2	1.7	0.7	1.2
Mb0838c	31792003 (+1)	31 kDa	1	1	0.8	1.5	1	0.8
Mb1760	31792919	55 kDa	1	0.7	0.8	1.3	1.3	1.6
Mb0920	31792084	48 kDa	1	0.6	0.5	1.1	1	0.9
Mb2223c	31793379	40 kDa	1	1.8	1.6	1.5	1.7	1.7
Mb3350c	31794502	9 kDa	1.1	1.3	1.6	0.9	1.3	1.7
Mb3750	31794895	27 kDa	1	1	1.1	0.8	1	0.7
Mb1627	31792787	23 kDa	1.1	0.7	0.6	0.7	1	1.3
Mb0743	31791908	7 kDa	1	3.1	2	2.1	2.3	2.7
Mb0660	31791825	25 kDa	1	2.8	2.1	2	2.2	2.6
Mb1099c	31792261	27 kDa	1	1.1	0.8	1.3	1.1	0.8
Mb3008	31794160	83 kDa	1	1	1.2	1.4	0.8	1
Mb2565	31793718	25 kDa	1	0.6	0.6	0.7	1	0.8
Mb2615c	31793768	23 kDa	1	1	1	1.1	1.1	1.1
Mb0169	31791342	18 kDa	1	0.6	1.6	1.9	1.1	1.1
Mb3898	31795042	62 kDa	1	0.7	0.8	0.6	0.8	0.8
Mb2455	31793609	19 kDa	0.9	2.9	2	3.3	2.5	2
Mb2032	31793189	9 kDa	1	0.4	1.1	0.5	1.3	0.9
Mb0002	31791179	42 kDa	1	0.8	1	1.7	1	0.8
Mb2893c	31794045	40 kDa	1	1.3	1.3	1.2	1.5	1.3
Mb0248c	31791420	47 kDa	1	1.4	1.1	1.8	0.8	0.9
Mb0897	31792061	71 kDa	1.1	0.8	0.7	1.2	1.2	1.3
Mb2957	31794109	162 kDa	1	1.8	2.1	1.2	0.9	2.4
Mb1128c	31792291	50 kDa	1	1.1	1	0.6	1	1
Mb0253c	31791425	29 kDa	1	1.2	0.9	1.2	1	1.1

Mb3168c	31794320	39 kDa	1	0.4	0.6	0.5	0.7	0.8
Mb2374	31793530	70 kDa	1	0.8	0.7	1.3	0.8	0.8
Mb2900	31794052	19 kDa	1	0.2	0.2	0.2	1	0.7
Mb0370c	31791540	37 kDa	1	1.3	1.6	1.2	1.1	1.1
Mb3745c	31794890	16 kDa	1	1.4	0.9	1.3	1	1.1
Mb0159c	31791332	44 kDa	1	0.6	0.6	0.7	0.7	0.6
Mb0476	31791646	47 kDa	1	2.1	1.9	1.7	1.2	2.5
Mb3234c	31794386	9 kDa	1	1	1.1	1	1.2	0.9
Mb0784c	31791949	40 kDa	1	0.7	0.8	1.1	0.7	0.7
Mb0649c	31791814	24 kDa	1	0.4	0.7	1.5	0.4	1.3
Mb0006	31791183	92 kDa	1	1.6	1.5	1.1	1.1	1.3
Mb0687	31791852	147 kDa	1	1.8	1.6	1.6	1.5	2
Mb1103c	31792265	43 kDa	1	0.7	0.8	0.8	0.7	0.8
Mb0721	31791886	23 kDa	1	1.4	1.1	1.3	1.7	1.6
Mb1010	31792174	18 kDa	1.1	1.6	1.8	1.4	1.7	1.6
Mb1273	31792434	10 kDa	1	0.7	1.2	0.8	1.6	1.1
Mb1777	31792936	27 kDa	1.1	0.9	0.7	1	1.6	1.3
Mb3036c	31794188	51 kDa	1	1	1	0.9	1.2	1.1
Mb2012c	31793169	12 kDa	1	0.8	1	0.7	0.8	1.1
Mb3016c	31794168	54 kDa	1	1	0.9	1	0.9	0.9
Mb2727c	31793880	9 kDa	1	1.8	1.9	0.9	0.9	1.3
Mb0595c	31791762	18 kDa	1	0.8	0.6	1.1	0.6	0.6
Mb2482c	31793636	69 kDa	1	0.9	0.9	1.1	1	1.3
Mb3027c	31794179	18 kDa	1	1.3	1.2	1.6	1.1	1.2
Mb2487c	31793641	24 kDa	1	0.9	0.7	1.3	0.8	0.6
Mb3313	31794465	64 kDa	0.8	0.7	1.8	1	0.7	0.8
Mb2077c	31793234	94 kDa	1	1	1.1	0.3	1.4	1.5
Mb3646c	31794792	40 kDa	1	0.2	0.4	0.3	0.4	0.4
Mb3927c	31795071	32 kDa	1	0.9	0.8	0.9	0.5	1
Mb3339	31794491	46 kDa	1	1.4	1.3	1.4	3.7	3.9
Mb3298	31794450	76 kDa	1	0.8	1	0.5	0.8	0.8
Mb0833c	31791998	7 kDa	1.2	0.7	1.2	0.4	1.7	1.8
Mb0250c	31791422	66 kDa	1	1.7	1.6	1.7	1.7	3.9
Mb1729c	31792889	22 kDa	1	1	0.9	0.8	1.2	1.1
Mb1164c	31792327	82 kDa	1	0.9	0.7	0.9	0.7	0.8
Mb1668	31792828	22 kDa	1	0.9	1	1.2	0.8	1
Mb0674	31791839	39 kDa	1	0.4	0.5	0.5	0.3	0.8
Mb3834c	31794978	36 kDa	1	2.3	1.9	1.6	1.7	2.1
Mb3952c	31795094	41 kDa	1	1.3	1.1	2.1	1.1	1.3
Mb1961c	31793118	17 kDa	1	0.4	0.5	0.4	0.9	0.7
Mb0447c	31791617	34 kDa	1	1.2	1.3	0.9	1.2	1
Mb1947c	31793105	36 kDa	1	0.8	1.1	1.2	0.7	1

Mb2526c	31793678	29 kDa	1	2.3	2.1	1.3	1.7	1.8
Mb2469c	31793623	11 kDa	1	1.2	1.4	1.3	17	1.6
Mb0725	31791890	11 kDa	1	2.4	2.1	2	1.9	2.5
Mb0043c	31791219	22 kDa	1	0.4	0.5	0.4	1	0.7
Mb2959	31794111	193 kDa	1	1.9	1.6	1.3	1.2	2.2
Mb0184	31791356	26 kDa	1	0.6	0.9	0.6	1.4	1.1
Mb0824	31791989	13 kDa	1	0.8	0.9	0.8	0.9	0.9
Mb0751	31791916	26 kDa	1	0.7	0.5	1	0.7	0.7
Mb3735c	31794880	36 kDa	1	1.4	1.1	1.3	1.1	1.2
Mb1153	31792316	36 kDa	1	1.3	1.2	1	0.8	1
Mb2394c	31793550	40 kDa	1	1.1	1.1	1.3	1.1	1.5
Mb3347	31794499	65 kDa	1	0.9	1.3	1.5	0.9	3.7
Mb2205c	31793361	13 kDa	1	1.3	1.4	1	1.4	1.1
Mb0702	31791867	18 kDa	1	1.7	1.6	1.5	1.8	2
Mb1856	31793015	31 kDa	1	0.8	1.5	1	1.3	1.2
Mb3948c	31795090	37 kDa	0.9	0.7	1.1	1.3	0.8	0.7
Mb3807c	31794951	42 kDa	1	0.7	0.8	0.8	0.8	1.1
Mb0153	31791326	30 kDa	1	0.9	1	1.4	1	0.8
Mb0054	31791230	11 kDa	1	0.9	1.1	1.1	0.9	0.9
Mb0158c	31791331	30 kDa	1	1.1	1.4	1.4	1.5	1
Mb3429c	31794577	56 kDa	1	1.2	1.3	1.1	1.1	1
Mb0177	31791349	54 kDa	1	0.6	0.6	0.8	0.5	0.8
Mb3026c	31794178	36 kDa	1	0.7	1.3	1.9	1.4	1.6
Mb0741	31791906	13 kDa	1	1	1.1	1.1	0.7	1
Mb1129c	31792292	35 kDa	1.2	1.4	1.1	1	1.9	1.9
Mb0014c	31791191	67 kDa	1	0.8	0.6	1.1	0.9	0.8
Mb1143	31792306	7 kDa	1	0.9	1.4	0.7	0.7	1
Mb0195c	31791367	59 kDa	1	0.8	1	0.9	0.9	0.8
Mb2652c	31793805	12 kDa	1	0.5	1	0.8	0.9	0.9
Mb2593	31793746	35 kDa	1	0.9	0.9	1.2	1	1
Mb3433	31794581	28 kDa	1	1	1.3	1.2	0.9	1
Mb0780	31791945	28 kDa	1	0.7	0.9	0.8	0.7	1
Mb1414	31792573	21 kDa	1	0.8	0.5	0.9	1.1	0.7
Mb2080	31793237	25 kDa	1	1	0.8	0.9	1	0.7
Mb0290	31791461	68 kDa	1	0.8	0.9	1.2	1.1	1.2
Mb0726	31791891	20 kDa	1	1.7	1.5	1.4	1.2	1.9
Mb3012c	31794164	50 kDa	1	0.6	0.7	0.9	0.6	0.7
Mb0977	31792141	31 kDa	1	1.1	1.2	1.3	1.6	1.7
Mb2976	31794128	31 kDa	1	2.2	2	2.1	1.9	1.6
Mb1637	31792797	28 kDa	0.9	1.5	0.8	1.4	0.7	0.6
Mb1639	31792799	28 kDa	1	1.3	1	1.4	0.9	1
Mb2549c	31793701	8 kDa	1	1.4	1	1.6	1	1

Mb2318	31793474	33 kDa	1	0.6	0.8	1.4	0.8	0.9
Mb2133c	31793289	27 kDa	1	1.4	0.7	1.7	0.9	1
Mb3601c	31794747	43 kDa	1	0.9	1.1	1	1	0.9
Mb3717	31794863	38 kDa	1.1	1.5	1.1	1.9	1.2	1.4
Mb2974c	161511529	64 kDa	1	1.6	1.3	1.4	1.6	1.4
Mb1343	31792504	13 kDa	1	0.7	0.6	0.6	1.6	1.5
Mb0236	31791408	63 kDa	1	0.3	0.4	0.4	0.5	0.4
Mb1339	31792500	49 kDa	1	1	0.8	1.2	0.9	0.9
Mb3605	31794751	22 kDa	1	1.6	1	1.9	1.3	0.9
Mb0478	31791648	33 kDa	1	1	0.9	1	1	1.1
Mb3278c	31794430	7 kDa	1	0.9	1.4	0.6	0.8	1
Mb1516	31792675	34 kDa	1	0.8	1	0.6	0.9	1.7
Mb0198	31791370	37 kDa	1	1.5	1.7	1.2	1.8	1.2
Mb1686	31792845	44 kDa	1	0.8	0.9	0.9	0.9	0.9
Mb0324	31791495	22 kDa	1	0.9	1.1	1.4	1	0.9
Mb0923	31792087	34 kDa	1	0.9	1.1	0.9	0.9	1.1
Mb1327	31792488	37 kDa	1	0.6	0.8	0.6	1	0.8
Mb1446c	31792605	25 kDa	1.1	1.2	1	1.2	1.3	1.3
Mb2016c	31793173	9 kDa	1	0.5	1.5	1.2	1.7	1.1
Mb0063	31791239	40 kDa	1	2	1.8	1.6	1.8	1.6
Mb1292c	31792453	17 kDa	1	0.6	0.4	0.6	0.7	0.6
Mb2134c	31793290	30 kDa	1	2.2	1.1	3.2	1.3	0.9
Mb2165c	31793321	48 kDa	1	1	0.9	0.8	0.6	1
Mb3491c	31794638	8 kDa	1	2.1	2.6	2.5	2.3	2.7
Mb0598c	31791765	24 kDa	1.1	1	0.7	1.2	0.8	1.4
Mb2955	31794107	63 kDa	1	3.5	1.9	1.7	2.1	4.3
Mb2197c	31793353	16 kDa	1	0.4	0.8	0.6	0.7	0.8
Mb0132	31791306	50 kDa	1	0.9	1	0.8	0.9	0.9
Mb0196	31791368	10 kDa	0.9	2.3	1.3	2	0.5	0.7
Mb3634c	31794780	43 kDa	1.1	0.7	1.1	1.5	1.2	1.4
Mb3215c	31794367	107 kDa	1	0.9	1	1	0.8	0.9
Mb3302c	31794454	42 kDa	1	0.9	0.9	0.9	0.7	0.9
Mb2508c	31793662	62 kDa	1	0.5	0.4	0.4	0.5	0.4
Mb0552	31791720	55 kDa	0.9	1	1.3	1.3	0.6	0.7
Mb0904	31792068	16 kDa	1	0.9	0.7	1.2	0.8	0.7
Mb0929	31792093	26 kDa	1	1	1.2	1.4	1	0.9
Mb2523c	31793675	41 kDa	1	2.3	1.6	2.5	1.5	1.1
Mb2250	31793406	56 kDa	1	1.6	1.2	1.6	1.1	1.1
Mb2699	31793852	23 kDa	1	0.7	1.2	1	1.2	1
Mb3488c	31794635	15 kDa	1	2.7	2.1	1.9	2	2.4
Mb1616	31792776	9 kDa	1	0.8	1.1	1.1	1.3	1
Mb1822	31792982	32 kDa	1	1.1	0.8	1	0.8	0.6

Mb3803	31794947	29 kDa	1.1	1	0.9	1.6	1.8	1.3
Mb2905c	31794057	32 kDa	1	0.6	1.2	0.6	0.6	0.6
Mb2617c	31793770	47 kDa	1	0.8	0.6	0.8	1.1	0.7
Mb3645c	31794791	11 kDa	1	0.9	0.8	0.9	0.4	0.9
Mb0207c	31791379	19 kDa	1	1.1	1.1	1.1	1.1	1
Mb1362c	31792523	79 kDa	1	0.8	0.6	0.7	0.8	0.7
Mb0723	31791888	11 kDa	1	1	1.1	0.9	1.1	0.9
Mb0724	31791889	31 kDa	1	2.1	1.9	1.6	1.6	1.7
Mb2979c	31794131	36 kDa	1	0.7	0.6	1.6	0.5	0.5
Mb3519	31794665	6 kDa	1	2.5	2.6	1.4	1.4	1.2
Mb1529	31792688	65 kDa	1	0.9	1.1	1.1	0.9	1
Mb0484	31791654	15 kDa	1	1.1	1.2	1.5	1.1	1.2
Mb0867c	31792032	23 kDa	1	1.6	1.1	1.4	1.7	1.1
Mb2204c	31793360	27 kDa	1	0.9	1.2	1	0.8	0.8
Mb2715c	31793868	27 kDa	1	1	0.9	1.3	0.9	0.9
Mb2970c	31794122	24 kDa	1	1.4	1	1.6	1.1	0.9
Mb3829c	31794973	57 kDa	1	0.8	0.7	0.8	0.9	0.7
Mb3309	31794461	17 kDa	1	0.7	1.1	0.9	0.7	1
Mb3909c	31795053	76 kDa	1	0.1	0.1	0.1	0.1	0.1
Mb1252c	31792413	22 kDa	1	1.2	1	1.4	0.9	1
Mb3320	31794472	45 kDa	1	1.1	1.9	1.4	1.2	0.9
Mb2996	31794148	30 kDa	1	0.9	1.2	1.2	1.1	1
Mb3715	31794861	24 kDa	1	0.8	0.4	0.9	0.8	0.8
Mb0415	31791585	37 kDa	1	0.6	0.8	0.6	0.7	0.8
Mb0438	31791608	12 kDa	1	1.4	1.7	1.6	1.6	1.2
Mb0752c	31791917	35 kDa	1	0.8	0.7	0.6	0.7	0.8
Mb0701	31791866	14 kDa	1	1.9	1.7	1.8	2.1	2.3
Mb0707	31791872	43 kDa	1	0.9	0.8	1.1	1	1
Mb3236c	31794388	25 kDa	1	1	0.8	2	0.9	0.8
Mb3639c	31794785	22 kDa	1	0.4	1.3	1.2	1.4	1.1
Mb0821c	31791986	29 kDa	1	0.8	0.8	0.8	0.8	0.9
Mb2089	31793246	8 kDa	1	0.9	1.5	0.9	1.2	1
Mb2190c	31793346	16 kDa	1	0.8	1.2	1.3	0.9	1.1
Mb1241	31792402	13 kDa	1	1	0.6	1	0.7	0.7
Mb2733	31793886	36 kDa	1	0.9	1.5	1.8	0.8	1.1
Mb0650c	31791815	32 kDa	1	0.9	1.6	1.1	1.3	1.2
Mb2301	31793457	48 kDa	1	1	1	1.3	1.1	0.8
Mb3301	31794453	81 kDa	1	0.8	0.9	0.4	1	3.1
Mb3514	31794660	54 kDa	0.7	1.8	2.4	2.1	0.1	0.2
Mb2475c	31793629	98 kDa	1	0.9	1	0.8	1	0.7
Mb2118c	31793274	26 kDa	1	0.7	0.7	1.3	0.7	0.8
Mb0501	31791671	25 kDa	1	0.8	0.8	0.7	0.8	0.4

Mb1776	31792935	92 kDa	1	0.9	0.8	1.1	0.9	0.9
Mb3207c	31794359	17 kDa	1	0.9	1.1	0.9	1.1	1
Mb3240	31794392	22 kDa	1	1.1	1	0.9	0.8	0.9
Mb1279c	31792440	10 kDa	1	0.9	1	1.3	0.9	0.9
Mb3888c	31795032	53 kDa	1	1.3	1.3	1.1	1.4	1.6
Mb3171	31794323	27 kDa	1	1.7	1.6	1.8	1.6	1.7
Mb1803	31792963	48 kDa	1	4.3	0.7	0.8	0.8	0.5
Mb2188c	31793344	40 kDa	1	1.5	1.4	1	0.7	1.3
Mb3035c	31794187	37 kDa	1	1.3	1.2	0.9	0.8	0.9
Mb0084	31791259	12 kDa	1	1.4	1.4	1	0.8	1.2
Mb1326	31792487	46 kDa	1.1	0.9	1.2	0.8	1.4	1.5
Mb3908	31795052	27 kDa	1.7	0.4	0.4	0.4	1.4	3.3
Mb2958	31794110	231 kDa	1	1.6	1.7	1.9	1.6	2.8
Mb3243c	31794395	14 kDa	1	1.2	1.7	1.2	1.3	1.2
Mb1706	31792865	38 kDa	1	1	1	1.2	1.2	1
Mb0015c	31791192	46 kDa	1	0.9	0.8	1.2	0.8	0.8
Mb2354	31793510	17 kDa	1	0.9	1.5	1.1	1	1.1
Mb0112c	31791286	8 kDa	1	0.3	1.4	1.1	1.5	1.3
Mb3864c	31795008	45 kDa	1	0.9	1.1	0.8	0.8	0.9
Mb0593c	31791760	106 kDa	1	0.9	1.1	0.8	1	1.1
Mb2386c	31793542	11 kDa	1	1.4	1.1	1.4	1.1	1.1
Mb3285c	31794437	49 kDa	1	0.8	0.7	1	0.9	1
Mb3037c	31794189	11 kDa	1	0.9	1.1	0.7	1.1	1
Mb3312	31794464	15 kDa	1	1.8	1	1.3	1.2	1.2
Mb3707	31794853	85 kDa	0.9	1.7	1.1	1.1	0.6	0.7
Mb0803	31791968	33 kDa	1	1.2	1.3	1.6	1	1.2
Mb2488c	31793642	22 kDa	1	1.8	0.8	1.3	0.6	0.8
Mb2932c	31794084	9 kDa	1	0.9	1.6	1	0.9	1.1
Mb1586	31792745	8 kDa	1	0.6	1.3	0.8	1.6	1.1
Mb1472	31792631	43 kDa	1	1.8	1.6	0.9	1.7	1.9
Mb1021	31792185	44 kDa	1	0.4	0.5	0.5	0.6	0.6
Mb1879	31793038	11 kDa	1	0.8	0.8	0.7	1	0.8
Mb2200c	31793356	51 kDa	1	1.1	1.1	1.3	1.1	1.3
Mb1306	31792467	19 kDa	1.2	1.3	0.9	1.8	1.5	2
Mb3839c	31794983	46 kDa	1	1.1	1.2	1.3	1.2	1.4
Mb1573	31792732	15 kDa	1	1.1	1.2	1.1	1.5	1.1
Mb2398c	31793554	8 kDa	1.1	1.1	2.5	1.3	1.2	1.7
Mb1643	31792803	51 kDa	1	0.9	0.8	0.8	1.1	0.9
Mb3441	31794588	11 kDa	1	0.8	1.1	1.1	1.3	1.1
Mb2646c	31793799	77 kDa	1	1	1	0.9	1	1
Mb1412c	31792571	23 kDa	1	1	1.1	1	1.2	1
Mb0654	31791819	17 kDa	1	1	0.9	0.9	1	0.9

Mb2633	31793786	10 kDa	1.1	1.3	1.6	1.7	1.3	1.6
Mb3077c	31794229	79 kDa	1	2.7	3	2.7	2.8	2.5
Mb1506	31792665	13 kDa	0.9	0.9	0.9	0.7	0.6	0.7
Mb2639	31793792	25 kDa	0.9	0.8	1.1	1.9	0.8	0.6
Mb0577	31791744	36 kDa	1	0.9	0.8	1.1	0.9	0.9
Mb0180	31791352	54 kDa	1	1.2	0.7	1.5	0.7	0.9
Mb2564c	31793717	39 kDa	1	1.5	1	2	1.2	0.9
Mb2492c	31793646	17 kDa	1	1.2	0.9	1.1	1	1.1
Mb1887c	31793046	23 kDa	1.1	3.1	0.9	0.9	2.3	3.2
Mb1683	31792842	41 kDa	1	0.9	0.9	0.6	1	1.1
Mb0217	31791389	67 kDa	1	3.2	2	1.8	1.6	2.3
Mb3362	31794514	48 kDa	1	0.5	0.5	0.5	1.1	0.7
Mb1737	31792897	25 kDa	1	1.8	1.3	2.2	1.3	1.7
Mb3434	31794582	87 kDa	1	1	0.9	1.2	0.6	0.6
Mb0179	31791351	42 kDa	1	0.8	0.7	1.1	0.8	0.8
Mb2225c	31793381	34 kDa	1	0.6	1.8	1.1	1.6	1.1
Mb1225	31792386	50 kDa	1	0.8	0.6	0.6	0.9	1
Mb0877	31792042	16 kDa	1	0.3	0.4	0.4	0.9	0.7
Mb1478c	31792637	18 kDa	1	0.8	0.9	0.6	0.9	1
Mb0539	31791708	23 kDa	1	3.1	2.1	1.4	0.9	1.1
Mb3074c	31794226	37 kDa	1	1.1	1.6	2.1	1.5	1.3
Mb1800	31792960	48 kDa	1	0.4	0.7	0.7	0.7	0.8
Mb3295	31794447	51 kDa	1	1.1	1	0.9	1.4	1.1
Mb0574c	31791741	12 kDa	1	1.7	1.8	1.2	1.3	1.3
Mb0204c	31791376	74 kDa	1	1.4	1.2	1.2	1.2	1.1
Mb0367c	31791537	15 kDa	1.1	0.8	0.8	1.1	1.9	1.3
Mb0738	31791903	7 kDa	1	2.3	1.9	1.5	1.7	2.1
Mb0316	31791487	26 kDa	1	1.5	1.8	1.9	1.6	1.2
Mb2991c	31794143	120 kDa	1	0.5	0.3	0.5	0.4	0.4
Mb1011c	31792175	16 kDa	1.4	0.3	0.3	0.3	2	3.1
Mb2178c	31793334	56 kDa	0.9	0.6	1.1	0.5	0.8	0.7
Mb0257c	31791429	18 kDa	1	1.3	9.2	1	1.8	1.4
Mb2221c	31793377	31 kDa	1	1.1	0.7	1.2	0.9	0.8
Mb2559Ac	31793712	8 kDa	1	0.7	1	0.6	1.1	1
Mb2890	31794042	10 kDa	1	0.8	0.9	0.7	0.9	0.9
Mb2234c	31793390	40 kDa	1	0.8	0.8	0.9	0.8	0.8
Mb2480c	31793634	21 kDa	1	1	1.1	1.2	1.1	0.8
Mb3473c	31794620	16 kDa	1	1.1	1	1.4	1.1	0.9
Mb3080c	31794232	19 kDa	1	1.2	1.6	1.8	0.8	1.3
Mb3846c	31794990	28 kDa	1	0.8	0.9	1	0.9	1.3
Mb3432	31794580	38 kDa	1	1.2	1.2	1.4	1	1.1
Mb3117	31794269	32 kDa	1	0.9	1.1	1.3	1.2	0.6

Mb3020c	31794172	55 kDa	0.9	1.1	1.1	1.2	0.6	0.6
Mb2451c	31793607	44 kDa	1	1	1.3	1	1.1	0.7
Mb2237c	31793393	64 kDa	1	1.1	1.3	1.2	0.8	1
Mb0882	31792047	42 kDa	1.1	0.6	0.6	0.7	1	1.3
Mb0689	31791854	27 kDa	1	1.5	1.6	1.4	1	1.1
Mb3126c	31794278	31 kDa	1	0.7	1	1	0.8	0.8
Mb3736c	31794881	44 kDa	1	1.1	1.3	1.9	0.8	1.1
Mb2870c	31794022	63 kDa	1	1.2	0.8	0.9	0.8	0.8
Mb0516c	31791686	18 kDa	0.9	0.9	1	1.5	0.6	0.6
Mb2585c	31793738	97 kDa	1	1.3	0.8	0.7	1.1	0.9
Mb1685	31792844	17 kDa	1	1.8	1.4	1.3	1.1	1.1
Mb2702	31793855	18 kDa	1	1.7	0.8	2	0.8	1.2
Mb1034c	31792198	58 kDa	1	0.9	0.8	0.8	0.9	0.8
Mb2713c	31793866	14 kDa	1.1	0.4	1.4	0.9	1.2	1.6
Mb0389c	31791559	19 kDa	1	1.1	1.1	1.3	1.3	1.1
Mb1049	31792212	35 kDa	1	1.2	1.3	1.4	1.4	1
Mb0624	31791790	9 kDa	1	0.9	1.3	0.8	1.2	0.9
Mb1943c	31793101	81 kDa	1	0.6	0.6	0.7	0.8	0.8
Mb0396	31791566	15 kDa	1	0.9	1.1	1.4	1	1.2
Mb3810	31794954	30 kDa	1	1.5	1.1	1.8	1.4	1
Mb1513	31792672	50 kDa	1	1	1	0.6	1.2	0.8
Mb1842c	31793001	42 kDa	1	1.4	0.9	1.3	1.2	1.1
Mb2943c	31794095	12 kDa	1.1	1.2	1	1.3	2.1	1.4
Mb0232c	31791404	46 kDa	1	1.3	0.9	1.4	0.7	0.9
Mb0511	31791681	30 kDa	1	1.1	0.9	1.4	0.8	0.8
Mb2443c	31793599	14 kDa	1	1.1	0.7	1.1	0.8	0.9
Mb1710A	31792870	8 kDa	1	1.3	2.7	1	0.9	1.3
Mb3065c	31794217	27 kDa	1	1.1	1	1.3	1	1
Mb2756c	31793909	85 kDa	1	1.6	2.2	1.8	2.1	4.1
Mb2164c	31793320	19 kDa	1	0.9	1.1	1.3	0.9	0.8
Mb2484c	31793638	47 kDa	1	0.8	1	1.1	0.8	0.8
Mb1233c	31792394	33 kDa	1	1.1	1.1	1.7	0.7	0.9
Mb2497	31793651	15 kDa	1	1.3	0.8	1.3	0.9	0.9
Mb3132c	31794284	42 kDa	1	1.4	1.2	1.7	0.9	1
Mb2139c	31793295	67 kDa	1	1.2	1.5	1.1	1.3	1.1
Mb3747	31794892	47 kDa	1	1.4	1.2	1.1	1	1
Mb2794c	31793947	17 kDa	1	1.1	0.7	1.1	1.2	0.8
Mb3940	31795083	124 kDa	1	1.3	1.3	1.6	1.4	1.1
Mb3695c	31794841	41 kDa	1	1.7	0.7	1.5	1	0.8
Mb3623	31794769	11 kDa	1	0.9	1	1.2	1.1	0.9
Mb2269	31793425	43 kDa	1	1.9	1.7	2.1	1.6	2.3
Mb1124	31792287	31 kDa	1	2.1	4.3	1.4	3.5	2.1

Mb0596	31791763	8 kDa	1	0.6	0.9	0.8	0.7	0.8
Mb0018c	31791195	54 kDa	1	0.8	1.6	1.7	1.5	1.4
Mb2316	31793472	44 kDa	1	0.6	0.7	0.7	0.8	0.8
Mb0536c	31791705	15 kDa	1	0.3	0.3	0.3	0.9	0.7
Mb1458	31792617	35 kDa	1	0.8	0.8	1.2	0.8	0.6
Mb2950c	31794102	25 kDa	1	2.5	1.9	1.8	0.6	0.7
Mb2656	31793809	32 kDa	1	0.4	0.9	0.3	0.8	1.4
Mb0474c	31791644	53 kDa	0.8	1.4	2.5	1.2	0.4	0.3
Mb0166	31791339	47 kDa	1	1.6	1.5	1.3	1.4	1.6
Mb0426	31791596	52 kDa	1	0.9	1.4	1.2	1.1	1.1
Mb0181	31791353	22 kDa	1	0.9	1	1.8	1.1	0.9
Mb2539	31793691	23 kDa	1	1.8	1.1	1.9	1	1
Mb0182	31791354	35 kDa	1	1	1.1	1.1	1.2	1.2
Mb3017c	31794169	25 kDa	1	1.7	1.1	1.5	1	1
Mb0190	31791362	27 kDa	1	2.3	2.3	1.7	2.6	1.8
Mb2138	31793294	23 kDa	1	1	0.9	1.2	1	1
Mb0311	31791482	33 kDa	1	0.5	0.5	0.5	0.6	0.5
Mb0397	31791567	43 kDa	1	0.8	0.8	0.9	1.2	1.1
Mb0473c	31791643	21 kDa	1	0.8	0.8	0.8	0.8	0.9
Mb0499	31791669	27 kDa	1	0.8	1.1	0.5	1	1
Mb0507	31791677	35 kDa	1	0.7	0.9	0.9	0.9	1
Mb0652	31791817	9 kDa	1	0.8	1.1	1	0.8	0.7
Mb3173	31794325	27 kDa	1	2.1	2.3	2	1.9	1.7
Mb1100c	31792262	36 kDa	1	0.9	0.9	1.1	0.9	0.9
Mb2217	31793373	29 kDa	1	1	0.9	1.1	0.9	0.9
Mb1250c	31792411	33 kDa	1	0.7	0.6	0.9	0.8	1.2
Mb0740	31791905	19 kDa	1	2.1	1.7	1.8	1.6	2.1
Mb1499	31792658	45 kDa	1	1	1.1	1.2	1.1	1.1
Mb3239c	31794391	29 kDa	1	3.4	2.9	1.3	2	1.8
Mb1530	31792689	81 kDa	1	0.8	0.7	1.1	0.9	0.9
Mb0578	31791745	31 kDa	1	1	0.9	0.9	1.1	1.6
Mb2907c	31794059	27 kDa	1	1.3	0.9	1.3	0.9	0.9
Mb0028	31791204	12 kDa	1	0.4	0.9	1.5	1.2	1
Mb1863	31793022	99 kDa	1	0.9	1	0.9	0.8	0.9
Mb1875c	31793034	74 kDa	1	1.7	1	2.2	1.2	1.7
Mb0023	31791200	27 kDa	1	0.8	0.7	0.5	0.5	0.5
Mb2194c	31793350	33 kDa	1	1.5	1.1	1.7	2.5	1.5
Mb2246c	31793402	50 kDa	1	0.7	0.7	1	0.8	0.8
Mb2282c	31793438	38 kDa	1	0.7	0.7	0.7	0.7	0.6
Mb2623c	31793776	37 kDa	1	1.1	1.3	1.5	1.2	1.6
Mb2695c	31793848	26 kDa	1	1	1.1	1.2	1.2	1.2
Mb2956	31794108	199 kDa	1	1.6	1.7	1.6	1	1.9

Mb2912c	31794064	51 kDa	1	0.9	0.9	1.1	0.9	0.9
Mb1509	31792668	7 kDa	1	0.3	0.6	0.6	0.5	0.3
Mb3223	31794375	9 kDa	1	1.2	1.1	1.2	1.5	1.3
Mb1301c	31792462	25 kDa	1	0.3	0.3	0.4	0.4	0.5
Mb3225c	31794377	38 kDa	1	0.8	0.8	0.8	0.8	1
Mb0374c	31791544	14 kDa	1	0.7	2.1	1.4	1.6	1.4
Mb3256	31794408	46 kDa	1	1.1	0.9	0.9	0.9	1.1
Mb1636	31792796	25 kDa	1	1.1	0.9	1.1	0.9	0.7
Mb3283c	31794435	43 kDa	1	0.8	0.9	0.9	0.9	1.1
Mb2219	31793375	61 kDa	1	0.8	1	0.9	2	2.5
Mb3311	31794463	33 kDa	1	1.1	1	1.4	0.9	1
Mb1300c	31792461	13 kDa	1	0.7	0.4	0.6	0.6	0.8
Mb3348	31794500	29 kDa	1	0.9	1	1	0.9	3.2
Mb3424c	31794572	32 kDa	1	0.6	0.6	0.4	0.9	0.8
Mb2863c	31794015	19 kDa	1	2	1.8	2.2	2	2
Mb3369c	31794521	36 kDa	1	1.2	1	1.1	1	0.9
Mb3466c	31794613	68 kDa	1	0.9	1	0.9	0.8	0.8
Mb2236	31793392	53 kDa	1	0.8	0.8	1	0.7	0.8
Mb3515c	31794661	33 kDa	1	0.8	0.9	1	0.9	1
Mb3019c	31794171	35 kDa	1	1.3	1.4	1.5	1.1	1.1
Mb3329c	31794481	25 kDa	1	1.1	1.1	0.9	1.4	0.9
Mb3820	31794964	27 kDa	1	1.3	1.3	1	1.2	1.3
Mb1046c	31792209	52 kDa	1	0.5	0.6	0.6	0.6	0.9
Mb0547c	31791715	35 kDa	1	0.8	0.9	1.1	1.1	1.1
Mb0955c	31792119	31 kDa	1	0.6	0.6	0.7	0.6	0.7
Mb0300	31791471	36 kDa	1	0.8	1	0.9	0.9	1
Mb3372	31794524	47 kDa	1	0.6	0.5	0.5	0.3	4.1
Mb2181c	31793337	52 kDa	1	0.9	0.8	0.9	0.8	1
Mb3806	31794950	34 kDa	1	0.5	0.5	0.4	0.6	0.6
Mb0364c	31791534	47 kDa	1	0.7	0.7	0.7	0.9	0.8
Mb1680	31792839	36 kDa	1	0.9	1.1	1	0.9	1.1
Mb2911	31794063	15 kDa	1	2	1.2	1.8	1	1.6
Mb2271	31793427	50 kDa	1	0.6	1.8	1.1	0.8	1.6
Mb1954c	31793111	17 kDa	1	1.1	0.7	1.2	0.9	0.8
Mb2087c	31793244	15 kDa	1	1	0.9	1.3	0.8	0.8
Mb1664	31792824	106 kDa	1	1.3	1.3	1.5	1.4	1.8
Mb1512	31792671	20 kDa	1	0.7	1.7	1.3	1.3	1.2
Mb2021c	31793178	27 kDa	1	1.3	1.3	1.5	1.4	1.3
Mb1450	31792609	46 kDa	1	0.8	0.9	0.8	0.7	1.1
Mb0538	31791707	22 kDa	1	1.1	0.6	1.1	0.7	0.7
Mb1373	31792534	29 kDa	1	1	1.3	1.2	1	1
Mb1593c	31792752	24 kDa	1	0.9	0.5	0.5	0.6	0.6

Mb3520	31794666	56 kDa	1	1	1	1.2	0.9	0.9
Mb2109	31793266	31 kDa	1	2	1	1.6	0.8	0.8
Mb3493	31794640	38 kDa	1	0.8	1.1	1	0.9	1.2
Mb2233c	31793389	40 kDa	1	1.1	2.4	1.3	1.6	1.3
Mb0418c	31791588	82 kDa	1	0.4	0.7	0.4	0.7	0.9
Mb0524	31791693	59 kDa	1	2.8	1.6	1.2	2.6	2.2
Mb2788c	31793941	27 kDa	1	0.4	0.3	0.5	0.5	0.4
Mb0847c	31792012	39 kDa	1	1.8	2.8	1.7	2.6	2.1
Mb3039c	31794191	75 kDa	1	0.6	0.7	0.7	0.8	0.8
Mb1358	31792519	40 kDa	1	2.7	2.4	3.8	2.3	2.5
Mb0691	31791856	59 kDa	1	1.1	1	1.4	1.2	1.5
Mb2779c	31793932	9 kDa	1	1	1.2	0.8	1.5	1
Mb3076c	31794228	28 kDa	1	1.1	1.7	1.4	1.6	1
Mb3011c	31794163	22 kDa	1	1.1	1.4	1.2	1	1.7
Mb2665c	31793818	10 kDa	1	0.4	0.4	0.3	0.5	0.4
Mb1918c	31793077	35 kDa	1	2.1	1.8	1.5	1.7	1.9
Mb3292c	31794444	38 kDa	1	0.8	1	1.1	1	1.2
Mb0239c	31791411	54 kDa	1	1	1	1.6	1.7	1.6
Mb0005	31791182	78 kDa	1	1.4	1.8	2.3	1.2	1.7
Mb2155c	31793311	28 kDa	1	0.7	0.9	0.9	0.8	0.7
Mb2561c	31793714	14 kDa	1	1.3	1	1.1	1	0.9
Mb0042	31791218	108 kDa	1	1	1.1	1.3	1.2	1.1
Mb3272c	31794424	61 kDa	1	1.6	1.3	1.7	1.4	1.4
Mb0841	31792006	28 kDa	1	0.9	1	1.4	0.7	1.1
Mb1045c	31792208	35 kDa	1	1.4	1.6	1.7	1.3	1.1
Mb0134c	31791308	37 kDa	1	1.9	1.3	1.8	1.4	1.4
Mb3709	31794855	38 kDa	1	0.7	0.6	0.5	0.8	0.6
Mb2124c	31793280	51 kDa	1	1	1.1	1	1.1	1.1
Mb3685	31794831	31 kDa	1	1.8	2.2	1.7	1.9	1.4
Mb3471c	31794618	46 kDa	1	1.5	1.7	0.8	2	2.3
Mb0925	31792089	19 kDa	1	0.8	0.8	0.5	0.8	0.7
Mb0939c	31792103	41 kDa	1	1.3	2	1.6	1.6	1.5
Mb2130c	31793286	9 kDa	1	1	1.4	0.7	1.1	1
Mb1305	31792466	20 kDa	1	0.8	0.7	1	0.9	1
Mb0703	31791868	77 kDa	1	1.4	1.3	1.3	1.4	1.7
Mb3523c	31794669	26 kDa	1	0.9	0.9	1.3	1.1	0.8
Mb1677	31792837	88 kDa	1	0.9	1.3	0.8	0.9	0.8
Mb0356	31791526	24 kDa	1	1.3	1.1	1.4	1.3	1
Mb3005c	31794157	39 kDa	1	0.8	0.6	1.1	0.9	1.1
Mb3830c	31794974	186 kDa	1	0.9	0.6	0.8	0.9	1.1
Mb2972c	31794124	75 kDa	1	0.9	0.8	0.8	1.1	0.9
Mb0883	31792048	76 kDa	1	0.8	1	1.2	1	0.7

Mb0308	31791479	8 kDa	1	1	0.9	1	1.1	0.8
Mb0836c	31792001	24 kDa	1	0.6	0.9	2.4	2.5	1.6
Mb3944	31795086	36 kDa	1	0.3	1.5	0.6	1.2	0.7
Mb0463c	31791633	17 kDa	1	1.4	2.4	1.9	1.9	1.4
Mb3749c	31794894	47 kDa	1	1	0.9	1.4	1	0.7
Mb3619c	31794765	22 kDa	1	0.9	0.6	1.5	0.7	0.8
Mb1824	31792984	60 kDa	1	0.6	1.1	1.3	1.6	1.1
Mb2509c	31793663	52 kDa	1	0.5	0.6	0.4	0.7	0.4
Mb1571	31792730	28 kDa	1	0.7	0.8	1	0.7	1.9
Mb0739	31791904	14 kDa	1	2.3	1.6	1.5	1.7	2.1
Mb2659c	31793812	16 kDa	1	0.9	1.5	1.1	7.3	4.1
Mb2945c	31794097	44 kDa	1	2	1.2	1.8	1.6	1.8
Mb2493c	31793647	23 kDa	1	0.7	0.6	0.6	0.6	0.7
Mb0793	31791958	30 kDa	1	0.8	1.3	1.2	1.4	0.9
Mb3410	31794558	24 kDa	1	1.7	1.8	1.5	1.5	1.2
Mb1811	31792971	54 kDa	1	1	0.7	0.6	0.8	0.7
Mb2927c	31794079	32 kDa	1	2.5	2.3	1	1.8	1.5
Mb1520	31792679	29 kDa	1	2.1	1.7	1.1	1.8	1.6
Mb0289	31791460	33 kDa	1	0.8	1.3	1.3	1.7	1.4
Mb3524c	31794670	60 kDa	1	0.7	0.7	0.8	0.7	0.6
Mb1341	31792502	34 kDa	1	0.6	0.9	1.2	1.7	1.6
Mb1703	31792862	26 kDa	1	0.5	1.5	1.1	1.6	1
Mb1775	31792934	51 kDa	1	1.4	0.9	1.7	1	1.1
Mb2175c	31793331	34 kDa	1	1	0.9	1.2	1.1	0.8
Mb0051	31791227	71 kDa	1	2.5	1	1.3	1.8	1
Mb0869c	31792034	54 kDa	1	0.8	1.4	0.6	1.7	1
Mb3082	31794234	38 kDa	1	0.5	0.6	0.7	1.3	0.6
Mb2553c	31793705	326 kDa	1	0.5	0.6	0.9	0.6	0.8
Mb1860	31793019	18 kDa	1	0.8	0.7	1	0.8	0.8
Mb1596	31792755	40 kDa	1	0.3	0.9	0.8	0.9	0.8
Mb2855	31794007	27 kDa	1	0.8	2.5	1.8	2.3	1.2
Mb0993	31792157	10 kDa	1	0.4	1.8	1.7	2.1	1.2
Mb0340	31791511	13 kDa	1	1	0.8	1.3	0.9	1
Mb1837	31792997	40 kDa	1	1.7	0.7	2	0.8	0.8
Mb1452	31792611	16 kDa	1	1	1	0.9	1.2	0.8
Mb0242c	31791414	6 kDa	1	0.6	1.9	0.8	1.3	1.3
Mb0515c	31791685	35 kDa	1	0.6	0.6	1.5	0.6	1.1
Mb3237	31794389	57 kDa	1	0.8	1.1	1.6	1.2	1.2
Mb2975c	31794127	41 kDa	1	1.2	1.2	1.5	1	1.2
Mb2323	31793479	24 kDa	1	0.6	0.7	0.8	0.7	0.7
Mb1263c	31792424	20 kDa	1	0.8	0.6	0.8	0.5	0.5
Mb0641c	31791807	25 kDa	1	1.1	2.3	2.1	2.6	1.9

Mb1655	31792815	98 kDa	1	0.4	0.5	0.8	0.5	0.4
Mb3640c	31794786	82 kDa	1	1	1.3	1.6	1.6	1.3
Mb1877c	31793036	15 kDa	1	0.2	0.5	0.4	0.5	0.3
Mb2041	31793198	26 kDa	1	2	1.2	1.8	1.2	1.6
Mb0412	31791582	49 kDa	1	0.5	0.6	0.9	0.6	0.6
Mb1517	31792676	36 kDa	1	0.5	0.3	1.1	0.3	0.3
Mb2961	31794113	36 kDa	1	2.4	1.7	1.6	1.8	2.1
Mb0439	31791609	17 kDa	1	0.9	1.3	1.2	1.3	1
Mb3445c	31794592	55 kDa	1	0.3	3.7	0.2	0.7	0.2
Mb0222	31791394	36 kDa	1	0.6	1.3	0.7	0.7	0.6
Mb1443	31792602	24 kDa	1	1.7	1	1.8	1.2	1.5
Mb0948c	31792112	73 kDa	1	0.8	1.8	1.8	1.8	1.4
Mb3164	31794316	43 kDa	1	1.8	1.6	1.3	1.3	1.3
Mb0142c	31791315	20 kDa	1	1.3	1.5	1.4	1.1	1.3
Mb0276	31791448	60 kDa	1	0.3	0.5	0.4	0.4	0.3
Mb1809	31792969	20 kDa	1	1.3	0.9	2.4	1	0.9
Mb1851	31793010	58 kDa	1	0.6	0.8	1.2	1.5	0.8
Mb2626	31793779	9 kDa	1	1.3	1.8	1	1.8	1.3
Mb3742c	31794887	22 kDa	1	1.7	0.8	1.5	1	1.5
Mb1570	31792729	37 kDa	1	1.2	1.2	1.3	0.9	1.2
Mb0347	31791518	18 kDa	1	0.8	1	0.7	0.9	0.9
Mb1043c	31792206	22 kDa	1	1.1	1.3	1.5	1.6	1.7
Mb1385	31792546	26 kDa	1	0.6	0.6	0.7	0.6	0.5
Mb0076	31791251	43 kDa	1	0.7	1.1	1.1	1.5	1.3
Mb0970	31792134	27 kDa	1	0.7	1.2	0.6	1.3	0.8
Mb2883c	31794035	48 kDa	1	1.1	0.9	1.4	1	0.9
Mb2583c	31793736	45 kDa	1	1.4	0.6	1.4	0.9	0.9
Mb2362	31793518	33 kDa	1	1.1	1.4	0.8	1.4	0.9
Mb3916c	31795060	56 kDa	1	0.9	1	0.9	1.3	0.9
Mb2284	31793440	22 kDa	1	0.8	0.7	0.9	0.6	0.6
Mb3785c	31794929	33 kDa	1	1.1	0.8	1.9	1	1.2
Mb1137c	31792300	9 kDa	1	0.8	0.9	1.3	1.6	1.5
Mb0149	31791322	31 kDa	1	0.3	0.7	1.5	1.1	0.7
Mb2229	31793385	26 kDa	1	0.9	1.6	0.7	1.5	1.1
Mb0812	31791977	24 kDa	1	0.4	0.4	0.6	0.3	0.4
Mb0845c	31792010	73 kDa	1	1.4	1.6	1	1.1	0.9
Mb1266	31792427	19 kDa	1	0.5	0.4	0.7	0.4	0.3
Mb2213c	31793369	40 kDa	1	1.7	2	1.2	2	1
Mb2555	31793707	8 kDa	1	0.5	1.4	0.7	1.5	0.8
Mb3635c	31794781	17 kDa	1	2	1.2	1	1	0.6

Table S8: Primerpairs used for QPCR in this study

**Primer**	**Sequence**
esxI_Fw	TCCAGGTGATCTACGAGCAG
esxI_Rv	GTTTGTGCCATGTTGTTGC
Mb3440_Fw	CACCAACCGCTATGACTACG
Mb3440_Rv	CTCGAACACCTGACGGAAG
Mb0951_Fw	CTCGACACGGACTCGTTCTA
Mb0951_Rv	GCAAACGATTTCGTATGTCG
glpD2_Fw	TCGGTGATGTTTGTCATTCC
glpD2_Rv	GTCGAGGTTCCAGTCGGTAT
desA3_Fw	GAGGTCTGCGACAGATACGA
desA3_Rv	TACTTATCCGGCAACGACAG
Mb3614c_Fw	GAAGGCCTGGACAAGGTTT
Mb3614c_Rv	GATGCGAGTTTCTCGAGGTT
PPE40_Fw	TTGCAAACATTGGCTCTTTC
PPE40_Rv	TCTGGCTACCCGAATTTACC
PE_PGRS57_Fw	ATGAGGTGTCTGCCCGTATT
PE_PGRS57_Rv	GTACTCAAGGCCTGCACAAA
mtc28_Fw	GAGCCGACAAGTACCTGGTT
mtc28_Rv	ACCACTTGGAATCCGTTGAC
whiB1_Fw	AACAGTGGTCCGGCACTT
whiB1_Rv	GTCCTGGCCGGTATTCAGT
whiB6_Fw	AAGATCCCGATCGTTGGAC
whiB6_Rv	CCTGATTCGGGAATTACGAC
sigA_Fw	TGATTTCGTCTGGGATGAAG
sigA_Rv	TGCCGATCTGTTTGAGGTAG
PE13_Fw	GCGATGTATCAGTCCGTGAG
PE13_Rv	GACTTCAGTGGCCGCATAC
esxL_Fw	GATGTCGACGATCATGGC
esxL_Rv	AAAGTCACTCGCGGTCAAC

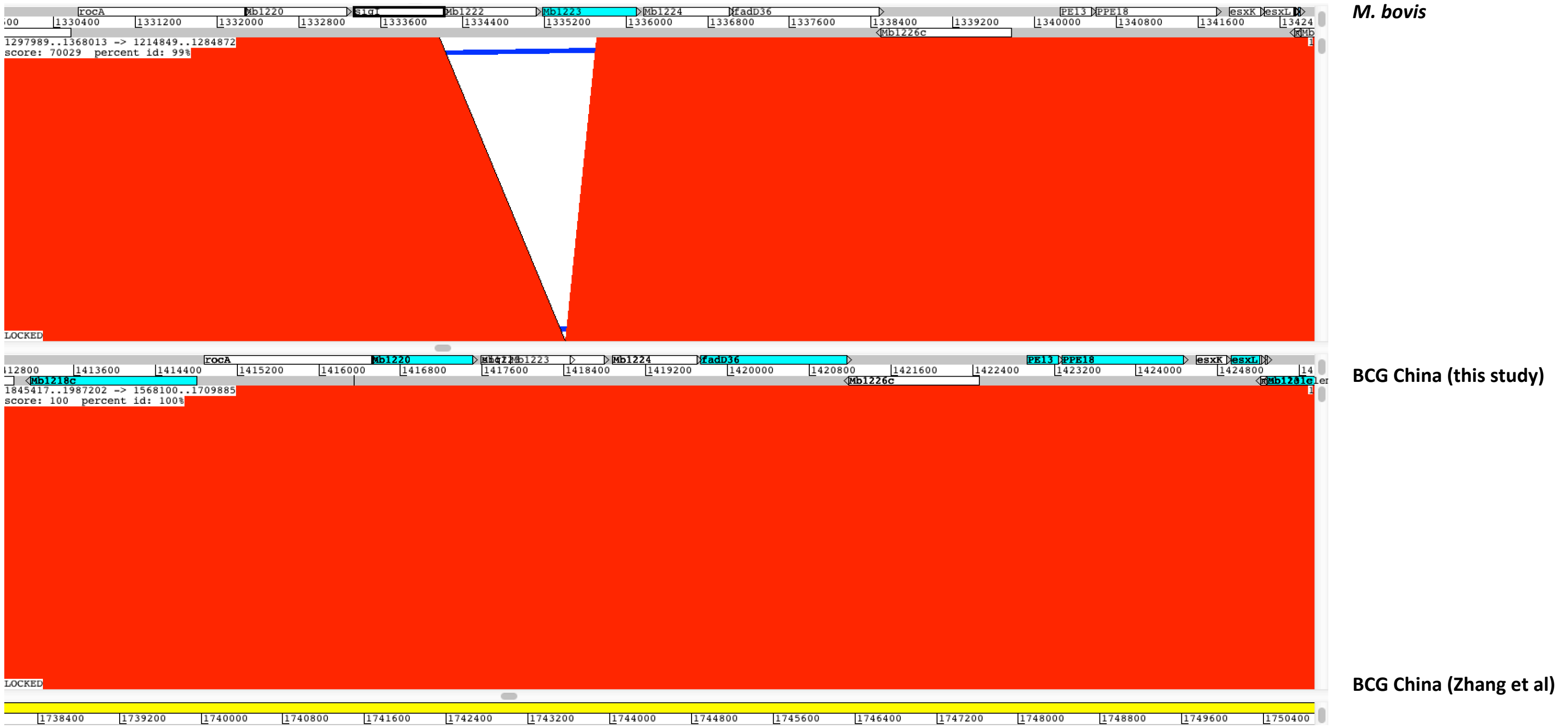

Figure S1

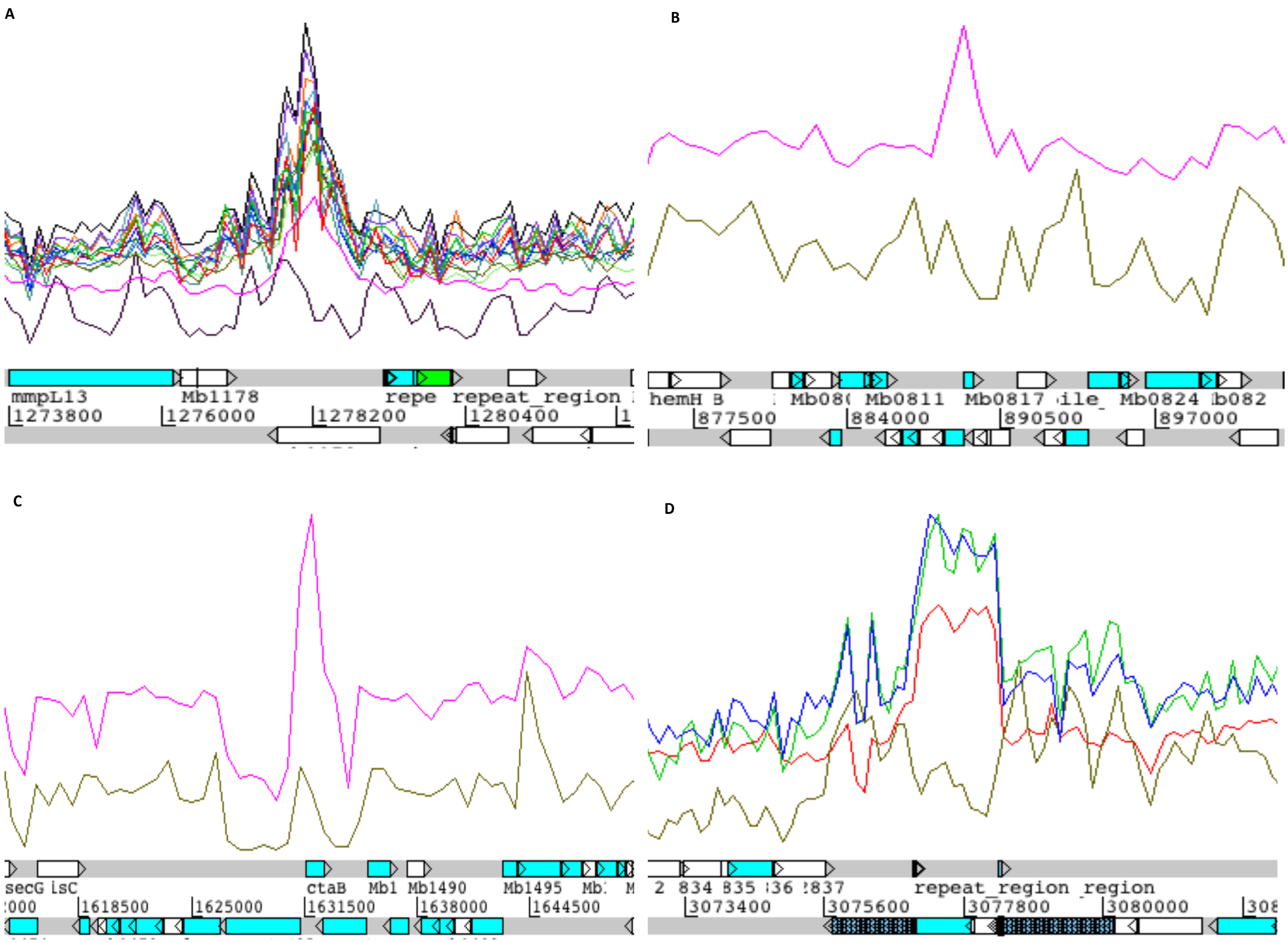
A
mmpL13
Mb1178
repe
repeat_region
1273800
1276000
1278200
1280400
B
hemH
Mb0811
Mb0817
Mb0824
877500
884000
890500
897000
C
secG
ctaB
Mb1490
Mb1495
1618500
1625000
1631500
1638000
1644500
D
repeat_region
3073400
3075600
3077800
3080000

Figure S2

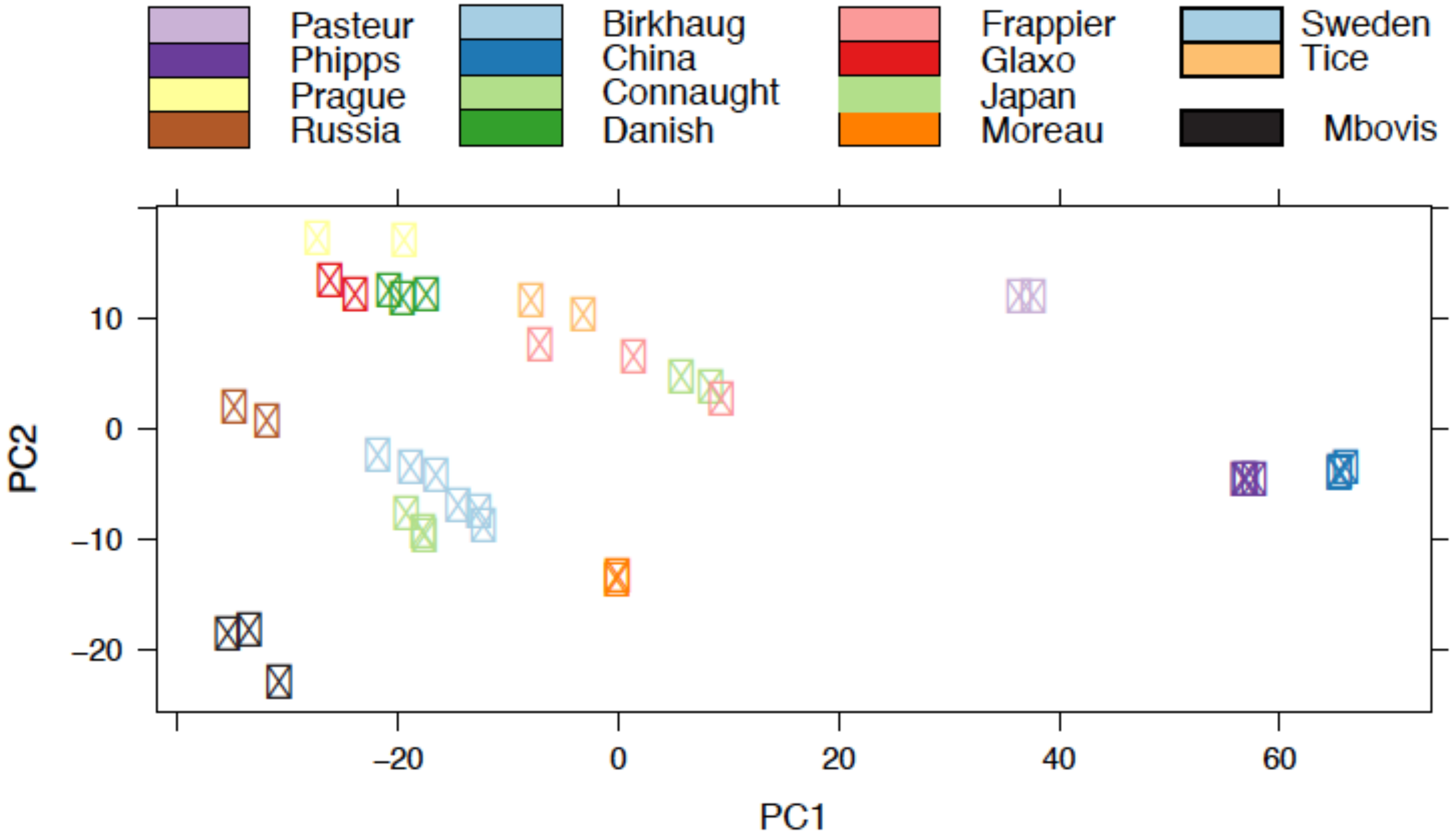
Pasteur
Phipps
Prague
Russia
Birkhaug
China
Connaught
Danish
Frappier
Glaxo
Japan
Moreau
Sweden
Tice
Mbovis
PC2
10
0
–10
–20
–20
0
20
40
60
PC1

Figure S3

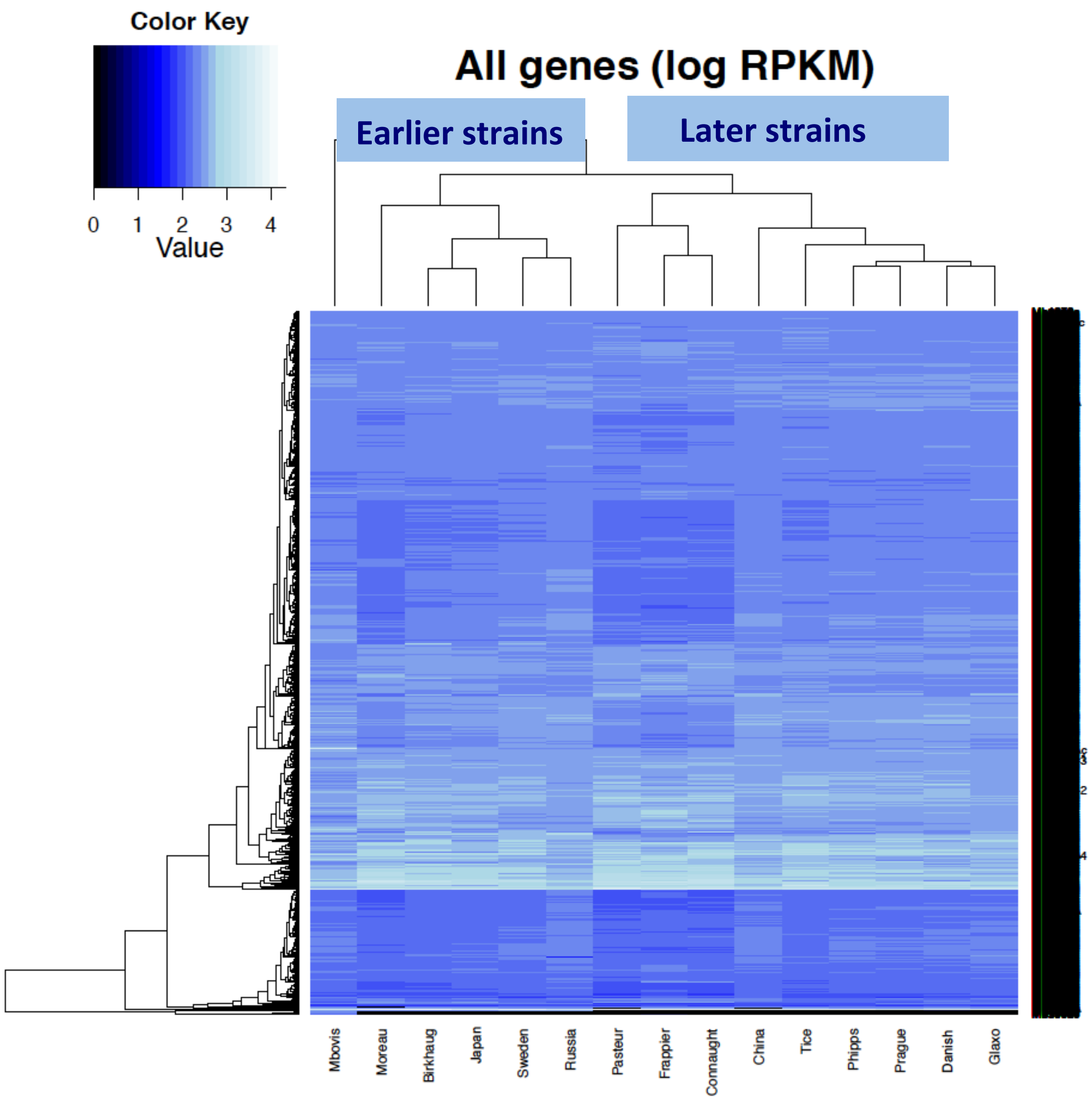
Color Key
0
1
2
3
4
Value
All genes (log RPKM)
Earlier strains
Later strains
Mbovis
Moreau
Birkhaug
Japan
Sweden
Russia
Pasteur
Frappier
Connaught
China
Tice
Phipps
Prague
Danish
Glaxo

Figure S4

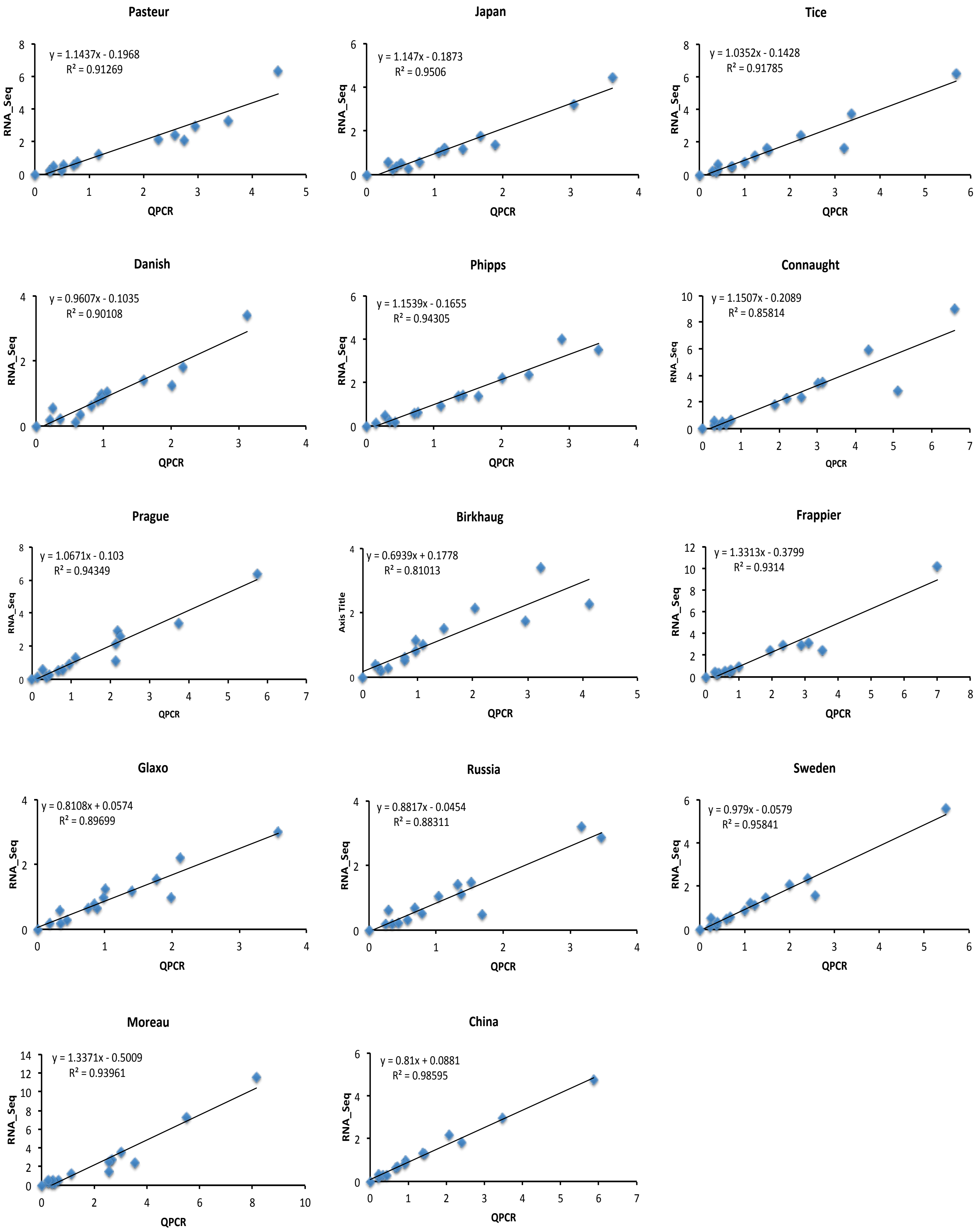
Pasteur
y = 1.1437x - 0.1968
R² = 0.91269
RNA_Seq
QPCR
Japan
y = 1.147x - 0.1873
R² = 0.9506
RNA_Seq
QPCR
Tice
y = 1.0352x - 0.1428
R² = 0.91785
RNA_Seq
QPCR
Danish
y = 0.9607x - 0.1035
R² = 0.90108
RNA_Seq
QPCR
Phipps
y = 1.1539x - 0.1655
R² = 0.94305
RNA_Seq
QPCR
Connaught
y = 1.1507x - 0.2089
R² = 0.85814
RNA_Seq
QPCR
Prague
y = 1.0671x - 0.103
R² = 0.94349
RNA_Seq
QPCR
Birkhaug
y = 0.6939x + 0.1778
R² = 0.81013
Axis Title
QPCR
Frappier
y = 1.3313x - 0.3799
R² = 0.9314
RNA_Seq
QPCR
Glaxo
y = 0.8108x + 0.0574
R² = 0.89699
RNA_Seq
QPCR
Russia
y = 0.8817x - 0.0454
R² = 0.88311
RNA_Seq
QPCR
Sweden
y = 0.979x - 0.0579
R² = 0.95841
RNA_Seq
QPCR
Moreau
y = 1.3371x - 0.5009
R² = 0.93961
RNA_Seq
QPCR
China
y = 0.81x + 0.0881
R² = 0.98595
RNA_Seq
QPCR

Figure S5

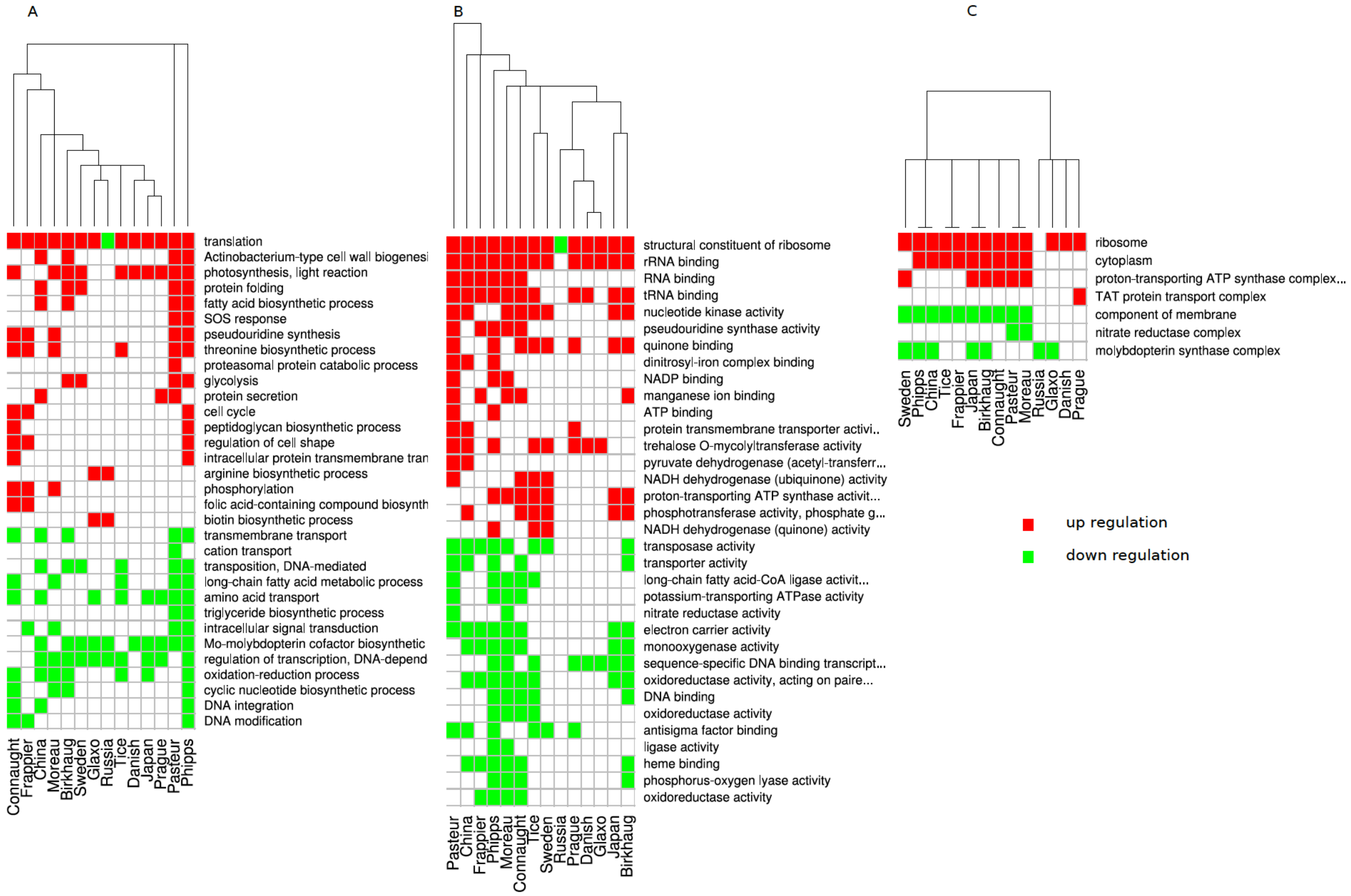
A
translation
Actinobacterium-type cell wall biogenesi
photosynthesis, light reaction
protein folding
fatty acid biosynthetic process
SOS response
pseudouridine synthesis
threonine biosynthetic process
proteasomal protein catabolic process
glycolysis
protein secretion
cell cycle
peptidoglycan biosynthetic process
regulation of cell shape
intracellular protein transmembrane tran
arginine biosynthetic process
phosphorylation
folic acid-containing compound biosynth
biotin biosynthetic process
transmembrane transport
cation transport
transposition, DNA-mediated
long-chain fatty acid metabolic process
amino acid transport
triglyceride biosynthetic process
intracellular signal transduction
Mo-molybdopterin cofactor biosynthetic
regulation of transcription, DNA-depend
oxidation-reduction process
cyclic nucleotide biosynthetic process
DNA integration
DNA modification
Connaught
Frappier
China
Moreau
Birkhaug
Sweden
Glaxo
Russia
Tice
Danish
Japan
Prague
Pasteur
Phipps
B
structural constituent of ribosome
rRNA binding
RNA binding
tRNA binding
nucleotide kinase activity
pseudouridine synthase activity
quinone binding
dinitrosyl-iron complex binding
NADP binding
manganese ion binding
ATP binding
protein transmembrane transporter activi..
trehalose O-mycolyltransferase activity
pyruvate dehydrogenase (acetyl-transferr...
NADH dehydrogenase (ubiquinone) activity
proton-transporting ATP synthase activit...
phosphotransferase activity, phosphate g...
NADH dehydrogenase (quinone) activity
transposase activity
transporter activity
long-chain fatty acid-CoA ligase activit...
potassium-transporting ATPase activity
nitrate reductase activity
electron carrier activity
monooxygenase activity
sequence-specific DNA binding transcript...
oxidoreductase activity, acting on paire...
DNA binding
oxidoreductase activity
antisigma factor binding
ligase activity
heme binding
phosphorus-oxygen lyase activity
oxidoreductase activity
Pasteur
China
Frappier
Phipps
Moreau
Connaught
Tice
Sweden
Russia
Prague
Danish
Glaxo
Japan
Birkhaug
C
ribosome
cytoplasm
proton-transporting ATP synthase complex...
TAT protein transport complex
component of membrane
nitrate reductase complex
molybdopterin synthase complex
Sweden
Phipps
China
Tice
Frappier
Japan
Birkhaug
Connaught
Pasteur
Moreau
Russia
Glaxo
Danish
Prague
up regulation
down regulation

Figure S6

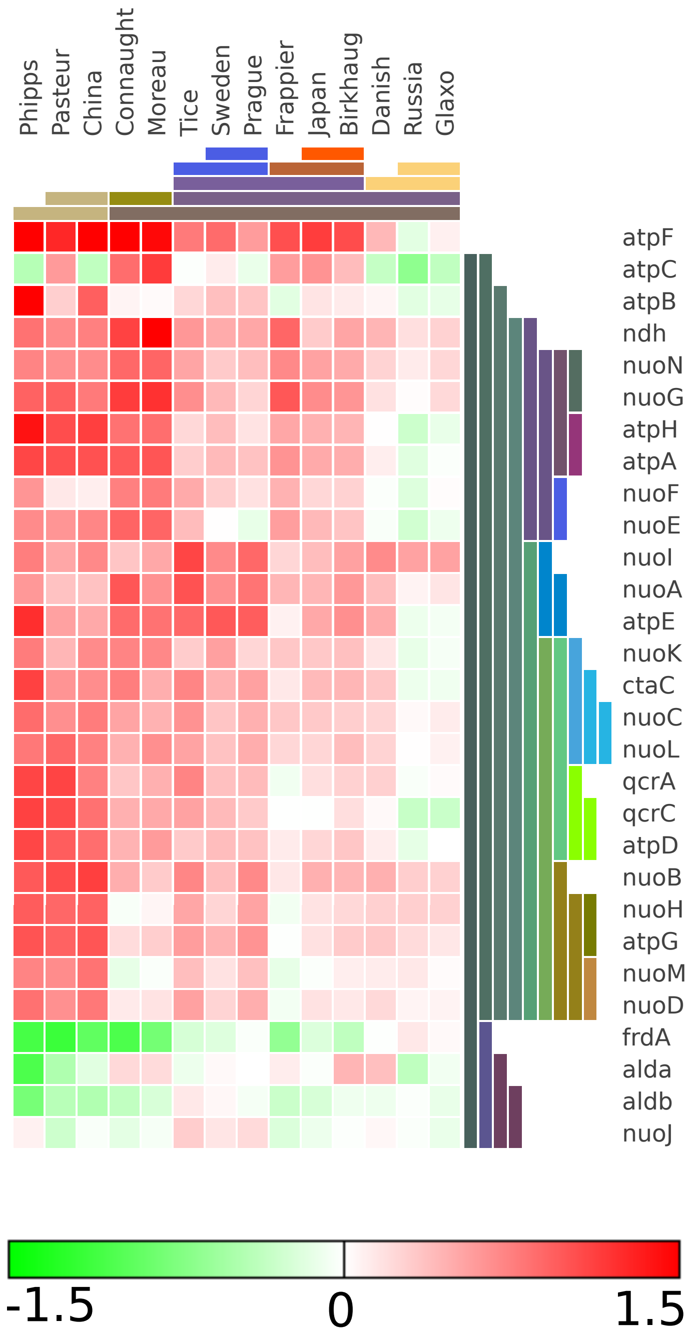
Phipps
Pasteur
China
Connaught
Moreau
Tice
Sweden
Prague
Frappier
Japan
Birkhaug
Danish
Russia
Glaxo
atpF
atpC
atpB
ndh
nuoN
nuoG
atpH
atpA
nuoF
nuoE
nuoI
nuoA
atpE
nuoK
ctaC
nuoC
nuoL
qcrA
qcrC
atpD
nuoB
nuoH
atpG
nuoM
nuoD
frdA
alda
aldb
nuoJ
-1.5
0
1.5

Figure S7

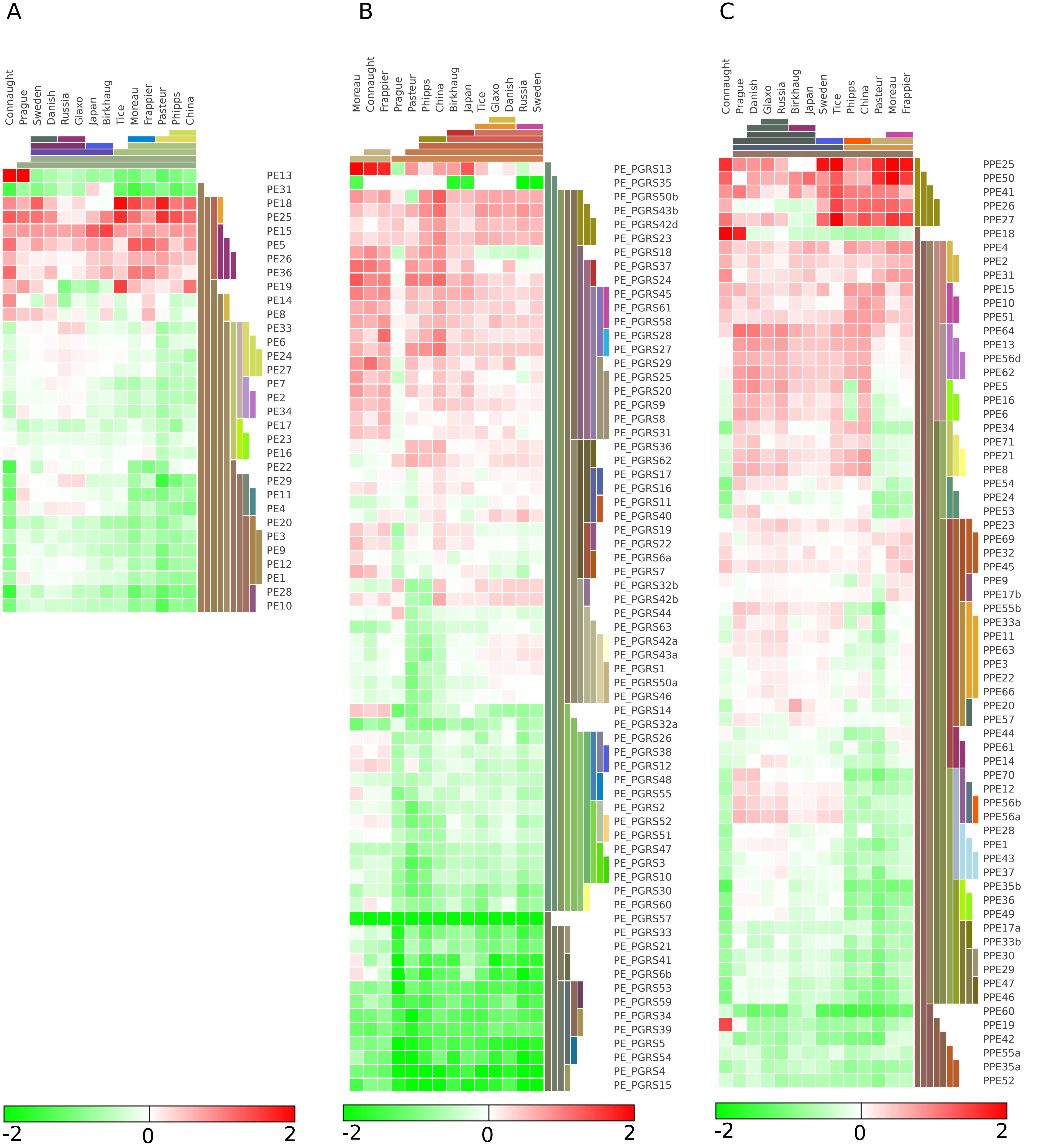

A
Connaught
Prague
Sweden
Danish
Russia
Glaxo
Japan
Birkhaug
Tice
Moreau
Frappier
Pasteur
Phipps
China
PE13
PE31
PE18
PE25
PE15
PE5
PE26
PE36
PE19
PE14
PE8
PE33
PE6
PE24
PE27
PE7
PE2
PE34
PE17
PE23
PE16
PE22
PE29
PE11
PE4
PE20
PE3
PE9
PE12
PE1
PE28
PE10
-2
0
2
B
Moreau
Connaught
Frappier
Prague
Pasteur
Phipps
China
Birkhaug
Japan
Tice
Glaxo
Danish
Russia
Sweden
PE_PGRS13
PE_PGRS35
PE_PGRS50b
PE_PGRS43b
PE_PGRS42d
PE_PGRS23
PE_PGRS18
PE_PGRS37
PE_PGRS24
PE_PGRS45
PE_PGRS61
PE_PGRS58
PE_PGRS28
PE_PGRS27
PE_PGRS29
PE_PGRS25
PE_PGRS20
PE_PGRS9
PE_PGRS8
PE_PGRS31
PE_PGRS36
PE_PGRS62
PE_PGRS17
PE_PGRS16
PE_PGRS11
PE_PGRS40
PE_PGRS19
PE_PGRS22
PE_PGRS6a
PE_PGRS7
PE_PGRS32b
PE_PGRS42b
PE_PGRS44
PE_PGRS63
PE_PGRS42a
PE_PGRS43a
PE_PGRS1
PE_PGRS50a
PE_PGRS46
PE_PGRS14
PE_PGRS32a
PE_PGRS26
PE_PGRS38
PE_PGRS12
PE_PGRS48
PE_PGRS55
PE_PGRS2
PE_PGRS52
PE_PGRS51
PE_PGRS47
PE_PGRS3
PE_PGRS10
PE_PGRS30
PE_PGRS60
PE_PGRS57
PE_PGRS33
PE_PGRS21
PE_PGRS41
PE_PGRS6b
PE_PGRS53
PE_PGRS59
PE_PGRS34
PE_PGRS39
PE_PGRS5
PE_PGRS54
PE_PGRS4
PE_PGRS15
-2
0
2
C
Connaught
Prague
Danish
Glaxo
Russia
Birkhaug
Japan
Sweden
Tice
Phipps
China
Pasteur
Moreau
Frappier
PPE25
PPE50
PPE41
PPE26
PPE27
PPE18
PPE4
PPE2
PPE31
PPE15
PPE10
PPE51
PPE64
PPE13
PPE56d
PPE62
PPE5
PPE16
PPE6
PPE34
PPE71
PPE21
PPE8
PPE54
PPE24
PPE53
PPE23
PPE69
PPE32
PPE45
PPE9
PPE17b
PPE55b
PPE33a
PPE11
PPE63
PPE3
PPE22
PPE66
PPE20
PPE57
PPE44
PPE61
PPE14
PPE70
PPE12
PPE56b
PPE56a
PPE28
PPE1
PPE43
PPE37
PPE35b
PPE36
PPE49
PPE17a
PPE33b
PPE30
PPE29
PPE47
PPE46
PPE60
PPE19
PPE42
PPE55a
PPE35a
PPE52
-2
0
2

Figure S8

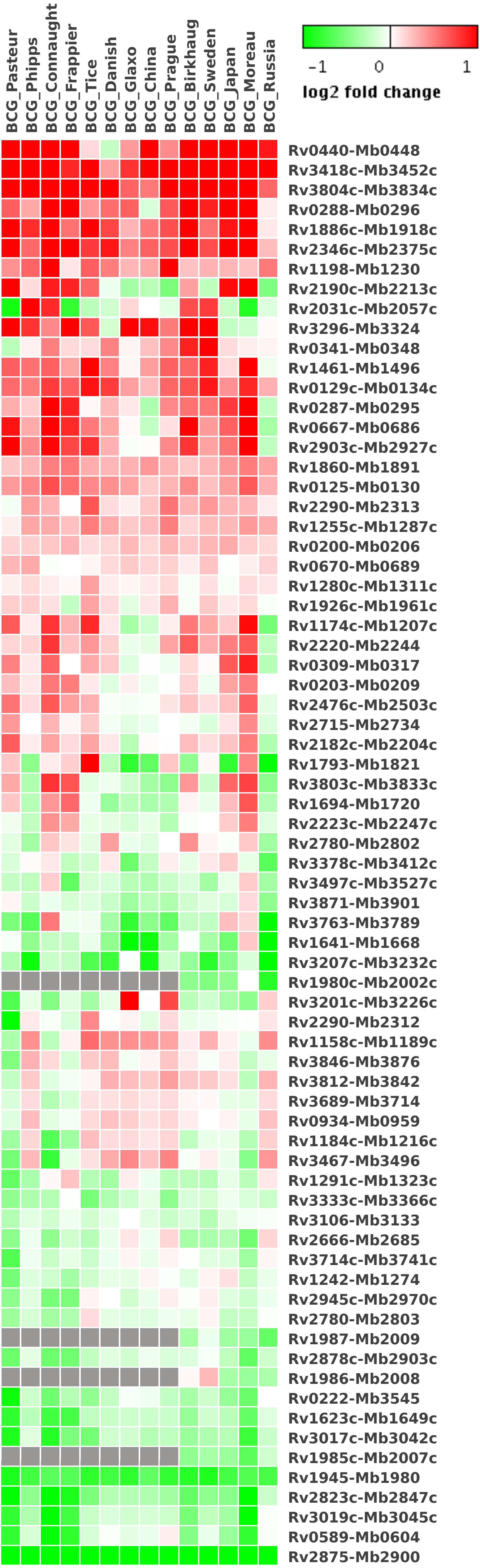
BCG_Pasteur
BCG_Phipps
BCG_Connaught
BCG_Frappier
BCG_Tice
BCG_Danish
BCG_Glaxo
BCG_China
BCG_Prague
BCG_Birkhaug
BCG_Sweden
BCG_Japan
BCG_Moreau
BCG_Russia
-1
0
1
log2 fold change
Rv0440-Mb0448
Rv3418c-Mb3452c
Rv3804c-Mb3834c
Rv0288-Mb0296
Rv1886c-Mb1918c
Rv2346c-Mb2375c
Rv1198-Mb1230
Rv2190c-Mb2213c
Rv2031c-Mb2057c
Rv3296-Mb3324
Rv0341-Mb0348
Rv1461-Mb1496
Rv0129c-Mb0134c
Rv0287-Mb0295
Rv0667-Mb0686
Rv2903c-Mb2927c
Rv1860-Mb1891
Rv0125-Mb0130
Rv2290-Mb2313
Rv1255c-Mb1287c
Rv0200-Mb0206
Rv0670-Mb0689
Rv1280c-Mb1311c
Rv1926c-Mb1961c
Rv1174c-Mb1207c
Rv2220-Mb2244
Rv0309-Mb0317
Rv0203-Mb0209
Rv2476c-Mb2503c
Rv2715-Mb2734
Rv2182c-Mb2204c
Rv1793-Mb1821
Rv3803c-Mb3833c
Rv1694-Mb1720
Rv2223c-Mb2247c
Rv2780-Mb2802
Rv3378c-Mb3412c
Rv3497c-Mb3527c
Rv3871-Mb3901
Rv3763-Mb3789
Rv1641-Mb1668
Rv3207c-Mb3232c
Rv1980c-Mb2002c
Rv3201c-Mb3226c
Rv2290-Mb2312
Rv1158c-Mb1189c
Rv3846-Mb3876
Rv3812-Mb3842
Rv3689-Mb3714
Rv0934-Mb0959
Rv1184c-Mb1216c
Rv3467-Mb3496
Rv1291c-Mb1323c
Rv3333c-Mb3366c
Rv3106-Mb3133
Rv2666-Mb2685
Rv3714c-Mb3741c
Rv1242-Mb1274
Rv2945c-Mb2970c
Rv2780-Mb2803
Rv1987-Mb2009
Rv2878c-Mb2903c
Rv1986-Mb2008
Rv0222-Mb3545
Rv1623c-Mb1649c
Rv3017c-Mb3042c
Rv1985c-Mb2007c
Rv1945-Mb1980
Rv2823c-Mb2847c
Rv3019c-Mb3045c
Rv0589-Mb0604
Rv2875-Mb2900

Figure S9

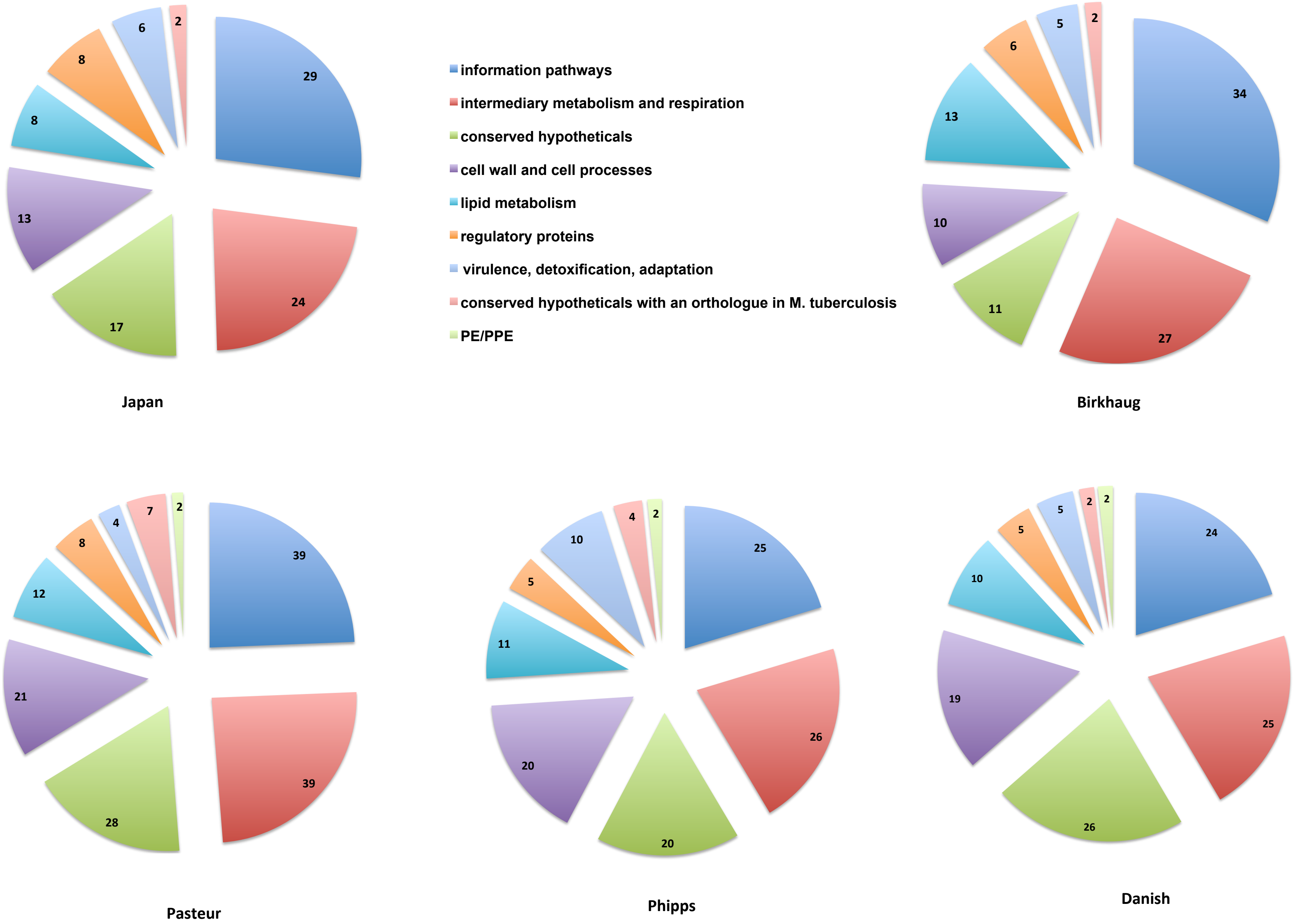
information pathways
intermediary metabolism and respiration
conserved hypotheticals
cell wall and cell processes
lipid metabolism
regulatory proteins
virulence, detoxification, adaptation
conserved hypotheticals with an orthologue in M. tuberculosis
PE/PPE
29
24
17
13
8
8
6
2
Japan
34
27
11
10
13
6
5
2
Birkhaug
39
39
28
21
12
8
4
7
2
Pasteur
25
26
20
20
11
5
10
4
2
Phipps
24
25
26
19
10
5
5
2
2
Danish

Figure S10
